# Suv4-20h Histone Methyltransferases Promote Neuroectodermal Differentiation by Silencing the Pluripotency-Associated Oct-25 Gene

**DOI:** 10.1371/journal.pgen.1003188

**Published:** 2013-01-31

**Authors:** Dario Nicetto, Matthias Hahn, Julia Jung, Tobias D. Schneider, Tobias Straub, Robert David, Gunnar Schotta, Ralph A. W. Rupp

**Affiliations:** 1 Adolf Butenandt Institut, Institut für Molekularbiologie, Ludwig Maximilians-Universität, München, Germany; 2 Center for Integrated Protein Science (Munich) at the Institut für Molekularbiologie, Adolf-Butenandt-Institut, LMU, München, Germany; 3 Medizinische Klinik I am Klinikum der Universität München (LMU), München, Germany; University of Cambridge, United Kingdom

## Abstract

Post-translational modifications (PTMs) of histones exert fundamental roles in regulating gene expression. During development, groups of PTMs are constrained by unknown mechanisms into combinatorial patterns, which facilitate transitions from uncommitted embryonic cells into differentiated somatic cell lineages. Repressive histone modifications such as H3K9me3 or H3K27me3 have been investigated in detail, but the role of H4K20me3 in development is currently unknown. Here we show that *Xenopus laevis* Suv4-20h1 and h2 histone methyltransferases (HMTases) are essential for induction and differentiation of the neuroectoderm. Morpholino-mediated knockdown of the two HMTases leads to a selective and specific downregulation of genes controlling neural induction, thereby effectively blocking differentiation of the neuroectoderm. Global transcriptome analysis supports the notion that these effects arise from the transcriptional deregulation of specific genes rather than widespread, pleiotropic effects. Interestingly, morphant embryos fail to repress the Oct4-related Xenopus gene Oct-25. We validate Oct-25 as a direct target of xSu4-20h enzyme mediated gene repression, showing by chromatin immunoprecipitaton that it is decorated with the H4K20me3 mark downstream of the promoter in normal, but not in double-morphant, embryos. Since knockdown of Oct-25 protein significantly rescues the neural differentiation defect in xSuv4-20h double-morphant embryos, we conclude that the epistatic relationship between Suv4-20h enzymes and Oct-25 controls the transit from pluripotent to differentiation-competent neural cells. Consistent with these results in Xenopus, murine Suv4-20h1/h2 double-knockout embryonic stem (DKO ES) cells exhibit increased Oct4 protein levels before and during EB formation, and reveal a compromised and biased capacity for *in vitro* differentiation, when compared to normal ES cells. Together, these results suggest a regulatory mechanism, conserved between amphibians and mammals, in which H4K20me3-dependent restriction of specific POU-V genes directs cell fate decisions, when embryonic cells exit the pluripotent state.

## Author Summary

The quest of modern developmental biology is a detailed molecular description of the process that leads from the fertilized egg to the complex and highly differentiated adult organism. This process is controlled largely on the level of gene expression. While early embryonic cells are pluripotent and capable of transcribing most of their genome, older cells have become committed to the germ layer and differentiation programs during gastrulation. They express then a subset of genes compatible with their future physiological function. Young, pluripotent cells and post-gastrula, committed cells express different networks of transcription factors and contain chromatin of different structure and composition. How these two regulatory layers are interconnected during development is incompletely understood. We describe a novel and unexpected link between the pluripotency-associated POU-V gene Oct-25 and xSuv4-20h histone methyltransferases. XSuv4-20h enzymes are required to repress the Oct-25 gene, a homolog of mammalian Oct4, in the neuroectoderm of frog embryos as a prerequisite for neural differentiation. Consistently, murine Suv4-20h double-null ES cells show increased Oct4 protein levels before and during *in vitro* differentiation and display compromised differentiation in comparison to wild-type ES cells. Thus, Suv4-20h enzymes control specific POU-V genes and are involved in germ-layer specific differentiation.

## Introduction

Embryonic development is controlled by fine-tuned differential gene expression. A succession of regulatory protein networks unfolds the zygotic gene expression program along a hierarchy of decisions, leading from the embryonic ground state to the epiblast and then to germ layers, which become patterned into cell type and organ precursor territories. The pluripotent trait, key feature of embryonic stem (ES) cells [1], is progressively restricted and finally lost as soon as embryonic cells become specified to germ layer fates. Recent studies have revealed that alterations in chromatin structure, dynamics and composition represent fundamental processes, which define the epigenetic landscape that directs cell type specification along this hierarchy [2], [3].

Besides important contributions from ATP dependent chromatin remodelling factors [4], [5] and histone variants [6] in modulating nucleosome dynamics, histone post-translational modifications (PTMs) have been linked to gene expression [3], [7]. The transition from pluripotent to differentiated cells is characterized by a progressive increase in heterochromatin formation, in a process that changes the hyperdynamic open chromatin structure into a less accessible architecture [1], [8]. At the same time transcriptional silencing of non-lineage specific genes is achieved via acquisition of repressive histone marks. *In vivo* studies have shown that dynamic alterations in the levels of histone modifications characterize early stages of development both in mammals [9]–[11] and other vertebrates [12]–[14].

Lysine methylation of histones is catalyzed by SET domain-containing histone methyltransferases (HMTases), and can be linked both to transcriptional activation and repression [15], [16]. In particular, repressive histone methyl marks are found on lysine residues at position 9 and 27 on histone H3 and in position 20 on histone H4. H3K27 trimethylation is catalyzed by polycomb repression complex (PRC) 2, which predominantly represses developmental regulatory genes [17]–[19]. Trimethylation of H3K9 and H4K20 relies on Suv39h and Suv4-20h enzyme activities, respectively [20], [21], and predominantly marks repetitive genomic DNA at pericentromeric and telomeric heterochromatin [16], [21]. While H3K9-specific HMTases have been characterized in significant depth [20], [22], [23], we know little about the functions of Suv4-20h1 and Suv4-20h2 enzymes with regard to gene regulation. *In vivo* analysis of H4K20 methylation states in mouse embryos reveals specific patterns both in cellular or subnuclear abundance [9], [24]. Suv4-20h DKO pups die perinatally, indicating an essential function of the two enzymes during embryogenesis [24]. Moreover, quantitative analysis of histone PTMs in *X. laevis* revealed a progressive and significant accumulation of H4K20me3 levels during embryogenesis, suggesting developmental functions for these enzymes [13].

To characterize the functional role of H4K20me2/3 during vertebrate development we have investigated the consequences of both morpholino-mediated protein knockdown and mRNA-born overexpression of the Xenopus Suv4-20h1 and h2 homologs in frog embryos. Our data reveal a specific and selective requirement for Suv4-20h enzyme acitivities in neuroectodermal differentiation, in a process which involves transcriptional repression of pluripotency associated POU-V genes, both in Xenopus embryos and in murine ES cells.

## Results

### Characterization of Xenopus Suv4-20h1 and h2 enzymes

We initially identified *X. laevis* Suv4-20h1 and h2 ESTs via database mining. Mouse and frog Suv4-20h1 and h2 protein sequences are well conserved, particularly within the SET domains (≥88% identity), even though the xSuv4-20h2 open reading frame is longer than its mouse homolog due to C-terminal insertions (supplementary data, Figure S1). XSuv4-20h1/h2 genes are both maternally and zygotically expressed in a ubiquitous manner, as shown by RNA *in situ* hybridisation and RT-PCR analysis (Figure S2A–S2D). XSuv4-20h1 mRNA abundance decreases during the initial stages of development and subsequently rises from mid-gastrula on, reflecting the switch from maternal-to-zygotic transcription at midblastula. In contrast, the initially high xSuv4-20h2 mRNA level falls and stays low at late stages (Figure S2D).

**Figure 1:**
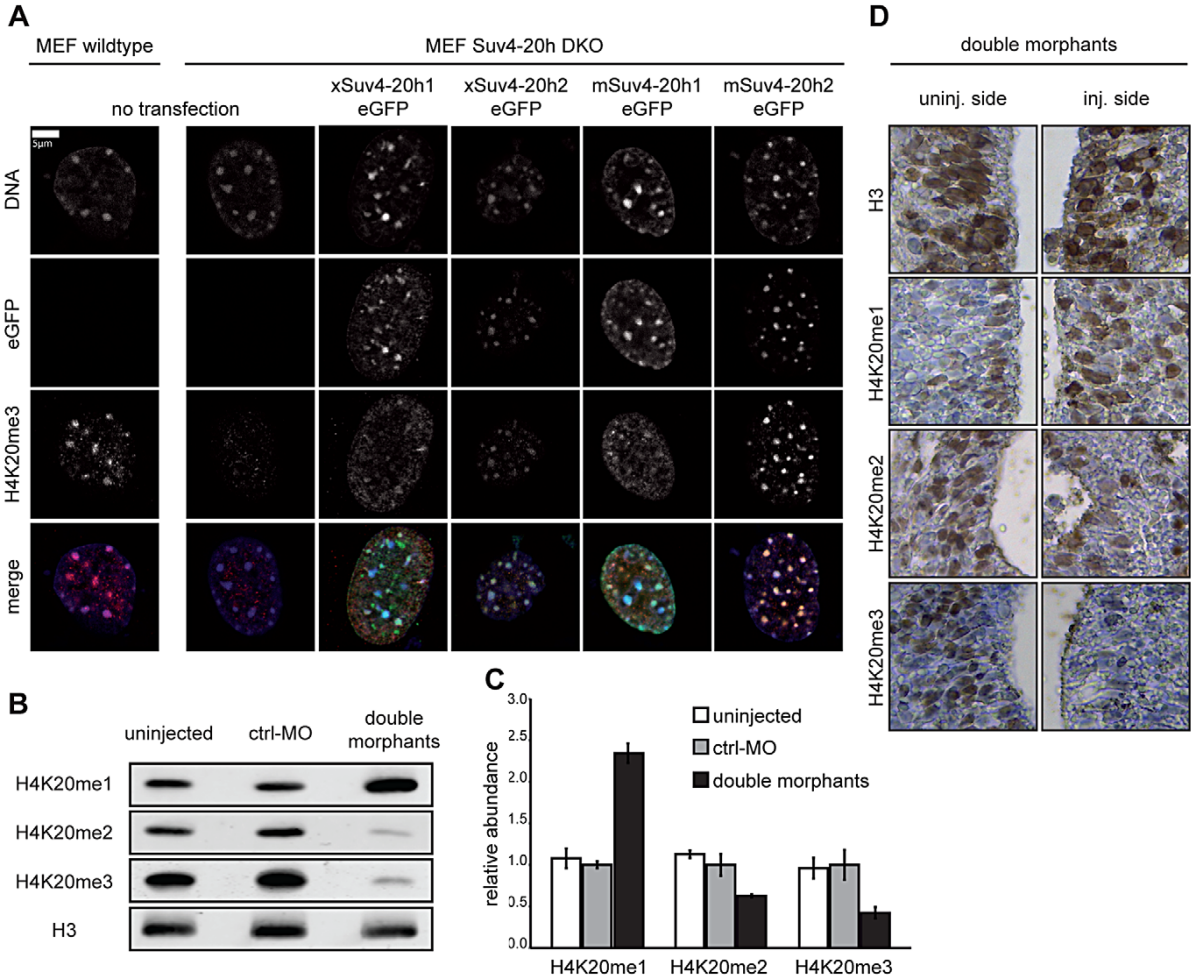
Functional analysis of xSuv4-20h HMTases. (A) Transiently transfected eGFP-tagged Suv4-20h1 and h2 enzymes from frog or mouse re-establish H4K20me3 marks in heterochromatic foci of Suv4-20h1/h2 DKO MEFs. (B–D) Bulk histones from tadpoles (NF30-33) injected with morpholinos targeting translation of endogenous xSuv4-20h1 and h2 mRNA show a strong reduction in H4K20me2 and H4K20me3 levels and a concomitant increase in the H4K20me1 mark. (B) Western Blot analysis of uninjected embryos, control morphants (ctrl-MO), and double morphants with antibodies against H4K20 mono-, di- and trimethylation. PanH3 antibody was used as loading control. (C) Western Blot quantification of three independent biological experiments; data represent mean values, error bars indicate SEM. (D) Immuno-histochemistry on xSuv4-20h double morphant tadpoles. Panels show details from neural tubes stained with antibodies against the histone epitopes indicated on the side. Whole sections shown in Figure S5A.

To test the acivities of these Xenopus HMTases, we first analyzed their ability to rescue H4K20me3 levels in Suv4-20h1/h2 DKO mouse embryonic fibroblasts (MEF Suv4-20h DKO; [24]), which lack this modification. Both frog cDNAs re-established a proper H4K20me3 pattern, which was strongly enriched at heterochromatic regions that were identified as DAPI-dense chromocenters within nuclei (Figure 1A). Thus, Xenopus laevis Suv4-20h homologs are biologically active and can direct H4K20 trimethylation. To test, whether they generate this histone mark in frog embryos, we designed antisense Morpholino oligonucleotides (MO) to reduce synthesis of xSuv4-20h1 and h2 proteins from endogenous mRNAs (Figure S3A, S3B). These MOs specifically inhibited translation of their cognate templates *in vitro* (Figure S3C). To avoid possible functional complementation between the xSuv4-20h enzymes *in vivo*, we decided to inject the two MOs simultaneously into both blastomeres of 2-cell stage embryos and performed western blots with nuclear protein extracts from these double-morphant embryos at the tadpole stage (NF30-33). Compared to uninjected controls or embryos injected with an unrelated control MO (control-morphants), the double morphants contained significantly less H4K20me2 (p = 0.0011) and H4K20me3 (p = 0.0164), which was coupled to an increase in H4K20me1 (p = 0.0034) (Figure 1B and 1C).

This result was confirmed by MALDI-TOF mass spectrometry (Figure S4A). As described in Schneider et al. [13], the relative abundance of histone modifications was calculated by quantifying the amount of a specific modification relative to the amount of all modification states determined for the same histone peptide. As reported before [13], the H4K20me3 mark could not be quantitated reproducibly for technical reasons. Compared to control embryos, however, xSuv4-20h double morphants contained approximately 2.5-fold less of H4K20me2 (p = 0.0153) and three-fold more H4K20me1 (p = 0.0185), while the abundance of the unmodified peptide state remained unaffected. Importantly, the levels of histone H3 methylation on two tryptic peptides, covering the K9, K27 and K36 positions, were indistinguishable between control and double-morphant embryos (Figure S4B and S4C). Western blot analysis with antibodies against trimethylated H3K9 or H3K27 also showed no difference in the abundance of these two marks between control embryos and xSuv4-20h double morphants (Figure S4D).

To further characterize the effects of xSuv4-20h enzyme depletion on the cellular level, we performed immunohistochemistry on sections from tailbud stage embryos (NF30), which were injected with the xSuv4-20h MO-mix into one of two blastomeres at 2-cell stage together with fluorescently labelled dextranes as lineage tracer. While H3 staining was comparable between injected and uninjected sides under all conditions (Figure S5A), staining for H4K20me2 and –me3 was clearly reduced on the double-morphant side of the neural tube (Figure 1D). In agreement with our western blot and mass spec results, the reduction in the di- and tri-methyl mark was coupled to an increase in H4K20me1 staining. Altogether these results indicate that xSuv4-20h1 and h2 downregulation leads to a quantitative reduction of H4K20 di- and trimethyl marks in the frog embryo, without affecting the bulk abundance of other repressive histone marks such as H3 K9/K27 methylation.

RNA-based overexpression of Suv4-20h HMTases had the opposite effect. When injected singly, xSuv4-20h1 or h2 mRNAs increased both di- and trimethylated H4K20 in a dose-dependent manner (Figure S8A). A comparable result was achieved by injection of either mouse Suv4-20h1 or h2 mRNAs (Figure S9A). Together, these results identify the frog cDNAs as orthologs of mammalian Suv4-20h enzymes. Loss and gain of function experiments also indicate that the bulk abundance of di- and trimethylated H4K20 can be manipulated over a wide range without compromising embryonic viability.

### XSuv4-20h HMTases depletion inhibits eye and melanocyte formation

**Figure 2:**
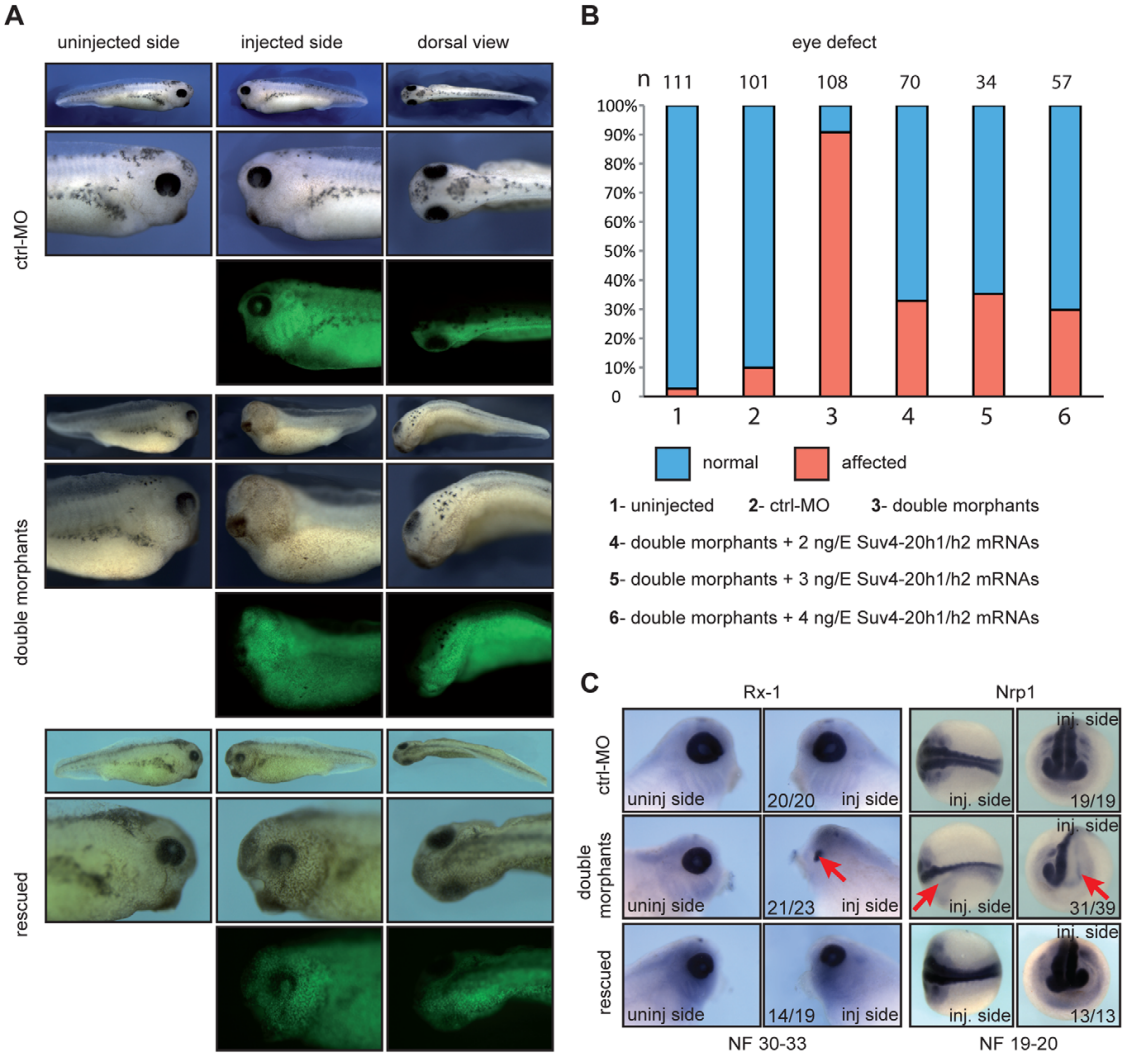
xSuv4-20h1/h2 double morphants lack eyes and melanophores. (A) Morphological phenotypes of representative tadpoles (NF30-33) from embryo cohorts injected into one of two blastomeres at two-cell stage with ctrl-MO, xSuv4-20h1/h2 MOs (double morphants), and double morphants coinjected with 3 ng mouse Suv4-20h1/h2 mRNAs (rescued). Injected body halves were identified by green fluorescence of the coinjected lineage tracer Fluor 488 Dextran. (B) Penetrance of the eye phenotype. Data from three to five independent experiments; n = total number of embryos scored. (C) RNA *in situ* hybridization analysis for Rx-1 in tadpoles (NF30-33), and CNS markers Nrp1 at neural tube stage (NF19-20). For each condition, numbers refer to embryos showing the displayed morphology or staining, in comparison to the total number of analysed embryos.

We next tested, whether depletion of xSuv4-20h HMTases affects embryonic development. We injected xSuv4-20h1/h2 MO-mix into one blastomere of two-cell stage embryos and scored phenotypic alterations by comparing injected with uninjected sides. No obvious differences were observed during early development, including gastrulation, axial extension and dorsoventral patterning. From tailbud stages on, two main phenotypes became manifest. First, in the injected side of xSuv4-20h double morphants the eye formation was strongly compromised. The eye rudiments contained no or barely visible retinal pigment and typically had no lens (Figure 2A). Secondly, melanophores that are found on the dorsal part of the head and the lateral portion of the trunk, were severely reduced in numbers or completely lost from the double-morphant side (Figure 2A). Both phenotypes had a penetrance between 80–90% in xSuv4–20h double morphants (p<0.0001, Fisher's exact test) in several independent experiments (Figure 2B). Control-morphant embryos had normal eyes and melanocytes (Figure 2A) and were indistinguishable from uninjected siblings in most cases (Figure 2B).

The distinct eye phenotype prompted us to investigate the underlying molecular changes. RNA *in situ* hybridization experiments revealed a clearly reduced expression of the homeobox transcription factor Rx-1 (Figure 2C) and the paired box transcription factor Pax-6 (Figure S5D) in xSuv4-20h double morphants. The reduction of these two master regulators of eye differentiation explains the morphological eye phenotype, but we noticed that embryonic transcription was already misregulated upstream of these factors. The pan-neural markers Nrp1 (Figure 2C) and N-CAM (Figure S5E), which are induced during gastrula stages, were also strongly reduced in double morphants. However, several key markers of embryonic patterning were not perturbed, such as the organizer genes Chordin, Goosecoid and Xnr-3 at gastrula stages (Figure S5B). The anteroposterior patterning of the central nervous system (CNS) appeared also to be normal given the wild-type-like expression patterns of Otx2 and Krox20 in fore- and hindbrain territories, respectively (Figure S5C). These results provide first evidence that H4K20 di- and trimethylation serves to regulate distinct developmental genes in a selective manner.

### Xenopus Suv4-20h activity is required for normal development

The specificity of the developmental phenotypes arising from xSuv4-20h enzyme depletion was validated by rescue experiments, in which we coinjected increasing doses of murine Suv4-20h1/h2 mRNAs together with the xSuv4-20h MO-mix. Due to sequence divergence, transcripts of the murine orthologs escape inhibition by the MOs targeting the frog mRNAs. Already 2 ng of murine Suv4-20h transcripts were sufficient to rescue the eye defect in two thirds of the double morphant embryos (p<0.0001, Fisher's exact test). In most cases, the retinal neuroepithelium regained its circular structure and near normal size, as well as a central lens (Figure 2A). The rescue efficiency did not increase with higher concentrations of mouse transcripts (Figure 2B, columns 4–6). The number of melanophores was also increased at their proper sites under rescue conditions (Figure 2A). Furthermore, the expression domains of Rx-1 and Nrp1 (Figure 2C), as well as Pax-6 and N-CAM (Figure S5D and S5E) were efficiently restored.

To test, whether this phenotypic rescue requires Suv4-20h proteins or their enzymatic activity, we generated catalytically inactive murine Suv4-20h protein variants (Figure S6A), based on structural predictions [25], [26]. Unlike the wild-type proteins, neither variant restored the H4K20me3 mark at heterochromatic foci in Suv4-20h DKO MEFs (Figure S6B). When tested side by side with the wild-type enzymes, the mutants did neither increase the abundance of the H4K20me2 and -me3 marks in wild-type frog embryos (Figure S6C, compare lanes 1, 3 and 5), nor rescue H4K20 methylation levels in xSuv4-20h double morphants (Figure S6C compare lanes 1, 4 and 6), although being expressed at similar levels (Figure S6D). Consequently, the inactive variants also failed to rescue the eye and melanophore phenotype (Figure S7A–S7C and S7D, compare columns 2–4).

In the course of these experiments we noticed that overexpression of either frog or mouse Suv4-20h1 and h2 proteins never caused any obvious morphological or molecular changes in the embryos (Figures S8B, S8C and S9B, S9C), despite strongly enhanced H4K20me3 levels in bulk chromatin (Figures S8A and S9A). In particular, morphological landmarks such as eyes and melanophores formed normal in size, number and location under overexpression conditions. Expression domains of marker genes such as Rx-1 and Pax-6 were unaffected (Figures S8D and S9D). Thus, H4K20 di- and trimethylation is required for normal development, but excess deposition of these marks has no apparent phenotypic consequences.

### XSuv4-20h enzymes are required for ectoderm formation

**Figure 3:**
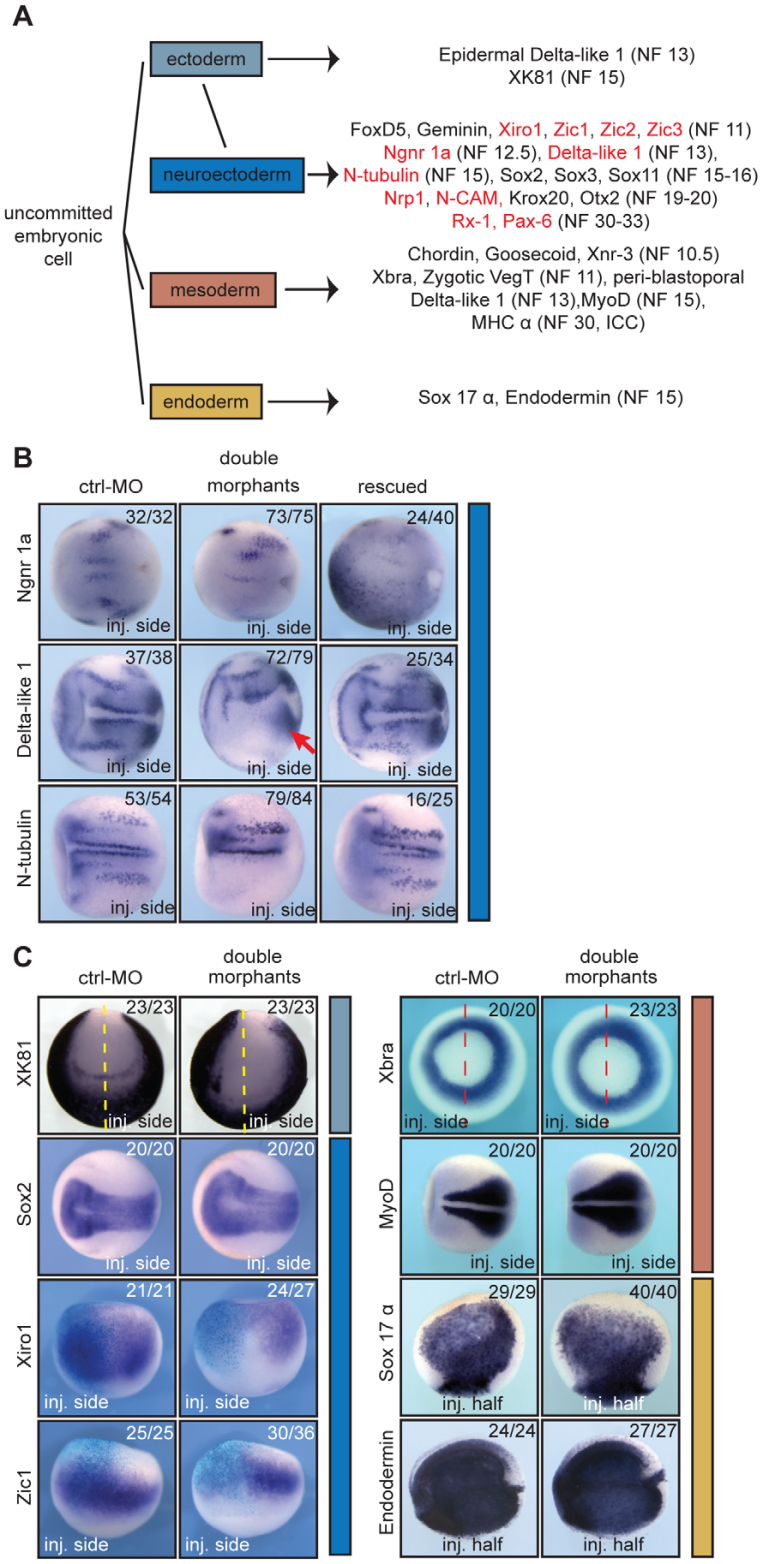
xSuv4-20h enzymes are required for differentiation of the neuroectoderm. (A) Schematic illustration of analysed markers of the different germ layers (germ layer colour code extended to *in situ* panels). Downregulated genes upon xSuv4-20h depletion are labelled in red. (B) Expression pattern of the neuroectodermal markers Ngnr 1a (NF12.5), Delta-like 1 (NF13), and N-tubulin (NF15). The pictures show dorsal views of the open neural plate with anterior to the left. (C) Expression patterns of XK81 (ectoderm), Sox2, Xiro1, Zic1 (neuroectoderm), Xbra, MyoD (mesoderm), Sox17 α, Endodermin (endoderm) in ctrl-MO injected or double morphant embryos. XK81 - anterior views with dorsal side to the top. Sox2 and MyoD - dorsal views, anterior to the left. Xiro1 and Zic1 - dorsal views; injected halves are lineage-traced by coinjection of LacZ mRNA and subsequent β–Gal staining (light blue). Xbra - vegetal view. Sox-17 α - internal stain from the injected side in bisected embryos, animal pole up. Endodermin – internal stain from the injected side in bisected embryos; anterior to the left.

The apparent functional selectivity of the ubiquitously expressed enzymes encouraged us to test, whether xSuv4-20h HMTases control additional aspects of germ layer formation and patterning. Therefore, we compared the expression of key developmental regulatory genes in uni-laterally injected control-morphants *versus* xSuv4-20h double morphants by RNA *in situ* hybridisation (listed in Figure 3A).

Based on our previous results, we continued with genes involved in neurogenesis (Figure 3B). At the open plate stage, primary neurons are specified in three stripes next to the dorsal midline on each side. At this time, each stripe expresses the neural specific regulatory genes Neurogenin-related 1a (Ngnr-1a) and Delta-like 1, as well as the differentiation marker N-tubulin. The expression of these three genes was extinguished in almost all of the xSuv4-20h MO-injected sides, while being unaffected by control-MO (Figure 3B). In addition to these stripes, Delta-like 1 mRNA delineates the anterior border of the neural plate, and this domain was also extinguished (Figure 3B). In contrast, mesodermal expression of Delta-like 1 around the blastoporus remained unaffected in morphant condition (Figure 3B, arrow). Delta-like 1 and N-tubulin stripes were effectively rescued by coinjection of wild-type mSuv4-20h1/h2 mRNAs, while Ngnr-1a was restored in a broad, diffuse manner (Figure 3B, right column). Notably, inactive mouse Suv4-20h HMTases could not rescue N-tubulin expression (Figure S7E, middle column). At the same time, mesodermal control genes like MyoD were unaffected (Figure S7E, right column) Together, these results implicate xSuv4-20h enzymes in neuronal fate selection.

Next, we extented our analysis to marker genes expressed in other germlayers and territories (Figure 3C and Figure S5). The epidermal keratin gene XK81 demarcates non-neural ectoderm and was expressed normally on the surface of morphant epidermis; however, due to a slight retardation in neural tube closure on the injected side, its expression appears asymmetric in anterior views. This may indicate an involvement of xSuv4-20h enzymes in morphogenetic processes during neurulation and/or neural crest specification. This phenotype led to a mild broadening of the neural plate markers Sox2 (Figure 3C), Sox3 and Sox11 (Figure S5F) at apparently normal mRNA levels. Prior to these neural plate markers, a group of genes including FoxD5, Geminin, Zic1, Zic2, Zic3 and members of the Iroquois family are induced in the prospective neuroectoderm and stabilize the neural fate by their regulatory interactions (reviewed in ref [27]). At midgastrula (NF11), FoxD5 and Geminin did not respond to xSuv4-20h enzyme depletion (Figure S5F), but Xiro1, Zic1 (Figure 3C), Zic2 and Zic3 (Figure S5F) mRNAs were strongly reduced. In contrast, key mesodermal factors such as Xbra, MyoD (Figure 3C) and VegT (Figure S5F), as well as regulators of endodermal differentiation like Sox17 α and Endodermin (Figure 3C) were expressed normally in both morphants and in embryos overexpressing frog xSuv4-20h proteins (Figure S8E). Taken together these results demonstrate that xSuv4-20h HMTases are critical for neural development, but apparently dispensable for mesoderm and endoderm formation in *X. laevis*.

**Figure 4:**
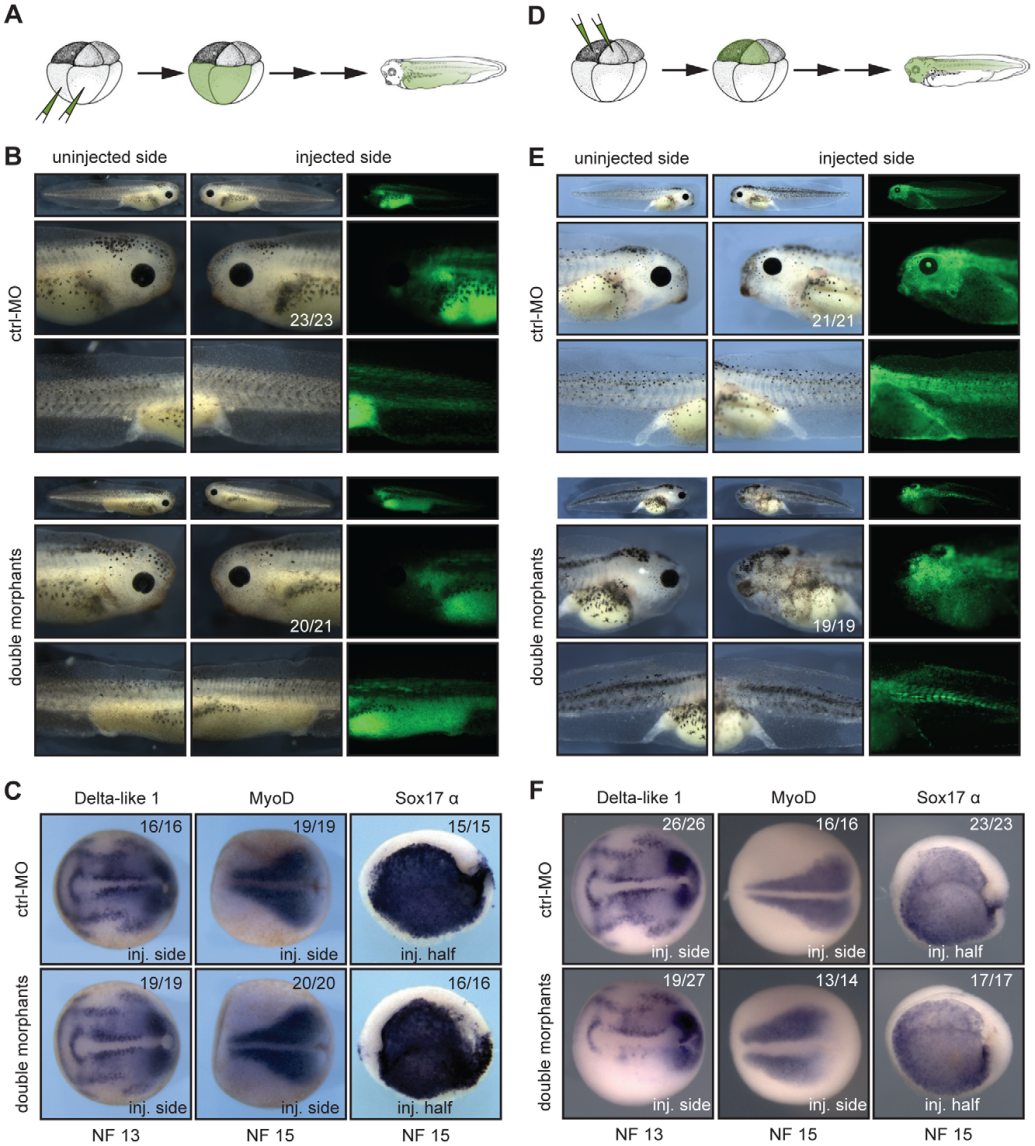
xSuv4-20h1/h2 enzymatic activity is required in the ectodermal germ layer. (A, D) Schematic illustrations of targeting microinjections into mesendodermal or ectodermal territories at 8-cell stage. (B) Injecting xSuv4-20h MOs into the mesendoderm causes no apparent morphological phenotype in the embryo. (C) Neural, mesodermal and endodermal marker genes are expressed normally. (E) xSuv4-20h MOs reduce eyes, cranial and trunk melanophores, when injected into the ectoderm. (F) Expression of all tested markers in mesoderm and endoderm is normal, except for Delta-like 1, whose expression specifically in the open neural plate is strongly reduced on the injected side. Global morphology was assessed at hatching stage (NF36), molecular markers at indicated stages during neurulation. Top row images in (B) and (E) depict whole embryos for overview.

To further verify the specific role of Xenopus Suv4-20h enzymes in neural development, we considered two different approaches; in a first series of experiments we performed injections at 8-cell stage in the animal or vegetal pole blastomeres, selectively labelling cells predominantly belonging either to mesendoderm (vegetal injections, Figure 4A) or ectoderm (animal injections, Figure 4D). Vegetal pole blastomere injections led to no evident morphological and molecular phenotypes (Figure 4B and 4C). Conversely, animal injections reproduced the eye and melanophore phenotypes from half-injected embryos, while mesodermal and endodermal structures developed normally (Figure 4E). Consistent with the morphological defects, Delta-like 1 expression in the neural plate was suppressed, while MyoD and Sox17 α genes were unaffected (Figure 4F). These results provide strong evidence that the neural and melanocyte phenotypes originate in the ectoderm.

**Figure 5:**
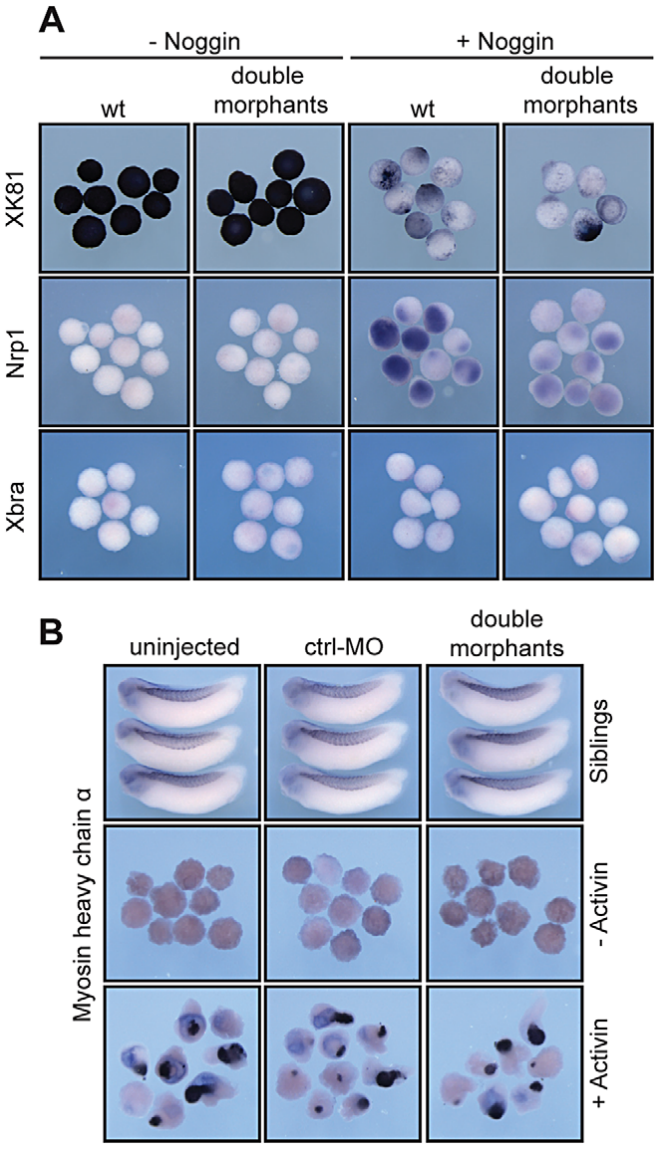
*In vitro* induction of xSuv4-20h double-morphant animal cap explants. (A) Noggin-dependent neuralisation. XK81, Nrp1 and Xbra expression is monitored in uninjected control caps and double-morphant caps with or without Noggin mRNA. Note that explants coinjected with xSuv4-20h MOs together with Noggin mRNA show reduced Nrp1 expression, but normal downregulation of XK81 mRNA. (B) Muscle induction by Activin A in uninjected, ctrl-MO injected, and xSuv4-20h double morphant animal caps. Top row demonstrates comparable expression of myosin heavy chain (MHC-α) in non-dissected sibling embryos.

As second approach we took advantage of animal cap (AC) explants, which form epidermis in isolation but can be neuralized by the BMP-inhibitor Noggin. Specifically, we tested whether the downregulation of xSuv4-20h HMTases prevented neural induction by Noggin. Without Noggin, wt and double morphant explants were positive for XK81 and negative for Nrp1 (Figure 5A). They were also negative for Xbra, indicating absence of contaminating mesoderm. Noggin-mediated Nrp1 expression was clearly visible in wt caps, but strongly reduced upon co-injection of xSuv4-20h morpholinos, while XK81 expression was downregulated in both the samples (Figure 5A). Thus, double morphant caps are both refractory to neural induction and restrained in epidermal differentiation. However, they differentiate into mesoderm upon stimulation with Activin A just like control explants, as shown by immunostaining for muscle myosin heavy chain (Figure 5B). These results confirm the crucial role of xSuv4-20h enzymes in coordinating the formation of ectodermal tissues, and show that in the absence of the two enzymes neural induction is impaired.

### XSuv4-20h enzymes are required for cell survival and proliferation

Loss of H4K20 di- and trimethylation is known to compromise DNA damage repair in mice and to partially block G1/S transition [24]. This prompted us to test, whether xSuv4-20h depletion affects apoptosis and cell proliferation in frog embryos. Immunostaining for activated Caspase3 revealed an increase in apoptotic cells on the injected side of double morphant embryos (Figure S10A). Coinjection of xBcl-2 mRNA, an anti-apoptotic factor, reduced the Caspase3 positive cells to levels of the uninjected control side, however, without re-establishing a proper Delta-like 1 and N-tubulin pattern in the double-morphant side. Overexpression of xBcl-2 mRNA alone had no effect on the expression of the tested markers (Figure S10A). Thus, although embryonic frog cells depleted for the H4K20me2/me3 marks become apoptotic at higher rate than wt cells, the absence of neurons in the double-morphant neural plate cannot be explained by selective cell death.

Double morphant embryos stained for the mitotic marker H3S10P, showed a two-fold reduction (p = 0.0058) in the number of proliferating cells at midneurula stage, compared to control morphant embryos (Figure S10B). This mild phenotype might be correlated with the observed increase in apoptosis. Since neural induction continues in frogs, even when cell proliferation is blocked from gastrulation onwards [28], it is unlikely that the nearly complete loss of N-tubulin positive neurons is brought about by this mild reduction in cell proliferation. Taken together, the main xSuv4-20h morphant phenotype represents not a selective loss of neuroblasts, but a block in neural differentiation.

### XSuv4-20h double morphant frog embryos fail to silence Oct-25 transcription in sensorial ectoderm

**Figure 6:**
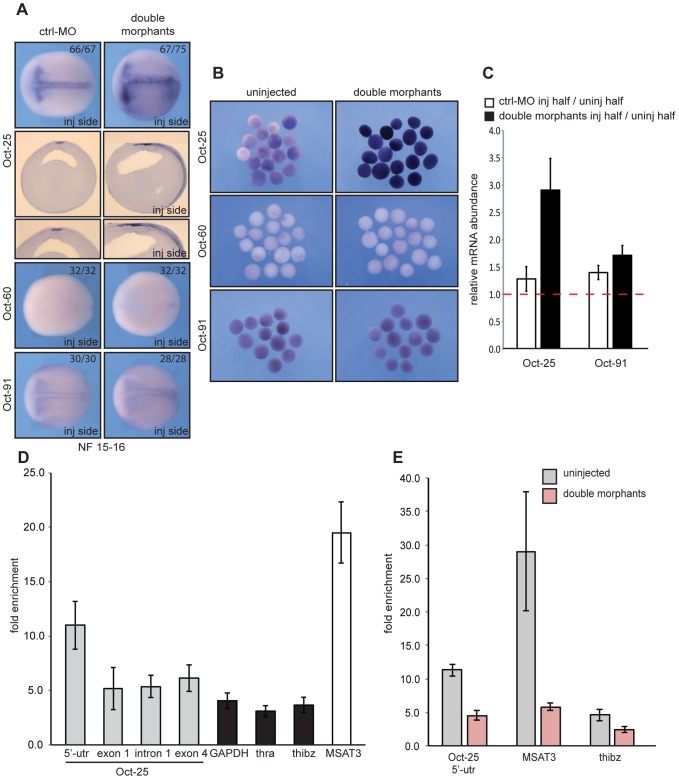
xSuv4-20h double morphants fail to silence Oct-25 expression in deep-layer ectoderm due to reduced H4K20me3 enrichment at Oct-25 promoter. RNA *in situ* hybridization analysis for Oct-25, Oct-60 and Oct-91 in embryos (A) and animal caps (B) for ctrl-morphants or xSuv4-20h double-morphant embryos, injected unilaterally at two-cell stage and fixed at midneurula stage (NF15). Injected sides were defined by coinjected Alexa-fluorescence prior to *in situ* hybridisation. (A) Dorsal views of stained embryos with anterior to the left. For Oct-25 Vibratome cross-sections, ctrl-MO and double-morphant embryos are shown. (B) Comparative expression analysis for Oct-25, Oct-60 and Oct-91 in animal caps from bilaterally injected embryos, fixed at midneurula stage (NF15). (C) qRT-PCR profiles for Oct-25 and Oct-91 in ctrl-MO and xSuv4-20h double-morphant embryos. Data represent normalized ratios of mRNA levels as means of four independent experiments, error bars indicate SEM. (D, E) Chromatin immunoprecipitation (ChIP) analysis on Oct-25, GAPDH, thra, thibz genes and major satellite repeat region 3 (MSAT3). (D) H4K20me3 levels on the indicated genes in normal embryos, and (E) in uninjected *versus* double-morphant embryos. Fold enrichment was calculated as the ratio between H4K20me3 precipitated material over negative control (No antibody sample). Data represent mean values of three to five independent experiments, error bars indicate SEM.

So far, our analysis in xSuv4-20h morphant embryos has indicated a specific and selective loss of gene expression in ectodermally derived tissues. The earliest affected markers - Zic and Xiro genes - become induced at early gastrula stage and help establish the neural plate state [27]. At this time in frog development, embryonic cells in the animal hemisphere are still plastic and express members of the POU-V gene family – i.e. Oct-25, Oct-60 and Oct-91 - that encode paralogs of the mammalian pluripotency regulator Oct4 [29], [30]. Because Oct-25 and Oct-91 regulate germ layer differentiation in Xenopus [31]–[34], we investigated their expression (Figure 6A). Oct-25 is initially expressed throughout the animal hemisphere at early gastrula, but gets restricted to the presumptive floor plate (notoplate) by midneurula [31]. On the injected side of the vast majority of double morphants, however, Oct-25 expression was expanded from the notoplate down to the ventral midline. Interestingly, ectopic Oct-25 expression was restricted to the sensorial cell layer of the ectoderm, which contains neural and epidermal precursor cells, respectively (Figure 6A, sections). The Oct-60 gene, which is expressed during oogenesis, was not activated under these conditions. Oct-91 staining appeared normal in the majority of the embryos, although some showed a mild upregulation in double morphants as well (data not shown). The ectopic expression of Oct-25 is a specific consequence of xSuv4-20h enzyme depletion, because its normal pattern was re-established in morphants upon coinjection of mRNAs encoding wild-type, but not inactive, mouse Suv4-20h proteins (Figure S7E, left column). Notably, the selective derepression of the Oct-25 gene was also observed in double-morphant AC explants (Figure 6B), excluding indirect effects from non-ectodermal tissues.

We then performed qRT-PCR analysis to quantitate the relative changes in gene expression. It is frequently observed that embryo cohorts develop in slight asynchrony as a non-specific consequence of Morpholino injection, possibly obscuring transcriptional responses. To minimize this potential artifact, we analysed the RNAs of matching pairs of wt and xSuv4-20h depleted samples by dissecting embryos at early neurula stage (NF14) into uninjected and injected halves, based on the coinjected fluorescent lineage tracer (Figure S11A). As shown in Figure 6C, the Oct-25 mRNA is about three-fold higher in xSuv4-20h double-morphant halves (p = 0.0123), while being similar between control-morphant and uninjected halves. In the same sample, Oct-91 expression was unaffected (Figure 6C). We used this assay also to confirm the diminished expression of neural plate marker genes detected earlier by RNA *in situ* hybridisation. With the exception of Ngnr 1a, Nrp1 and N-tubulin, mRNA levels were clearly reduced in the morphant halves (p = 0.0122 and 0.0163, respectively; Figure S11B).

To gain further information about the complexity of the underlying transcriptional misregulation, we performed transcriptome analysis in wild-type and double-morphant embryos, again dissecting embryos in corresponding pairs of injected and uninjected halves (Figure S12A). Six percent of the 11639 annotated probe sets present on the microarray were significantly altered in their expression as a consequence of xSuv4-20h enzyme depletion, about equally split into upregulated (n = 319) and downregulated (n = 404) probes (Figure S12B and S12C; for a complete list of the responding probesets see NCBI's GEO Series accession number GSE41256). This result suggests that the observed phenotypes in the double morphants originate from transcriptional misregulation of distinct genes, rather than from global, pleiotropic effects. Indeed, Oct-25 mRNA is also specifically upregulated in the microarray data set, where it is among the ten most upregulated mRNAs in the double-morphant condition (Figure S12D).

The sustained expression of Oct-25 in xSuv4-20h morphant embryos fits the prediction of Oct-25 being a direct target of H4K20me3 mediated transcriptional silencing. To test this assumption directly, we carried out chromatin-immunoprecipitation (ChIP) experiments with H4K20me3-specific antibodies at the neurula stage (NF15-16). For ChIP experiments we used *X. tropicalis* embryos, since the available genome sequence of this closely related frog species [35] allowed us to design primer amplicons for non-exon derived DNA sequences. RNA *in situ* hybridization performed on neurula stage *X. tropicalis* embryos, confirmed that the expression patterns of Oct-25 and N-tubulin were up- and down-regulated, respectively, to the same extend as observed for X. laevis (Figure S13). We retrived the pericentromeric major satellite repeat sequence (MSAT3) as positive control amplicon for the experiment. Genic regions, which are H4K20me3-free and, thus, could be used as negative controls, are difficult to predict, since genome-wide analysis in mammalian cells reported only enrichment of this modification on pericentromeric and subtelomeric heterochromatin [36], [37]. As negative controls we considered: GAPDH, a constitutively expressed housekeeping gene; thyroid hormone receptor α (thra), a gene whose expression can be detected at neurula; and thra-induced bzip protein (thibz) that is expressed from metamorphosis on (Figure S14A). Statistical analysis of qRT/PCR data indicates that expression of GAPDH and thra was not significantly altered under the double-morphant condition (). Therefore, the relative H4K20me3 levels at these genes were defined as background, and compared to the levels on other loci (Figure S14A). The modification strongly decorated the pericentromeric MSAT3 repeat region (Figure 6D), as expected from the analysis in murine cells [21]. At the 5′UTR amplicon of the Oct-25 gene, H4K20me3 was significantly enriched compared to the control genes GAPDH (p = 0.0155), thra (p = 0.0103) and thibz (p = 0.0128) (Figure S14A and Figure 6D). In a second set of experiments, we compared the abundance of H4K20me3 between wild-type and xSuv4-20h double-morphant embryos (Figure 6E). In morphants, the modification was selectively reduced at the 5′UTR amplicon of Oct-25 (p = 0.004). Together, these ChIP experiments validate the 5′ end of the Oct-25 gene as direct target of xSuv4-20h mediated transcriptional silencing.

Xenopus Oct-25 has been implicated in germ layer formation [32], [34]. We wanted to know, whether the sustained expression of Oct-25 in xSuv4-20h morphants could cause the observed downregulation of early neural plate and neural differentiation markers. This question is difficult to address, since the role of Oct-25 in neural induction is ambiguous - both overexpression and morpholino knockdown inhibit neural differentiation [32], [34]. Thus, Oct-25 acts in pleiotropic fashion, perhaps switching target genes or protein interaction partners. In a previous report [38], human Oct4 protein was shown by ChIP analysis to bind to promoters of early neural markers, including Zic and Sox genes. In order to link Xenopus Oct-25 mechanistically to these genes, we have misexpressed constitutively activating and repressing Oct-25 fusion proteins in animal caps (Figure S15A). Zic1, Zic3 and Sox2 responded to the Oct-25 variants in a manner consistent with direct regulator/target gene interaction, i.e. they were hyperactivated by Oct-25-VP16 (p = 0.0143; 0.0456; 0.01622, respectively) and suppressed by Oct-25-EnR (p = 0.0236; 0.0167; 0.0231, respectively) compared to the uninjected sample. In line with this assumption, inspection of the *X. tropicalis* gene sequences detailed the presence of multiple Oct-25 DNA binding motifs within 2.0 Kb distance from the transcriptional start site for each of these genes (Figure S16). For the two Zic genes, which are misregulated in the forming neural plate of morphant embryos (Figure 3C and Figure S5F), we confirmed the misregulation by Oct-25 variants via RNA *in situ* hybridisation (Figure S15B).

Interestingly, Sox2 expression was affected only in AC explants, but not in the double morphant embryos. This can be explained by considering two points: First, in animal caps levels and activities of the injected Oct-25 protein variants most likely exceed endogenous Oct-25 protein activity and regulate Sox2 expression in a dominant fashion. Secondly, formation of neural tissue in the embryo requires inductive influences including FGF signalling [39],and Sox2 transcription is stimulated by FGF8 [27], which is normally expressed in the mesoderm. Thus, the stimulating influence of FGF signalling on Sox2 transcription in the embryo may neutralize the repressive influence from deregulated Oct-25 expression, while the repressive activity of the deregulated Oct-25 levels prevails in animal caps in the absence of FGF signalling.

The remaining genes either failed to respond to one of the two Oct-25 protein variants (Zic2, Xiro1), or did not respond (Ngnr 1a, N-tubulin). These observations suggest an indirect effect. While it is possible that additional factors that are misregulated in xSuv4-20h morphants contribute to the neural phenotype, the combined results from ChIP experiments and Oct-25 variants define a pathway, in which xSuv4-20h enzyme dependent repression of Oct-25 is needed during gastrulation for proper neuroectoderm differentiation.

### Deregulated Oct-25 expression in xSuv4-20h double morphants inhibits neural differentiation

**Figure 7:**
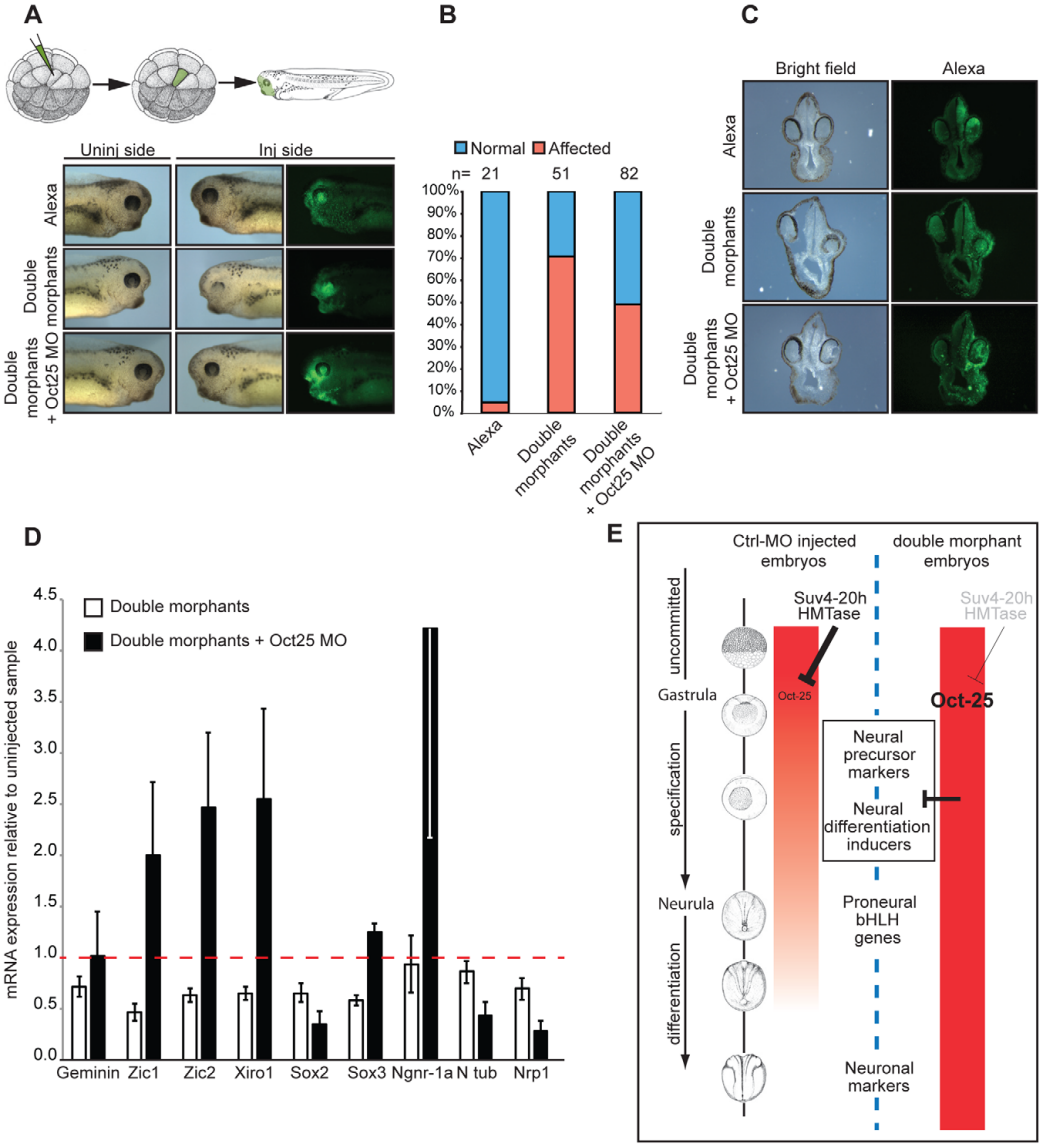
Oct-25 knockdown in double-morphant embryos rescues xSuv4-20h phenotypes. (A) Schematic illustration of targeting microinjections of tadpoles injected into the A1 blastomere at 32-cell stage, and morphological phenotypes of representative embryos (NF35-37) from cohorts injected with Alexa, xSuv4-20h MOs (double morphants) and double morphants plus Oct-25 MO. (B) Penetrance of the eye phenotype. Data from three independent experiments; n = total number of embryos scored. (C) Vibratome cross-sections of representative embryos injected as in panel (A). (D) qRT-PCR profiles for the indicated genes in double morphants and double morphants plus Oct-25 MO animal cap explants at NF 14–15. Data represent normalized mRNA levels as mean of three to four independent experiments; error bars indicate SEM. (E) Model for Xenopus Suv4-20h1/h2 enzyme function during neuroectoderm differentiation. A global increase in H4K20me3 reduces widespread Oct-25 expression in the animal emisphere during gastrulation as a prerequisite for neural induction. In H4K20me3 depleted morphant embryos, Oct-25 expression persists in the ectodermal stem cell compartment (sensorial cell layer), interfering with the transcriptional activation or activities of key regulators of the neural plate state and neurogenesis. Additional genes that are deregulated like Oct-25 in xSuv4-20h morphant embryos, may also contribute to impaired ectoderm differentiation.

To further analyse the mechanistic interaction between xSuv4-20h enzymes and Oct-25, we performed rescue experiments with triple-morphant embryos, in which synthesis of Oct-25 and xSuv4-20h proteins was simultanously blocked (Figure 7). The Oct-25 morpholino that we used has been shown before to inhibit efficiently Oct-25 translation from both non-allelic gene copies [40]. Because global Oct-25 depletion inhibits the formation of anterior neural structures [40], we employed two different strategies for the triple-knockdown to circumvent this problem. In a first series of analysis we injected a single A1 blastomere of 32-cell stage embryos to target cells that predominantly contribute to the retina and brain. Also in this experimental series, the morphology of double morphant eyes was strongly affected (Figure 7A). 71% of the injected embryos showed a clear reduction of retinal pigment, the remainders often restricted to the dorsal-most portion of the eyecup. The majority of the eyes contained no lens (Figure 7C). When the downregulation of xSuv4-20h enzymes was coupled to a concomitant knockdown of Oct-25 (triple morphants), the percentage of embryos showing this defect was reduced to 49% (p = 0.0188, Fisher's exact test). The retinal pigment was rescued in the triple morphants, whose eyes also regained a properly structured lens (Figure 7C). To confirm the morphological phenotypes, we investigated the basal neural gene expression in AC explants. The expression of a subset of genes involved in the establishment of the neural plate state (Zic1, Zic2, Xiro1, Sox2 and Sox3) was strongly reduced upon downregulation of xSuv4-20h enzymes at early neurula (NF14-15), compared to uninjected animal caps (p = 0.0068; p = 0.0127; p = 0.0113; p = 0.0321; p = 0.0037, respectively). With the exception of Sox2, the simultaneous downregulation of xSuv4-20h enzymes and Oct-25, rescued neural gene expression. In fact, under the triple morphant condition most of these genes were expressed at higher levels than normal, suggesting that they are partly repressed by Oct-25 in unmanipulated explants (Figure 7D). Most importantly, the combined results of the two triple-knockdown experiments indicate that both morphological and molecular features of the xSuv4-20h double morphant phenotype can be rescued to a significant extent by reducing Oct-25 protein levels. This result firmly establishes that the sustained and elevated expression of Oct-25 protein is responsible for the neural differentiation defect of xSuv4-20h double-morphant embryos.

### Murine Suv4-20h1/h2 double-knockout ES cells have elevated Oct4 levels in undifferentiating and differentiating conditions

**Figure 8:**
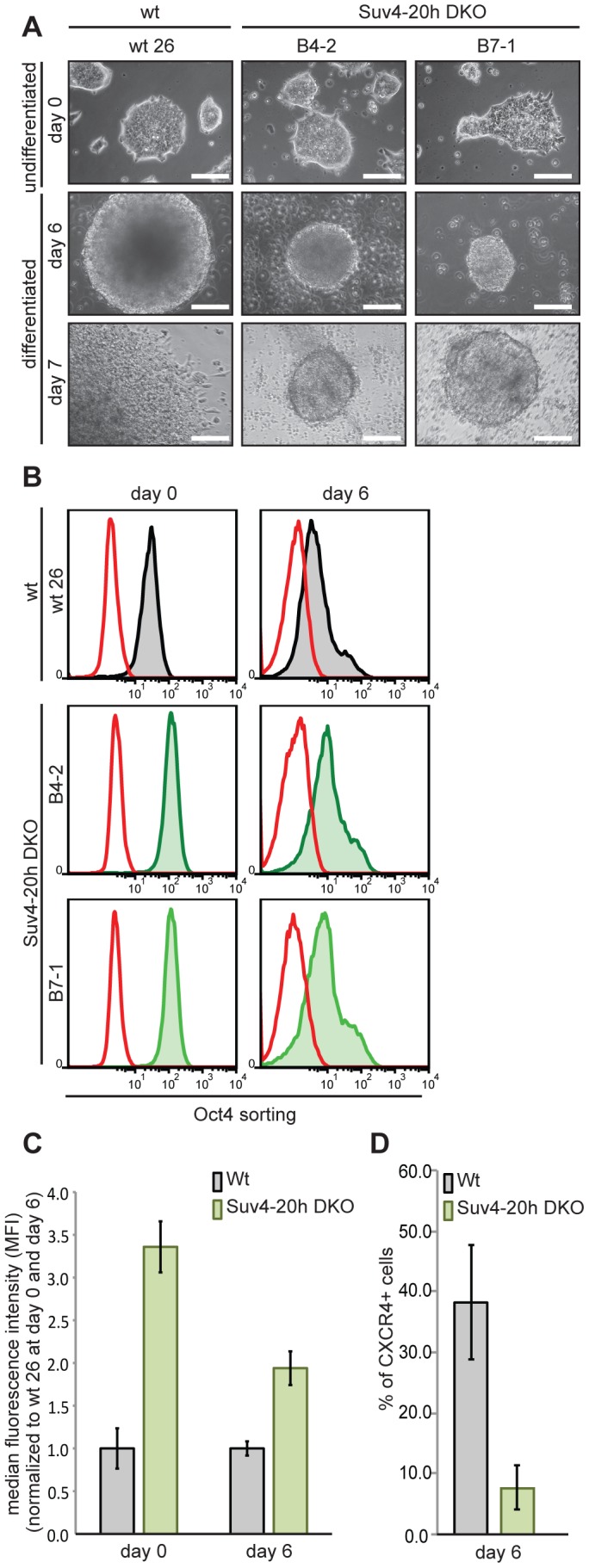
Suv4-20h double-null ES cells have elevated Oct4 and lower CXCR4 protein levels before and during differentiation. Wild-type and Suv4-20h DKO ES cells were grown undifferentiated in LIF-containing medium, or differentiated *in vitro* by embryoid body formation. Wt26 - isogenic wild-type ES cell line; B4-2, B7-1 – independent Suv4-20h DKO ES cell lines. (A) Morphological appearance (scale bar 100 µm). Top row: undifferentiated ES cells (day 0); middle: embryoid bodies at day 6; bottom: cells from embryoid bodies, replated for 24h. (B) Before (day 0) and during (day 6) differentiation, cell lines were stained for Oct4 protein and 2×10^4^ cells from each condition were subjected to FACS analysis. Red graph: fluorescence of non-specific isotype control; black and green graphs represent the Oct4 protein levels in wild-type and Suv4-20h DKO ES cell lines, respectively. (C) Suv4-20h DKO cells have higher Oct4 protein levels compared to wild-type ES cells and maintain these during differentiation. Median fluorescence intensity was calculated from data in panel (B), error bars indicate SEM. (D) Suv4-20h DKO cells show a reduction in the percentage of CXCR4 + cells at differentiation day 6. Data represent normalized values of percentage of CXCR4+ cells as means of three independent experiments, error bars indicate SEM.

Oct-25 plays multiple roles during early frog development, including interference with Activin/BMP-dependent mesendoderm formation before gastrulation, and with neural induction during gastrulation [32], [34]. A similar role is considered for its mammalian paralog Oct4, which is required for the pluripotent state of ES cells, but antagonizes ectodermal differentiation as soon as these cells exit pluripotency [30], [41], [42]. Although previous genome-wide studies of histone modifications in mammalian cells have not detected H4K20me3 on the Oct4 gene [36], [37], this apparent similarity made us investigate Oct4 protein expression in wild-type and Suv4-20h1/h2 DKO murine ES cells. We tested two independently derived DKO cell lines (B4-2 and B7-1), and compared them with two wild-type controls, i.e. wt26, an isogenic ES cell line, and the well-characterized GSES-1 cell line [43]. All four cell lines formed comparable ES cell colonies in LIF-containing medium (Figure 8A and Figure S17B), although the two DKO lines grew slightly slower. Upon aggregation they formed embryoid bodies, which were clearly smaller than those of the wild-type lines, both at day 2 and day 6 of differentiation (Figure 8A and Figure S17). After replating the differentiated cells for one day, the two DKO lines frequently formed again colonies resembling undifferentiated ES-cells (day 7 in Figure 8A and Figure S17B). To obtain a quantitative measure of Oct4 gene expression, we fixed and stained the four cell lines before (day 0) and during (day 6) differentiation for Oct4 protein and subjected equal cell numbers to FACS-analysis. The Oct4 signals were quite similar between wt26 and GSES-1 cells, as they were between the two DKO lines. In contrast to the wild-type cell lines, however, the signals of the DKO lines were reproducibly shifted to the right (Figure 8B and Figure S17C). Based on normalized median fluorescence intensity, the two DKO lines contained approximately three-fold higher Oct4 protein amounts than the wild-type lines at day 0 (p = 0.00604), and still two-fold more at day 6 (p = 0.01266) (n = 3; Figure 8C and Figure S17B). We conclude that Oct4 expression is being reduced during differentiation in Suv4-20h1/h2 DKO cells. However, these cells have higher Oct4 levels in the undifferentiated state, and maintain higher levels during differentiation in comparison to wild-type cells.

Oct4 protein levels are known to be tightly regulated [1] and to influence lineage decisions during ES cell differentiation [41], [42]. We therefore investigated the biological significance of the elevated Oct4 protein levels in Suv4-20h DKO ES cell lines. Unfortunately, the applied EB differentiation protocol promotes predominantly mesendodermal differentiation, which prevented the analysis of neural markers. Nevertheless, we performed FACS analysis on wt and Suv4-20h DKO cell lines stained for the chemokine receptor 4 (CXCR4) protein, whose expression indicates mesendoderm induction in embryoid bodies. At day 6 of differentiation, wt cell lines showed a robust increase in CXCR4 positive cells compared to day 0 (Figure 8D and data not shown). In contrast, both Suv4-20h DKO cell lines contained a significantly lower percentage of CXCR4 positive cells at day 6 when compared to the wild type cell lines (p = 0.03255; Figure 8). We also noted that replated wt cells frequently formed autonomously beating areas at differentiation day 14 (see Video S1), indicating functional cardiomyocyte formation, while contracting areas were never observed in the Suv4-20h DKO cells (Video S2; n = 4 experiments). Finally, qRT-PCR analysis indicated a reproducible and statistically significant shift in mesendoderm gene expression in the DKO ES cells, which show enhanced induction of FoxA2 (p = 0.00706) and reduced levels of Gata4 (p = 0.00037), compared to the wt ES cell lines (Figure S17D). Together, these results reveal a compromised and biased differentiation capacity for Suv4-20h DKO ES cell lines, and provide an entrypoint for further experimentation in the murine system.

## Discussion

In this study, we have investigated the developmental functions of the histone- methyltransferases Suv4-20h1 and h2 during frog embryogenesis, which are responsible for the establishment of the H4K20 di- and trimethylated states. These modifications have been implicated in heterochromatin formation, DNA damage repair and G1/S-transition [21], [24] and are also involved in transcriptional regulation [44], [45]. Our experiments identify a specific and selective role of xSuv4-20h HMTases in the formation of the ectodermal germlayer through control of mRNA expression of key regulators of the neural plate state and neuronal differentiation circuits. Indeed, our results indicate for the first time that H4K20me3 controls transcription in a rather gene-specific manner. The mRNA profile of double morphant embryos shows appr. 6% of the annotated probesets to be misregulated, when H4K20me3 levels have been reduced to appr. 25%. About half of the responding mRNAs are transcriptionally upregulated and, thus, their genes may qualify as being directly controlled by H4K20me3 deposition. Surprisingly, our molecular analysis revealed that xSuv4-20h enzymes are required to restrict the expression of the pluripotency-associated Oct-25 gene during gastrula and neurula stages. In the absence of proper H4K20me3 deposition, the Oct-25 gene becomes transcriptionally derepressed and interferes with neural differentiation. The successful rescue of key morphological and molecular aspects of the neural defect in double-morphant embryos by the simultanous inhibition of Oct-25 translation establishes this pathway formally. At least in Xenopus, the regulatory interaction between xSuv4-20h enzymes and Oct-25 is needed for embryonic cells to exit the pluripotent state and differentiate as neuroectoderm.

The genetic interaction between Suv4-20h enzymes and POU-V genes appears also to be conserved in mouse ES cells, although the H4K20me3 mark has not yet been detected on the Oct4 gene locus. To this point, we have shown that Suv4-20h DKO ES cells contain significantly elevated Oct4 protein levels, compared to wt ES cells. During ES cell differentiation the mammalian Oct4 gene is known to become repressed by a battery of epigenetic mechanisms including DNA methylation, incorporation of somatic linker histones and repressive histone modifications (H3K9me3/H3K27me3), which cooperate to achieve chromatin compaction of the Oct4 gene locus [46]. Our finding that Oct4 protein levels are increased in the DKO ES cells both before and during differentiation actually suggests that Suv4-20h enzymes regulate mammalian Oct4 transcription in a way that is at least partly independent from the other repressive mechanisms targetting this locus.

Our results in Xenopus rest predominantly on loss of function analysis, achieved by morpholino-mediated knockdown of endogenous xSuv4-20h protein translation. Specifically, we have shown that our antisense oligonucleotides block translation of xSuv4-20h1 and h2 isoforms *in vitro*, and significantly decrease H4K20me2 and –me3 levels *in vivo*, without altering the bulk abundance of other repressive histone marks such as H3K9me3 and H3K27me3. The morpholinos produced specific phenotypes, which were rescued on the morphological and molecular level by RNA-born co-expression of heterologous xSuv4-20h enzymes and, thus, originate from deficient H4K20me2/me3 states.

While xSuv4-20h double morphant embryos showed consistent phenotypes at high penetrance, we were surprised to see that H4K20me2 and –me3 states could be quantitatively increased in frog embryos without any obvious morphological or molecular consequences (Figures S8 and S9). This result can be explained considering first of all the higher stability of the knockdown by non-degradable morpholinos compared to the transient protein upregulation by RNA injection; secondly, demethylation of higher-methylated states may occur rather rapidly through H4K20me2 and me3 demethylases at specific sites, where H4K20me1 is required, e.g. Wnt/β-Catenin inducible genes [47]. However, we did not observe evidence for compromised transcription of Wnt target genes under overexpression (Figures S8 and S9) or morphant condition (Figure S5). Since mono- and dimethylated H4K20 states are quite abundant modifications in Xenopus embryos (30–40% each; see ref. [13]), it is most likely the loss of H4K20 trimethylation, which interferes with normal development.

XSuv4-20h double-morphant embryos were frequently defective for eye and melanocyte differentiation, indicating a prominent impairment of neuroectodermal differentiation. This selectivity is surprising, given that the two HMTases are expressed throughout the entire embryo (Figure S2). As a matter of fact, the phenotypes originate in the neuroectoderm, as shown by targeted injection into animal or vegetal blastomeres of 8-cell stage embryos (Figure 4). A large panel of marker genes that were investigated by RNA *in situ* hybridisation indicates that mesodermal and endodermal gene expression patterns are not perturbed by xSuv4-20h enzyme depletion (Figure 3A). This includes markers, which are required for specification of embryonic axes and formation and patterning of the mesendodermal germlayers (Figure S5). We also note that morphant animal cap explants were refractory to Noggin-dependent neural induction, but could be induced to differentiated skeletal muscle by a mesoderm inducing signal (Figure 5). We therefore assume that a major function of xSuv4-20h enzymes lies in the transcriptional control of genes that coordinate and execute neuroectodermal differentiation. Consistent with this hypothesis, many of the genes that we found downregulated in xSuv4-20h morphants, are key regulators of eye development (Rx-1, Pax-6), neuronal differentiation (Ngnr 1a, Delta-like 1) or regulators of neural competence and neural plate state (Zic-1, -2, -3, Xiro-1, Nrp1, N-CAM; [27]).

While these molecular results explain the overt morphological phenotypes in a consistent manner, it should be noted that these HMTases are clearly involved in additional cellular aspects. The mild reduction in mitotic cells and the increased apoptotic rate of morphant embryos (Figure S10) is reminiscent of findings in Suv4-20h1/h2 DKO MEFs, which are less resistant to DNA damage and compromised at the G1/S checkpoint [24]. The data reported here indicates a need for deeper analysis of the regulatory impact of Suv4-20h enzymes on transcription in both mammals and non-mammalian vertebrates.

According to current models, xSuv4-20h enzymes mediate transcriptional repression, based on the enrichment of the H4K20me3 mark on heterochromatic foci. Genes that are regulated by these enzymes should therefore become derepressed under loss of function condition. Following this logic, many of the genes, which are misregulated in morphant frog embryos, would be classified as indirect targets, since they were downregulated. One very notable exception, which we have validated as direct target, is Oct-25 (Figure 6). Oct-25 is induced broadly in the animal hemisphere at the blastula/gastrula transition, before it becomes restricted to the notoplate at neurula stages [31]. Oct-25 plays multiple roles during early frog development, including interference with Activin/BMP-dependent mesendoderm formation before gastrulation, and with neural induction during gastrulation [31], [32], [34]. Our study reveals now a new function for Oct-25, namely to control the transit from a pluripotent cell to a neural cell that differentiates, when Oct-25 expression has faded. As depicted in our model (Figure 7E), this function depends on the precise dose and duration of Oct-25 transcription, which is controlled by the level of H4K20me3 deposition on the first exon of the Oct-25 gene through xSuv4-20h enzymes. As we have shown here, deregulated transcription of Oct-25 in double-morphant embryos elicits massive consequences on the differentiation of neuroectodermal organs and cell types. We have traced back the origin of the malformations to the gastrula stage, when a gene network, defining the neural state, become perturbed by Oct-25. Some members of this network are good candidates for direct regulation through Oct-25 (e.g. Zic and Sox genes). However, since Oct-25 transcription persists ectopically at least until the mid-neural fold stage in the ectoderm, subsequent gene cascades involved in regional differentiation of the neuroectoderm could also be directly misregulated by Oct-25.

The specific and selective deregulation of Oct-25 transcription in a precise spatial domain, i.e. the sensorial cell layer of the ectoderm, implies a very intriguing role for xSuv4-20h enzymes. This domain contains not only the uncommitted precursors of neuronal and epidermal cell types, but – with regard to the involuting marginal zone – includes also mesodermal and endodermal precursor cells. The observed derepression of Oct-25 in this domain may thus reflect a conserved mechanism, by which Suv4-20h enzymes control pluripotency in the embryo. As discussed above, we have found Oct4 protein to be increased in two independent Suv4-20h double knockout ES cell lines under LIF-maintained self-renewal conditions, when compared to wt ES cells (Figure 8 and Figure S17). The DKO cell lines also maintain higher Oct4 levels during differentiation than wt ES cells, although their Oct4 levels get diminished in the course of 6 days. Recent data from several labs suggest that the pluripotency regulators Sox2 and Oct4 guide ES cells towards specific germ layer differentiation programs, when they exit the pluripotent state [41], [42]. Indeed, our findings are in agreement with Thomson and colleagues, who describe Oct4 to antagonize ectodermal specification and to direct mesendodermal cell fate decisions. The conserved Suv4-20h-dependent restriction of Oct4 expression may thus contribute to the germ-layer specification of embryonic cells, when they exit the pluripotent state.

## Materials and Methods

### Ethics statement

Animal work has been conducted in accordance with Deutsches Tierschutzgesetz; experimental use of Xenopus embryos has been licensed by the Government of Oberbayern (Projekt/AK ROB: 55.2.1.54-2532.6-3-11).

### Expression constructs and *in vitro* transcription

*Full length X. laevis* Suv4-20h1a (NM_001092308) and Suv4-20h2a (NM_001097050) cDNAs in pCMV-SPORT6 were provided by ImaGenes. Capped mRNAs were synthesized *in vitro* with SP6 RNA-Polymerase after HpaI linearization. Both cDNAs were subcloned via XhoI/EcoRI sites into pBluescript II KS to generate digoxygenin-labelled antisense probes with T3 RNA-Polymerase. Xenopus Bcl-2, Oct-25-VP16 and –EnR constructs were transcribed with SP6 RNA-Polymerase from NotI- (Bcl-2 and Oct-25-VP16) and SacII- (Oct-25-EnR) linearized pCS2+ plasmids, respectively. Mouse Suv4-20h1 and h2 enzymes were transcribed with SP6 from PvuI-linearized pCMVmyc-constructs [24]. Enzymatically inactive mouse Suv4-20h HMTases were generated via PCR-mutagenesis (see Text S1, Table S1 for primers). Synthetic mRNAs were injected in the animal pole of two-cell stage embryos at 2, 3 or 4ng per embryo. Rescue experiments with wt and mutated mRNAs were performed with 3ng of a 1∶1 mix of wt or mutated Suv4-20h1 and h2 mRNAs, injected into the animal pole of a single blastomere at two-cell stage. Xenopus Bcl-2 mRNA was injected unilaterally in the animal pole of two-cell stage embryos at 800 pg per embryo. Xenopus Oct-25-VP16, -EnR mRNAs were injected in the animal pole of two-cell stage embryos at 100 pg per embryo.

### Cell culture, microscopy, and FACS analysis

Mouse embryonic fibroblasts (MEF) wild type and Suv4-20h DKO cells [24] were cultivated in High Glucose DMEM with L-Glutamine and sodium pyruvate, complemented with 10% FCS, β-mercaptoethanol, non essential amino acids and penicillin/streptomycin in a 37°C incubator at 5% CO_2_. Lipofectamine 2000 (Invitrogen) was used for the transfection of plasmid DNAs. Immunofluorescence analysis was performed as described in the Text S1.

Mouse ES cells were cultivated on gelatinized plates in High Glucose DMEM with L-Glutamine and sodium pyruvate, complemented with 15% FCS, 0.1 mM ß-mercaptoethanol, non essential amino acids, penicillin/streptomycin and LIF. Cells were maintained at 37°C in a humidified atmosphere of 5% CO_2_. ES *in* vitro differentiation and FACS analysis were carried out as described [43] The incubation steps with the primary Oct4 (1∶250, Abcam) or CxCR4 (1∶50, BD Pharmingen) antibody and subsequently a FITC-conjugated secondary antibody (1∶250, Invitrogen) were performed at RT for 45 min with two washing steps after each antibody incubation. For the isotype controls purified, IgG was used instead of the Oct4-antibody. All FACS analyses were performed with an Epics XL (Beckman-Coulter) using the analysis software FlowJo.

### Morpholino oligonucleotides

Translation-blocking Morpholino oligonucleotides targeting Xenopus Suv4-20h1 (*X.laevis* and *X.*tropicalis: 5′-GGATTCGCCCAACCACTTCATGCCA-3′), Xenopus Suv4-20h2 (*X.laevis*: 5′-TTGCCGTCAACCGATTTGAACCCAT-3′: *X.tropicalis*: 5′-CCGTCAAGCGATTTGAACCCATAGT-3′) and Xenopus Oct-25 (*X.laevis*: 5′-TTGGGAAGGGCTGTTGGCTGTACAT-3′) mRNAs were supplied by Gene Tools LLC. Each Morpholinos recognizes the two non-allelic isoforms of each gene in *X.laevis* (see Figure S3A, S3B). GeneTools' standard control Morpholino was used to monitor non-specific effects. Morpholino activity was tested by *in vitro* translation (SP6-TNT Kit, Promega), adding 2 pg of control Morpholino or 1 pg of Suv4-20h1 and/or h2 Morpholinos per TNT reaction. Unless stated otherwise, embryos were injected at a dose of 60–80 ng per embryo (30–40 ng each of Suv4-20h1 and h2 Morpholinos, or 60–80 ng control Morpholino per embryo). For 8-cell stage experiments, morpholinos were injected in two neighbouring, animal or vegetal blastomeres on one side of the embryos, at half the dose (i.e. 40 ng total). For morphogical epistasis experiments, Xenopus Suv4-20h1 and h2 Morpholinos (5 ng each per embryo) and Oct-25 Morpholino (1 ng per embryo) were injected into A1 blastomere at 32-cell stage.

### Embryo handling

*Xenopus laevis* eggs were collected, fertilized *in vitro*, and handled following standard procedures; embryos were staged according to Nieuwkoop and Faber (1967). The embryos were injected with maximally 10 nl volume. When required, they were sorted into left side or right side injected cohorts before fixation, based on the coinjected lineage tracer Alexa Fluor-488 Dextran (Invitrogen). Alkaline-phosphatase stained and refixed embryos were either sectioned after embedding in paraffin (10 µm), or in gelatine/albumin mixture supplemented with 25% glutaraldehyde before sectioning (30–50 µm) with a Vibratome 1000 (Technical Products International, INC.) as described [48]. Animal caps were manually dissected at NF9 and transferred singly into wells of a 96-well plate, coated with 1% agarose and filled with 1X Steinberg's solution, 0.1% BSA with or without Activin A (1∶10 diluted conditioned cell culture supernatant). For neural induction, embryos were injected into the animal pole with Noggin mRNA (60 pg per embryo) alone or together with xSuv4-20h1 and h2 morpholinos (40 ng each per embryo) at two- to four-cell stage. For mesoderm induction, embryos were injected animally 4 times with 2.5 nl of control morpholino (80 ng per embryo) or a mix of xSuv4-20h1 and h2 morpholinos (40 ng each) at two or four cell stage. For Oct-25-VP16 and –EnR overexpression experiments, embryos were injected animally 4 times with 2.5 nl of each mRNAs (100 pg per embryo). For epistasis experiments on animal caps, embryos were injected 4 times with 2.5 nl of xSuv4-20h1 and h2 Morpholinos (40 ng each per embryo) and Oct-25 Morpholino (30 ng per embryo) at two or four cell stage.

### Analysis of histone modifications in Xenopus embryos

Nuclei extraction from Xenopus embryos and mass spectrometry analysis of histone modifications were performed as described [13]. Histone marks were quantitated as relative abundances of a specific modification state as a fraction of the amount of all modifications found for this peptide (for details see ref 13).

### RNA *in situ* hybridization and immunocytochemistry

Whole-mount RNA *in situ* hybridizations were performed as described (Sive et al. 2000). Embryos were photographed under bright light with a Leica M205FA stereomicroscope. The following antibodies were used for immunocytochemistry: H3S10P antibody (1∶300, Upstate Biotechnology), active Caspase3 antibody (1∶500, Promega), and myosin heavy chain antibody MF20 (1∶100 hybridoma cell culture supernatant), anti-mouse or anti-rabbit alkaline phosphatase-conjugated secondary antibodies (1∶1000, Chemicon).

### Western blots and immunostaining

Embryonic histones were purified via acidic extraction of nuclei as described [13], size-separated by SDS-PAGE and blotted onto PVDF membranes (Roth). Membranes were blocked with 3% BSA (Roth) in PBS and subsequently incubated o/n at 4°C with polyclonal rabbit antibodies against H4K20me1 (1∶6000), H4K20me2 (1∶1000), H4K20me3 (1∶500) [21], [24] and pan H3 (1∶25000, Abcam). Infrared (IR) 680 or 800 conjugated Goat anti Rabbit IgG (1∶5000, Li-Cor) were used as secondary antibodies (incubation o/n at 4°C). Signals were detected with an ODYSSEY Infrared Imaging System. To extract exogenous myc-tagged fusion proteins embryos were treated as described in the Text S1. Proteins were separated by SDS-PAGE, BSA-blocked PVDF membranes were incubated o/n at 4°C with anti-myc 9E10 antibody (1∶50), followed by anti-mouse HRP- conjugated antibody (1∶3000, Jackson Immunoresearch). Proteins were detected with ECL plus western blotting detection reagents (GE Healthcare). Histological sections were stained with pan H3 (1∶2000, Abcam), H4K20me1 (1∶5000), H4K20me2 (1∶2000), H4K20me3 (1∶5000) antibodies [24].

### Quantitative RNA analysis

Total cellular RNA was isolated with TRizol (Qiagen) and phenol/chloroform extraction. On-column RNA clean-up, including a DNAse digestion step, was performed using RNeasy-Mini-Kit (Qiagen). Samples for qRT-PCR and microarray profiling were collected as described in the Text S1.

### Microarray expression analysis

Microarray data were processed using R/Bioconductor (www.bioconductor.org). If not indicated otherwise, we used standard parameters in all functions calls. Expression values were calculated using ‘gcrma’. Probe sets were kept for differential expression analysis if there were more ‘present’ calls (calculated using ‘mas5calls’) in one of the treatment groups than non-‘present’ calls, if their expression level variance was higher than 0 across all arrays and if the probe set had an Entrez identifier annotation according to the Entrez database with a date stamp of 2011- Mar16. One gene to many probe set relationships were resolved by retaining only the probe set with the highest variance across all arrays. Differential expression statistics were obtained using a linear model (library ‘limma’). A significant response was defined if the local false discovery (‘locfdr’ package) rate calculated on the moderated t statistic was smaller than 0.2. The data discussed in this publication have been deposited in NCBI's Gene Expression Omnibus and are accessible through GEO Series accession number GSE41256 (http://www.ncbi.nlm.nih.gov/geo/query/acc.cgi?acc=GSE41256).

### ChIP experiments

ChIP experiments were performed using *Xenopus tropicalis* as described [49], with minor changes (see Text S1 for details).

### Identification of Oct-25 binding sites

A published weight matrix (PMID:17567999) was used to scan 2 kb upstream regions of selected *X. tropicalis* genes (Xenbase version 7.1) for binding site occurrence. Scanning was performed using RSA matrix-scan (PMID:18802439) with default parameters.

## Supporting Information

Figure S1 *Xenopus laevis* versus *Mus musculus* Suv4-20h proteins sequence alignment. Amino acid sequence alignment for *Mus musculus* (Refseq. NM_001167885.1) *versus Xenopus laevis* Suv4-20h1 (Refseq. NM_001092308) (A) and *Mus musculus* (Refseq. NM_146177.2) [24] *versus Xenopus laevis* Suv4-20h2 (Refseq NM_001097050) proteins (B). (C) Aminoacid sequence conservation (% identity) of the SET domain between mouse and Xenopus proteins.
(PDF)

Figure S2 *Xenopus laevis* Suv4-20h expression during early development. XSuv4-20h1 (A) and xSuv4-20h2 (B) mRNA expression was detected by RNA *in situ* hybridization at the indicated developmental stages. (C) Total RNA was extracted from animal cap (AC), marginal zone (MZ) and vegetal pole (VP) explants of NF9 embryos; semiquantitative PCR shows relative levels of xSuv4-20h1 and xSuv4-20h2 transcripts in the three explants. ODC was used as loading control, -RT as negative control. (D) qRT-PCR profiles of xSuv4-20h enzymes. The chart shows the expression of the two enzymes relative to ODC at the indicated developmental stages.
(PDF)

Figure S3 Morpholino specificity. 25-mer xSuv4-20h1 morpholino (A) and xSuv4-20h2 morpholino (B) oligonucleotides perfectly target to the start codon of the respective two non-allelic isoforms. Sequence differences between the two morpholinos confer specificity of each oligonucleotide for either xSuv4-20h1 or xSuv4-20h2 mRNA. (C) *In vitro* TNT assay performed as described in Materials and Method section. xSuv4-20h1 and h2 MOs specifically inhibited translation of their cognate templates.
(PDF)

Figure S4 Quantification of histone methylation states in xSuv4-20h morphants by MALDI-TOF mass spectrometry. Bulk histones from NF30-33 embryos were isolated and analysed as described in Materials and Methods. (A) H4 peptide 20–23, (B) H3 peptide 9–17 and (C) H3 peptide 27–40 in uninjected, ctrl-MO and double morphant embryos. The values represent mean of three independent experiments; error bars indicate SEM. Star - for technical reasons H4K20me3 mark was quantitated only in some samples (star). (D) Western Blot analysis of uninjected, control morpholino (ctrl-MO) and xSuv4-20h1, h2 morpholino injected embryos using antibodies against H3K27me3 and H3K9me3. PanH3 antibody was used as loading control.
(PDF)

Figure S5 Germ layer marker gene expression in xSuv4-20h double morphants. (A) Immuno-histochemistry on ctrl-MO and xSuv4-20h double-morphant tadpoles. Panels show representative cross-sections of neural tubes stained with antibodies against the histone epitopes indicated on top. Inj – injected side. Squares on double-morphant sections represent the croped pictures shown in Figure 1D. (B-F) RNA *in situ* hybridization analysis of ctrl-MO injected and double morphant embryos at the indicated stages using probes against Chordin, Xnr-3 and Gooscoid (B), Krox20 and Otx2 (C), Pax-6 (D), N-CAM (E), FoxD5, Geminin, Zic2, Zic3, Sox3, Sox11 and VegT (F). Chordin, Xnr-3 and Gooscoid – dorsal side views; animal pole is on the top. Krox20 - dorsal views, anterior on the left. Otx2 - anterior views, dorsal on the top. Pax-6 – head region; rescued embryos included. N-CAM - dorsal views of stained embryos with the anterior on the left; rescued embryos included. FoxD5, Geminin, Zic2, Zic3 - dorsal views; injected halves are lineage-traced by coinjection of LacZ mRNA and subsequent β–Gal staining (light blue). Sox2, Sox3 - dorsal views, anterior to the left. VegT - internal stain in bisected embryos.
(PDF)

Figure S6 Functional SET domains are required for proper Suv4-20h activity. (A) Schematic of *Mus musculus* Suv4-20h1 and h2 SET domain mutations. (B) Immunofluorescence of wild-type and Suv4-20h DKO MEFs transfected with the indicated constructs. (C) Western Blot analysis of NF11.5 uninjected embryos (lane 1), xSuv4-20h1, h2 double morphants (lane 2), active and inactive mouse Suv4-20h1, h2 mRNAs injected embryos (lane 3, 5 respectively) and double morphant embryos cojnected with active or inactive mSuv4-20h1, h2 mRNAs (lane 2, 4 respectively), using antibodies against H4K20 mono-, di- and trimethylation. PanH3 antibody was used as loading control. (D) Anti-myc western blot with the same samples used in B. Asterisks indicate unspecific bands.
(PDF)

Figure S7 A functional SET domain is required for morphological and molecular rescue of double-morphants phenotypes. (A–C) Morphological phenotypes of NF30-33 double morphants (A), embryos injected with xSuv4-20h1, h2 morpholinos and active (active rescue, B), or inactive (inactive rescue, C) mouse Suv4-20h1, h2 mRNAs. Embryos were coinjected in one half at two cell stage with Alexa Fluor 488 Dextran as lineage tracer (green channel) to identify the injected side and sort embryos. (D) Penetrance of the eye phenotype in the indicated samples. Displayed are the results from two independent experiments. (E) RNA *In situ* hybridization analysis of NF15 uninjected, double morphants, active and inactive rescue embryos using probes against Oct-25, N-tubulin and MyoD. The pictures show dorsal view of stained embryos, anterior is on the left.
(PDF)

Figure S8 *Xenopus laevis* Suv4-20h1 or h2 mRNA overexpression. (A) Overexpression of frog Suv4-20h1 and h2 enzymes causes an upregulation of H4K20me2 and H4K20me3 marks. Bulk histones from uninjected embryos or embryos bilaterally injected with increasing amounts of Suv4-20h1 or h2 mRNAs were isolated at NF11.5 and analysed by Western blot. Pan H3 antibody was used as loading control. (B, C) Morphological phenotypes of NF30-33 embryos injected with xSuv4-20h1 (B) or h2 (C) mRNA. (D) RNA *In situ* hybridization of NF30-33 uninjected embryos (top row) and embryos injected with Suv4-20h1 (middle row) or h2 (bottom row) mRNA using probes against Rx-1 and Pax-6. Pictures show the head of stained embryos. (E) RNA *In situ* hybridization analysis of uninjected embryos (top row) and embryos injected with xSuv4-20h1 (middle row) or h2 (bottom row) mRNA using probes against Ngnr-1a, Delta-like 1, N-tubulin, Xbra, MyoD, Sox17 α and Endodermin. Pictures show dorsal views of stained embryos, anterior is on the left; Xbra pictures show vegetal views of NF11 embryos; MyoD pictures show dorsal views of NF15 embryos, with the head on the left. For Sox-17 α and Endodermin sagittal sections of NF15 embryos were created; pictures show internal view of the injected halves, with anterior on the left.
(PDF)

Figure S9 *Mus musculus* Suv4-20h1 or h2 mRNA overexpression. (A) Western Blot analysis of uninjected embryos or embryos injected with *Mus musculus* Suv4-20h1 or h2 mRNAs at different concentrations. Bulk histones from NF11.5 embryos were isolated and analyzed as described in Materials and Methods section. Pan H3 antibody was used as loading control. (B, C) Morphological phenotypes of NF30-33 embryos injected with mouse Suv4-20h1 (B) or h2 (C) mRNA. (D) RNA *In situ* hybridization analysis of uninjected embryos (top row) and embryos injected with mSuv4-20h1 (middle row) or h2 (bottom row) mRNA using probes against Ngnr 1a, Delta-like 1 and Rx-1. Rx-1 pictures show the head of NF30-33 stained embryos. Ngnr 1a (NF12.5) and Delta-like 1 (NF13) pictures show dorsal view of stained embryos, anterior is on the left.
(PDF)

Figure S10 Cell proliferation and apoptosis in xSuv4-20h double morphants. (A) Double morphants show increased number of apoptotic cells during neurulation. Top row – immunocytochemistry for active Caspase3 in unilaterally injected embryos (NF15). Middle and bottom rows - RNA *in situ* hybridisation for Delta-like 1 (NF13) and N-tubulin mRNAs (NF15). Pictures show dorsal views, with anterior to the left. (B) Proliferation assay – immunocytochemistry for the mitotic histone modification H3S10P in Crtl-MO *versus* double morphant embryos. The chart shows a two-fold difference in the number of H3S10P positive cells on the injected side of double morphants. Data represent mean values of four embryos per condition from two independent experiments; error bars indicate SEM.
(PDF)

Figure S11 qRT-PCR analysis. (A) Schematic representation of mRNA purification from NF14-15 embryos for qRT-PCR experiments. (B) qRT-PCR profiles for the indicated genes in Ctrl-MO injected and xSuv4-20h double morphant embryos. Data represent normalized ratios of mRNA levels as means of four independent experiments, error bars indicate SEM.
(PDF)

Figure S12 Microarray analysis. (A) Schematic representing mRNA purification from NF 14–15 embryos for microarray experiments. (B) Pie-chart showing number of up- (green) and down- (red) regulated genes. (C) Histogram summarizing the fold expression change of the analysed 9752 active genes. Indicated in red are responder genes (153up, 169 down). (D) Table presenting the 10 most upregulated genes. For each gene, the gene name, symbol, the log fold change (logFC) and the fold change are indicated.
(PDF)

Figure S13 Oct-25 and N-tubulin gene expression in X. tropicalis Suv4-20h double morphant embryos. RNA i*n situ* hybridization analysis of ctrl-MO injected and double morphant embryos at neurula stage (NF14-15) using probes against Oct-25 and N-tubulin. The pictures show dorsal views of the open neural plate with anterior to the left. Dashed line: embryonic midline.
(PDF)

Figure S14 Genes analysed by ChIP. (A) Schematic representation of the genes and the amplicons analysed in ChIP experiments. Black boxes: exons; white boxes: untranslated regions; line connecting boxes: introns. For each gene the position of the amplicon(s) used in the experiments is indicated below the gene structure. (B) qRT-PCR profiles for GAPDH and thra genes in Ctrl-MO injected and xSuv4-20h double-morphant embryos. Data represent normalized ratios of mRNA levels as means of five independent experiments, error bars indicate SEM.
(PDF)

Figure S15 Regulation of early neural marker genes by Oct-25-VP16 and Oct-25-EnR fusion proteins. (A) qRT-PCR on animal cap (AC) explants cut from uninjected embryos and embryos overexpressing Oct-25-VP16 or Oct-25-EnR mRNAs. The chart shows the relative expression of the indicated genes compared to H4 gene levels. Data represent normalized ratios of mRNA levels as means of three or four independent experiments, error bars indicate SEM. (B) *In situ* hybridization on uninjected AC or explants overexpressing Oct-25-VP16/EnR for Zic1 (upper row, 20× magnification) and Zic3 genes (lower row, 50× magnification).
(PDF)

Figure S16 Oct-25 binding sites on Zic1, Zic3 and Sox2 genes. Oct-25 hypothetical binding sites on Zic1, Zic3 and Sox2 have been identified as described in the Material and Methods section. The schematic representation of the genes shows: black boxes: exons; white boxes: untranslated regions; line connecting boxes: introns. For each gene the position of the binding sites, the identified sequence and the similarity to the published weight matrix (weight) are indicated in the underneath table. For Zic3 the six highest identified sequences are shown.
(PDF)

Figure S17 ES cell analysis. (A) Morphological appearance of differentiated day 2 embryoid bodies: B4-2 and B7-1 cell lines formed smaller bodies than those of the wild-type line wt26. (B) GSES-1 morphology and *in vitro* differentiation at the indicated days. Scale bar: 100 µm. (C) FACS profiles of GSES-1 cell line before (day 0) and during (day 6) differentiation. Red graphs: fluorescence of non-specific isotype control; black graphs: Oct4 protein levels in GSES-1 cells; green dashed graphs: Oct4 protein levels in mutant B7-1 cell line. (D) qRT-PCR profiles for the indicated genes in wild-type (wt) and Suv4-20h DKO cell lines at differentiation day 6. FoxA2 and Gata4 expression levels are misregulated in Suv4-20h DKO cells upon differentiation.
(PDF)

Table S1 List of oligonucleotide sequences used in this study.
(PDF)

Text S1 Supporting information on experimental procedures. This file contains additional information on statistical analysis, extraction of Myc-tagged fusion protein from embryos, qRT-PCR samples preparation, Vibratome sections of Oct-25 stained embryos, Immunostaining, Immunofluorescence microscopy of MEF cells and ChIP (chromatin immunoprecipitation) analysis.
(DOC)

Video S1 Videorecording of wild-type ES cells at day 14 of differentiation. The video shows several areals of autonomously beating cardiomyocytes.
(M4V)

Video S2 Videorecording of Suv4-20h DN ES cells at day 14 of differentiation. In contrast to differentiated wild-type ES cells, spontanous contractions of cardiomyocytes are not observed.
(M4V)
