# Supplementary figures and images for: Suv4-20h Histone Methyltransferases Promote Neuroectodermal Differentiation by Silencing the Pluripotency-Associated Oct-25 Gene
Source: PLoS Genet. 2013 Jan 31;9(1):e1003188. doi: 10.1371/journal.pgen.1003188 (PMC3561085; doi:10.1371/journal.pgen.1003188)

A

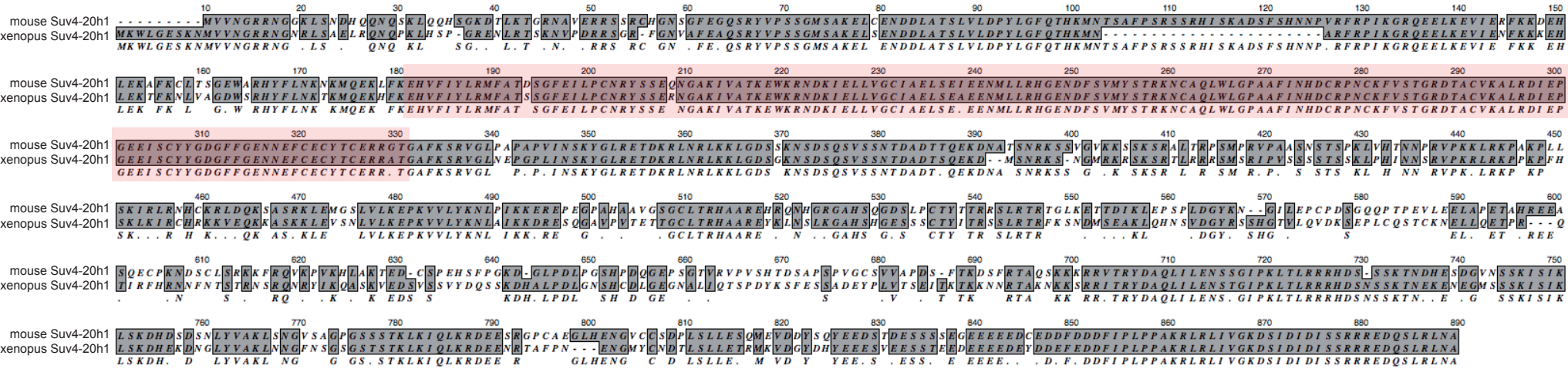

B

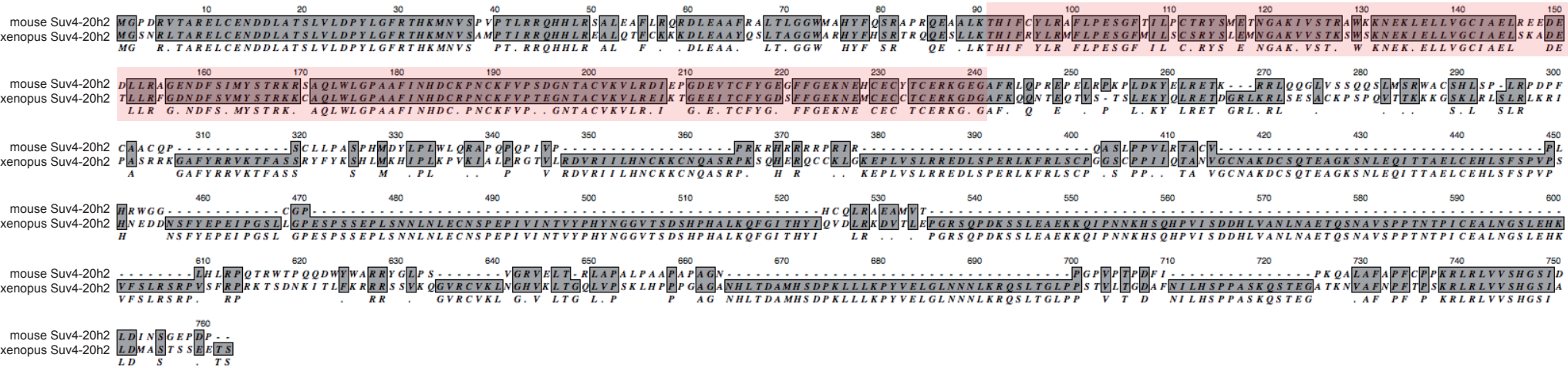

C

|            | xSuv4-20h1 | xSuv4-20h2 | mSuv4-20h1 | mSuv4-20h2 |
|------------|------------|------------|------------|------------|
| xSuv4-20h1 | -          | 83%        | 98%        | 81%        |
| xSuv4-20h2 | 83%        | -          | 83%        | 88%        |
| mSuv4-20h1 | 98%        | 83%        | -          | 75%        |

Supplement: Figure S1 — Xenopus laevis versus Mus musculus Suv4-20h proteins sequence alignment. Amino acid sequence alignment for Mus musculus (Refseq. NM_001167885.1) versus Xenopus laevis Suv4-20h1 (Refseq. NM_001092308) (A) and Mus musculus (Refseq. NM_146177.2) [24] versus Xenopus laevis Suv4-20h2 (Refseq NM_001097050) proteins (B). (C) Aminoacid sequence conservation (% identity) of the SET domain between mouse and Xenopus proteins. (PDF) [file pgen.1003188.s001.pdf]

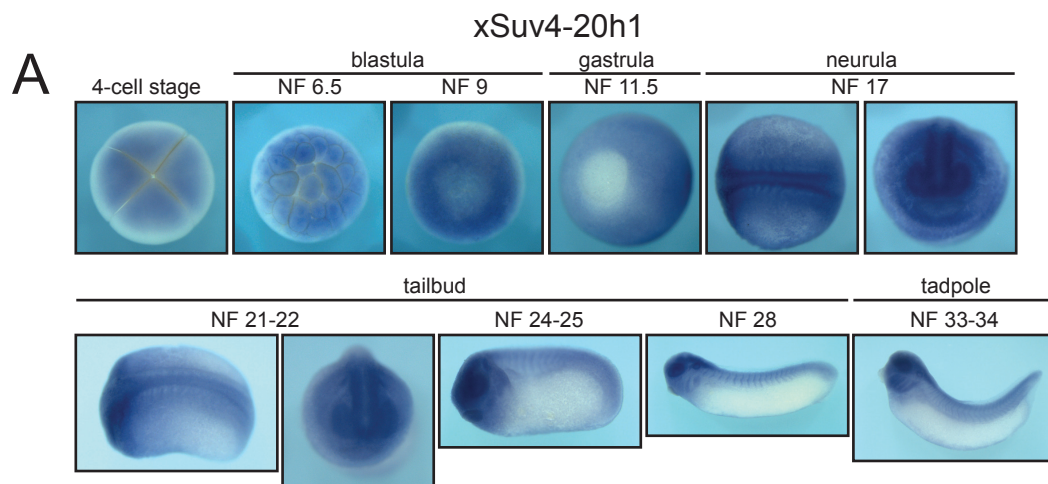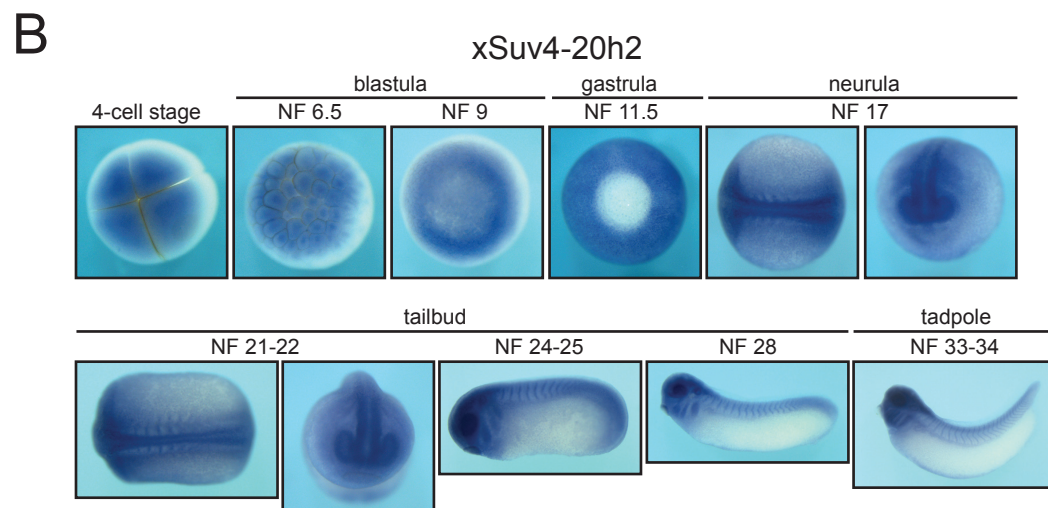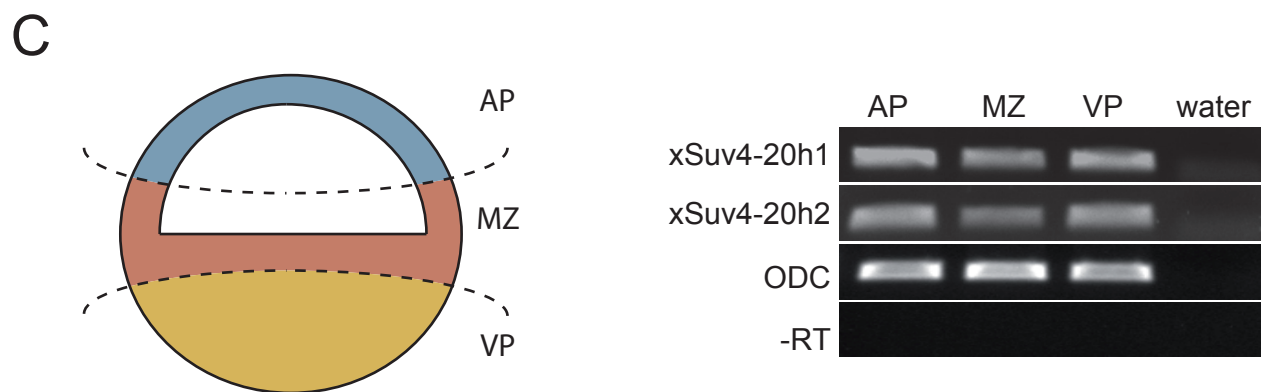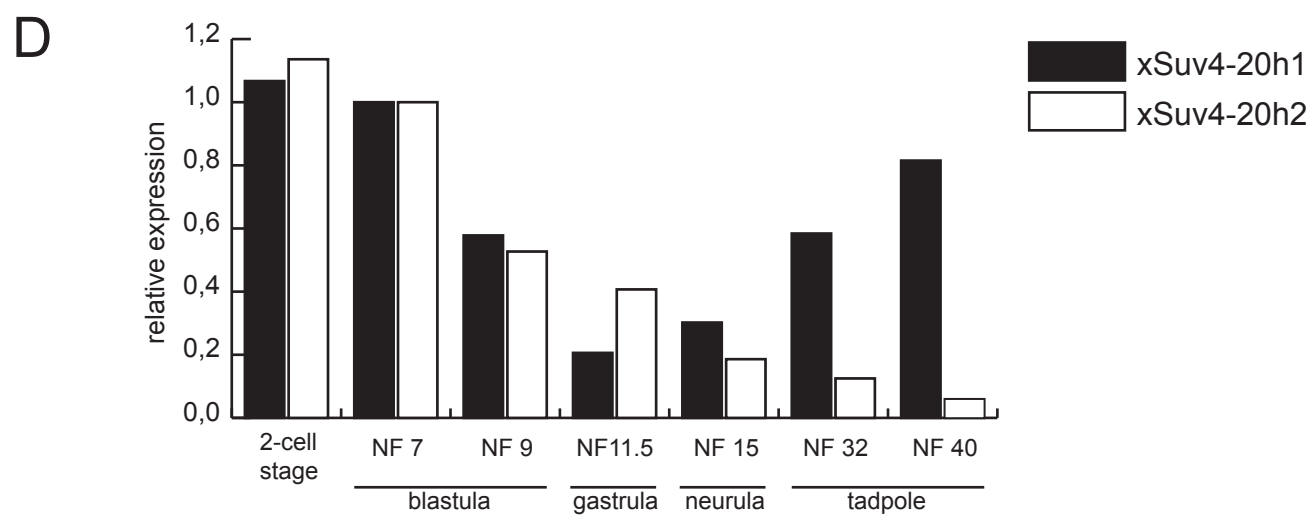

Supplement: Figure S2 — Xenopus laevis Suv4-20h expression during early development. XSuv4-20h1 (A) and xSuv4-20h2 (B) mRNA expression was detected by RNA in situ hybridization at the indicated developmental stages. (C) Total RNA was extracted from animal cap (AC), marginal zone (MZ) and vegetal pole (VP) explants of NF9 embryos; semiquantitative PCR shows relative levels of xSuv4-20h1 and xSuv4-20h2 transcripts in the three explants. ODC was used as loading control, -RT as negative control. (D) qRT-PCR profiles of xSuv4-20h enzymes. The chart shows the expression of the two enzymes relative to ODC at the indicated developmental stages. (PDF) [file pgen.1003188.s002.pdf]

A

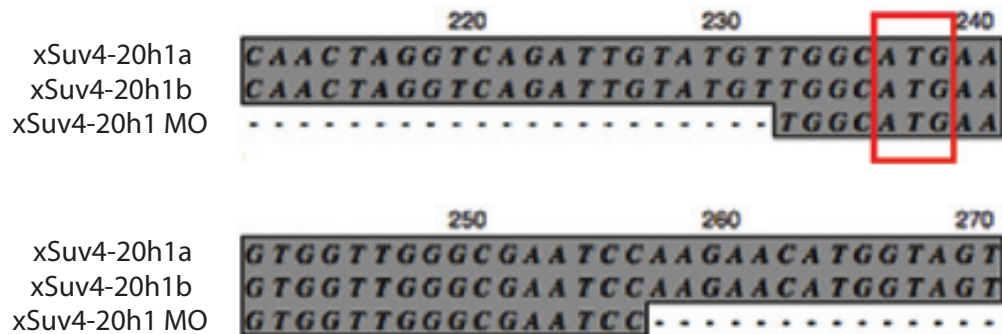

B

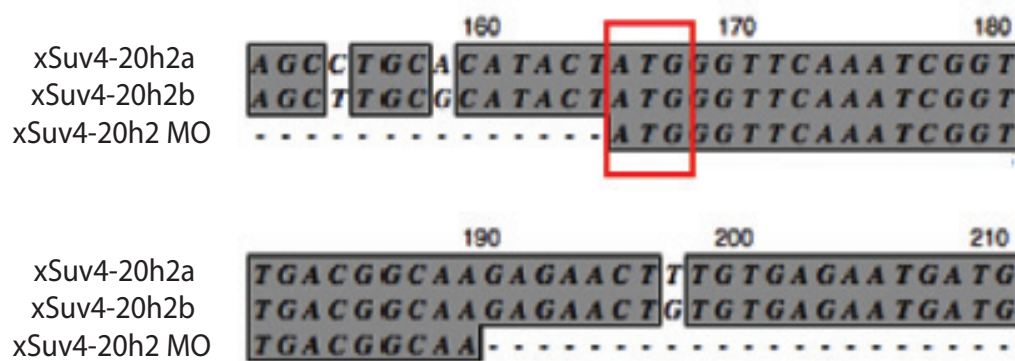

C

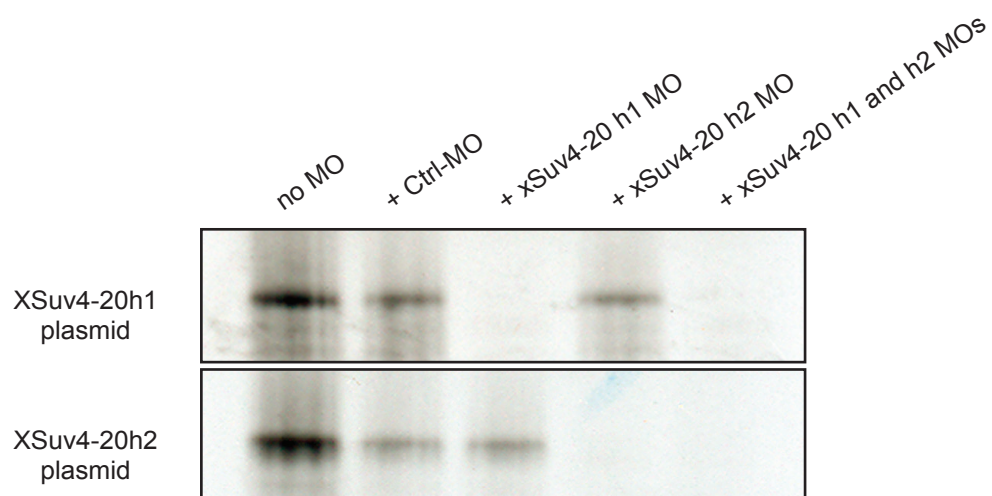

Supplement: Figure S3 — Morpholino specificity. 25-mer xSuv4-20h1 morpholino (A) and xSuv4-20h2 morpholino (B) oligonucleotides perfectly target to the start codon of the respective two non-allelic isoforms. Sequence differences between the two morpholinos confer specificity of each oligonucleotide for either xSuv4-20h1 or xSuv4-20h2 mRNA. (C) In vitro TNT assay performed as described in Materials and Method section. xSuv4-20h1 and h2 MOs specifically inhibited translation of their cognate templates. (PDF) [file pgen.1003188.s003.pdf]

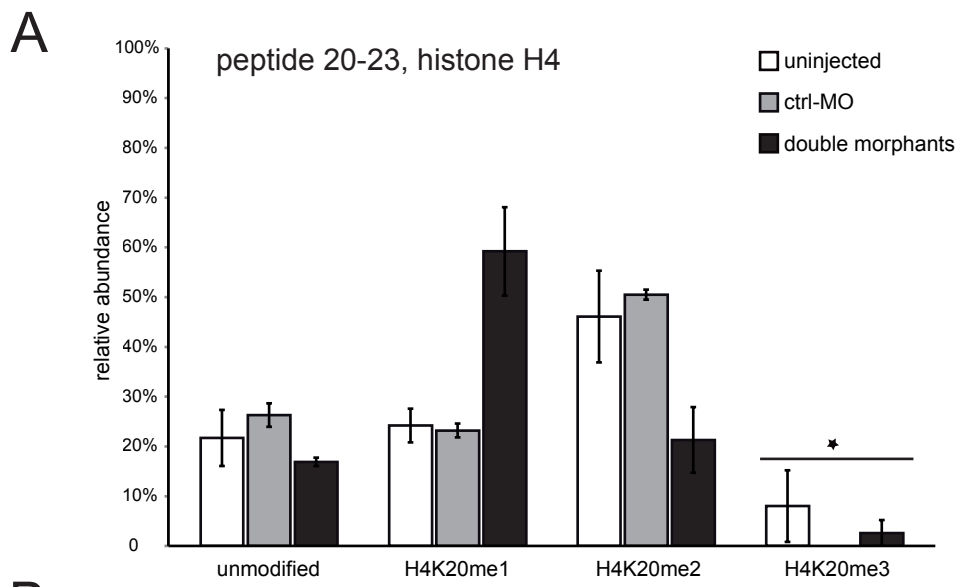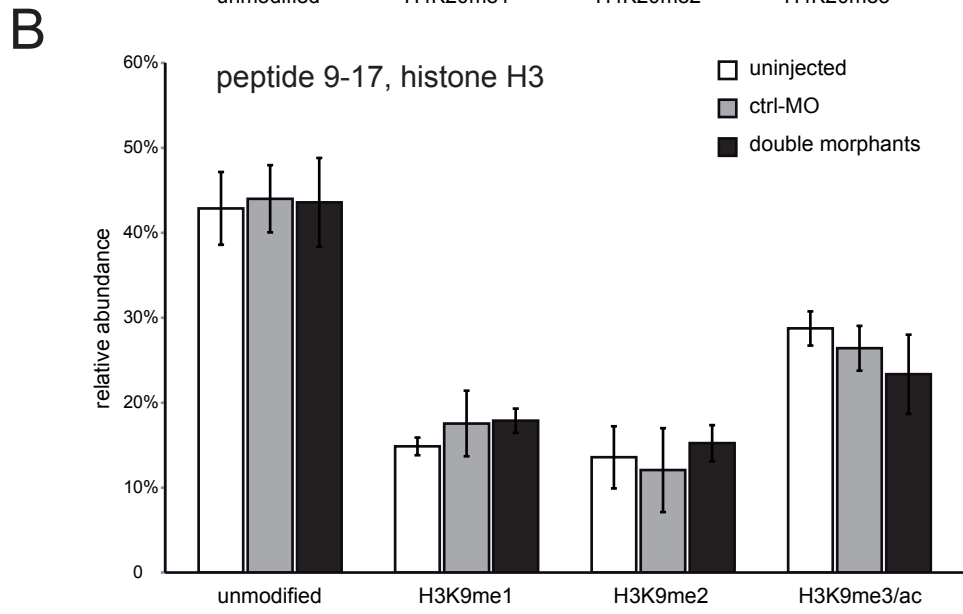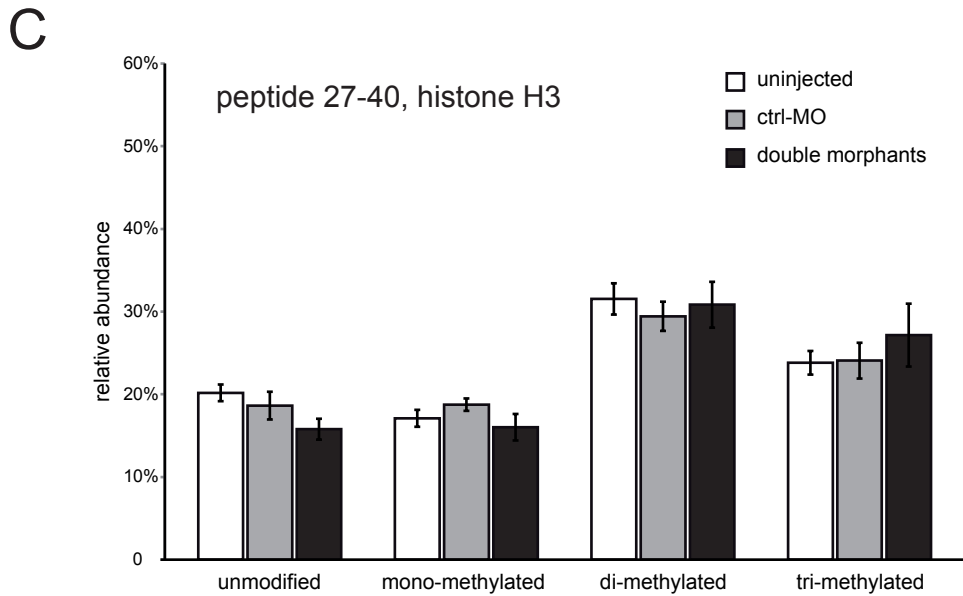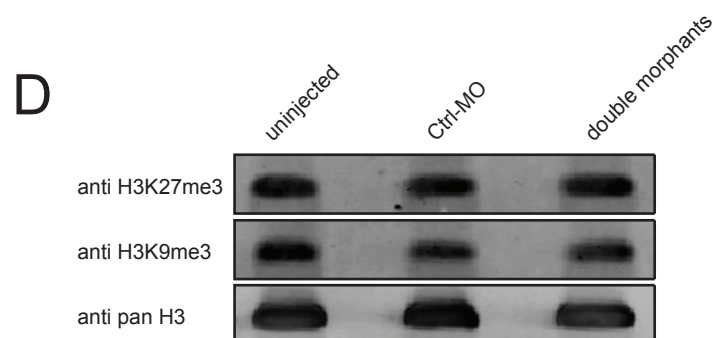

Supplement: Figure S4 — Quantification of histone methylation states in xSuv4-20h morphants by MALDI-TOF mass spectrometry. Bulk histones from NF30-33 embryos were isolated and analysed as described in Materials and Methods. (A) H4 peptide 20–23, (B) H3 peptide 9–17 and (C) H3 peptide 27–40 in uninjected, ctrl-MO and double morphant embryos. The values represent mean of three independent experiments; error bars indicate SEM. Star - for technical reasons H4K20me3 mark was quantitated only in some samples (star). (D) Western Blot analysis of uninjected, control morpholino (ctrl-MO) and xSuv4-20h1, h2 morpholino injected embryos using antibodies against H3K27me3 and H3K9me3. PanH3 antibody was used as loading control. (PDF) [file pgen.1003188.s004.pdf]

**A**

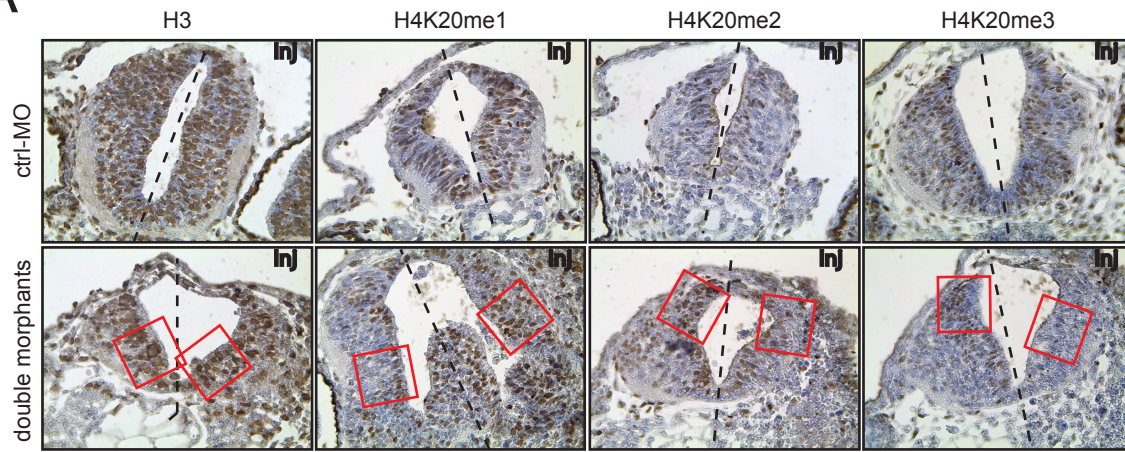

**B**

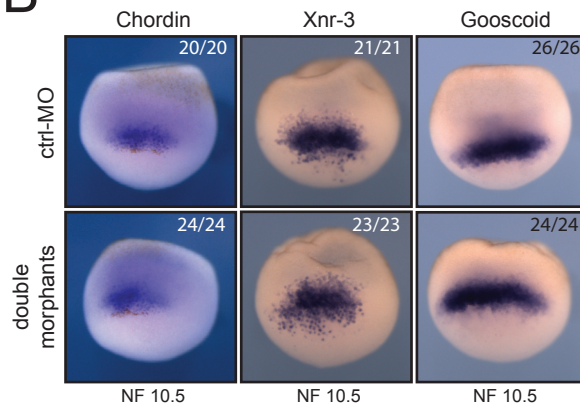

**C**

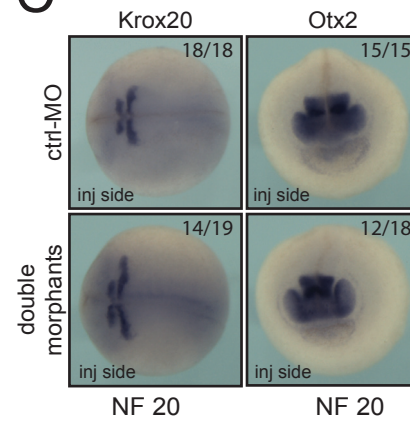

**D**

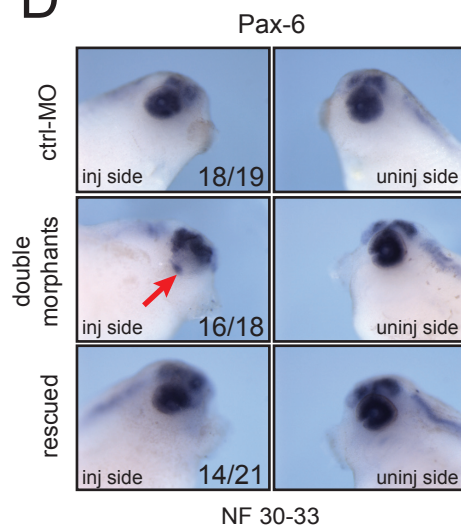

**E**

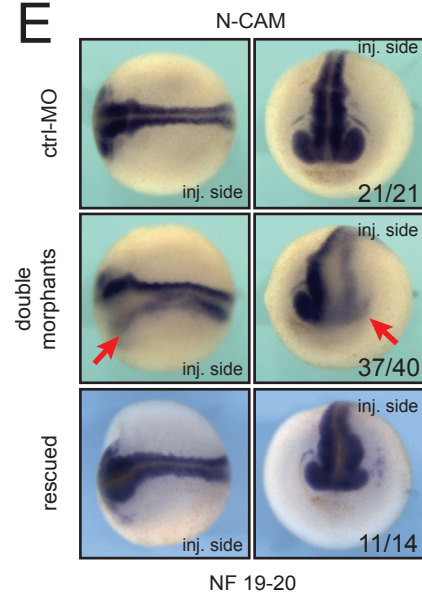

**F**

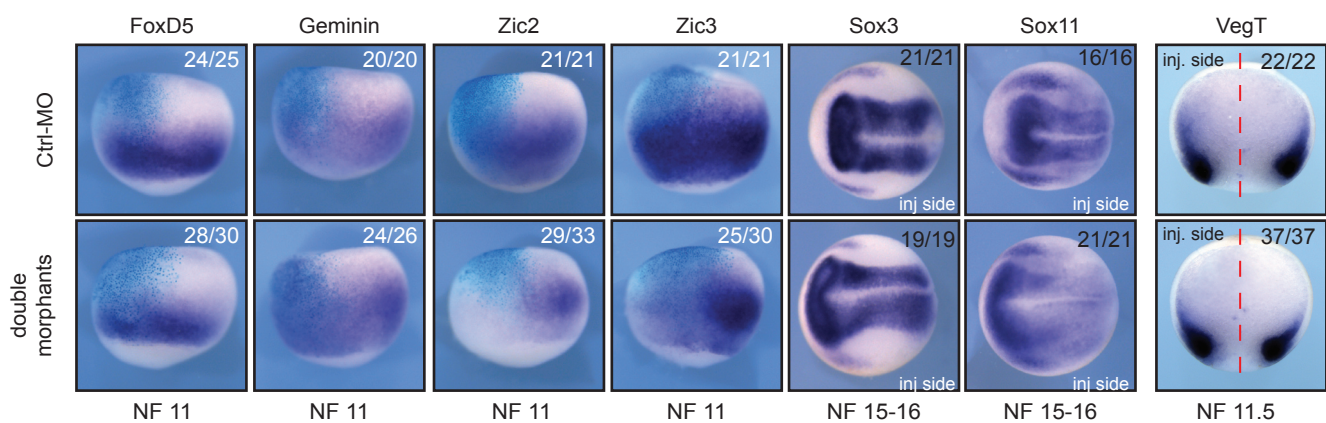

Supplement: Figure S5 — Germ layer marker gene expression in xSuv4-20h double morphants. (A) Immuno-histochemistry on ctrl-MO and xSuv4-20h double-morphant tadpoles. Panels show representative cross-sections of neural tubes stained with antibodies against the histone epitopes indicated on top. Inj – injected side. Squares on double-morphant sections represent the croped pictures shown in Figure 1D. (B-F) RNA in situ hybridization analysis of ctrl-MO injected and double morphant embryos at the indicated stages using probes against Chordin, Xnr-3 and Gooscoid (B), Krox20 and Otx2 (C), Pax-6 (D), N-CAM (E), FoxD5, Geminin, Zic2, Zic3, Sox3, Sox11 and VegT (F). Chordin, Xnr-3 and Gooscoid – dorsal side views; animal pole is on the top. Krox20 - dorsal views, anterior on the left. Otx2 - anterior views, dorsal on the top. Pax-6 – head region; rescued embryos included. N-CAM - dorsal views of stained embryos with the anterior on the left; rescued embryos included. FoxD5, Geminin, Zic2, Zic3 - dorsal views; injected halves are lineage-traced by coinjection of LacZ mRNA and subsequent β–Gal staining (light blue). Sox2, Sox3 - dorsal views, anterior to the left. VegT - internal stain in bisected embryos. (PDF) [file pgen.1003188.s005.pdf]

**A**

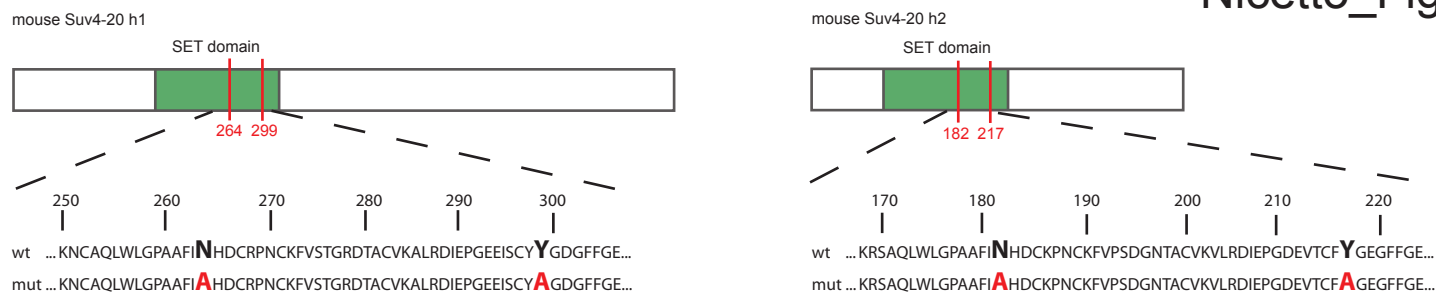

**B**

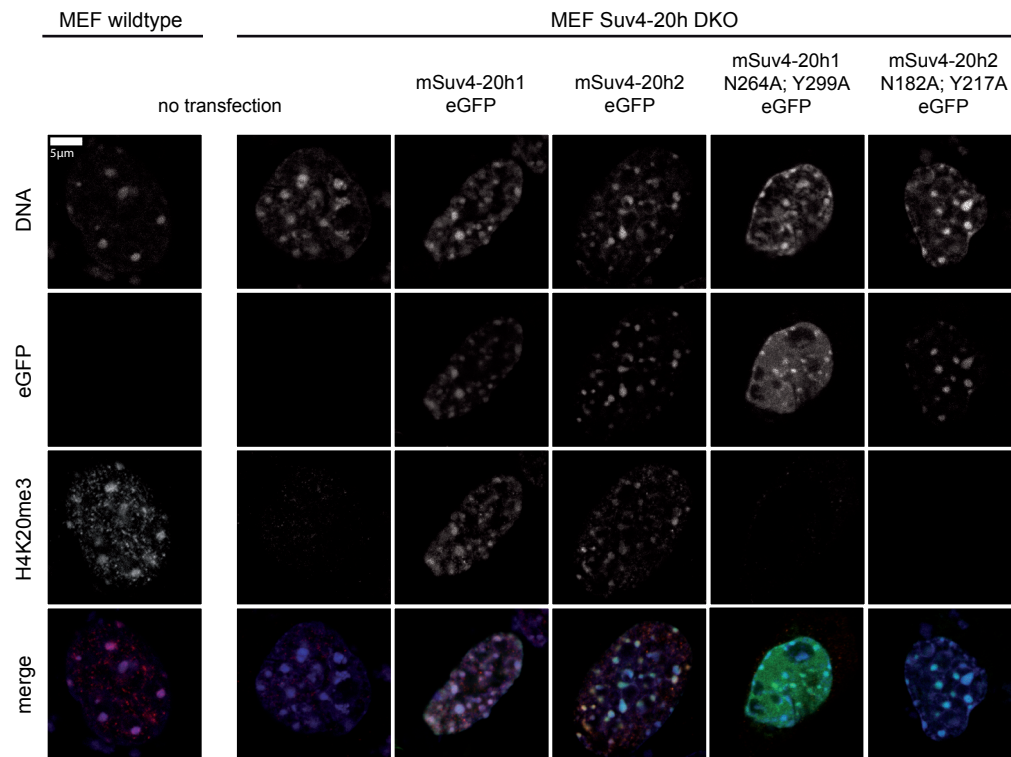

**C**

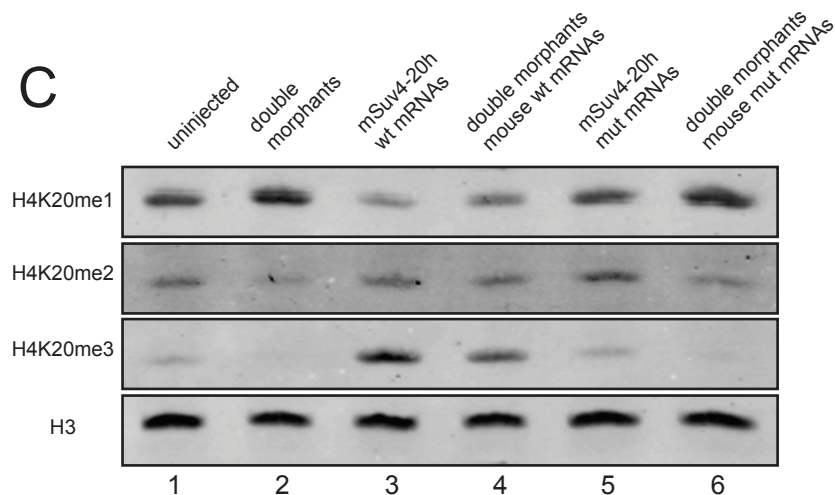

**D**

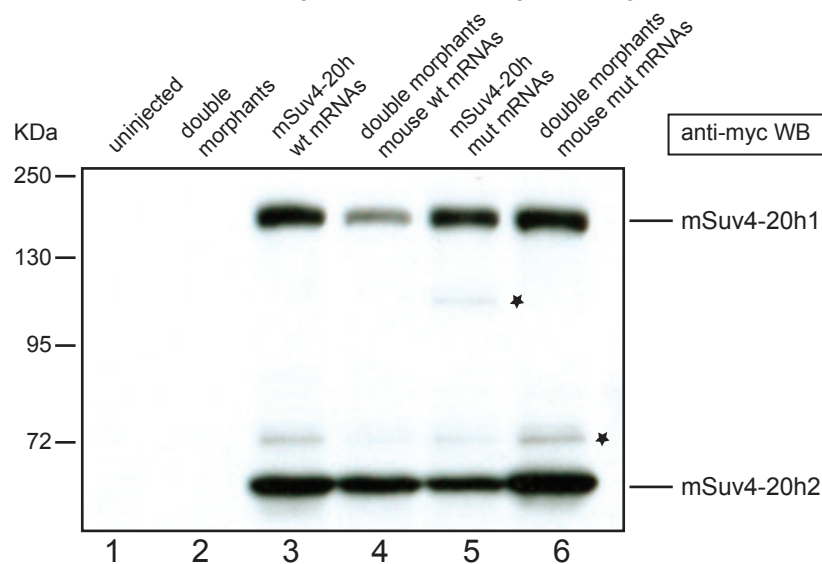

Supplement: Figure S6 — Functional SET domains are required for proper Suv4-20h activity. (A) Schematic of Mus musculus Suv4-20h1 and h2 SET domain mutations. (B) Immunofluorescence of wild-type and Suv4-20h DKO MEFs transfected with the indicated constructs. (C) Western Blot analysis of NF11.5 uninjected embryos (lane 1), xSuv4-20h1, h2 double morphants (lane 2), active and inactive mouse Suv4-20h1, h2 mRNAs injected embryos (lane 3, 5 respectively) and double morphant embryos cojnected with active or inactive mSuv4-20h1, h2 mRNAs (lane 2, 4 respectively), using antibodies against H4K20 mono-, di- and trimethylation. PanH3 antibody was used as loading control. (D) Anti-myc western blot with the same samples used in B. Asterisks indicate unspecific bands. (PDF) [file pgen.1003188.s006.pdf]

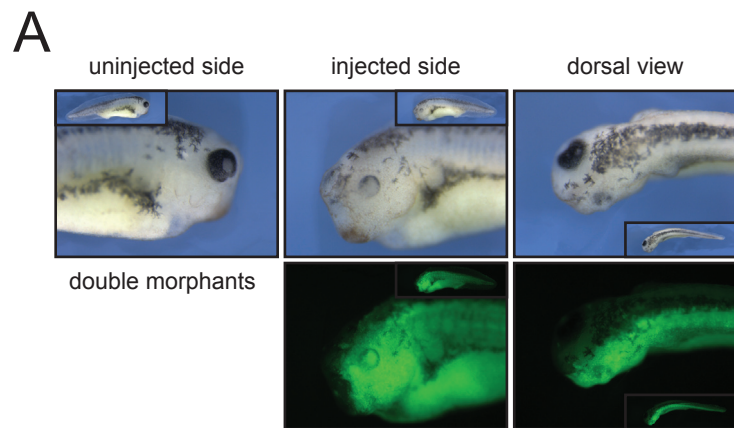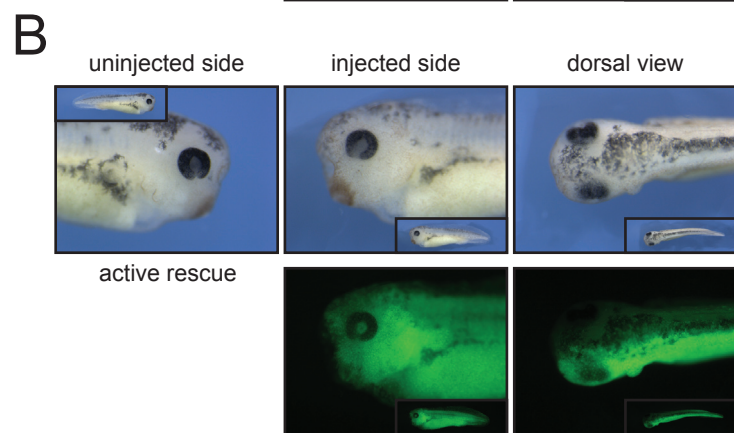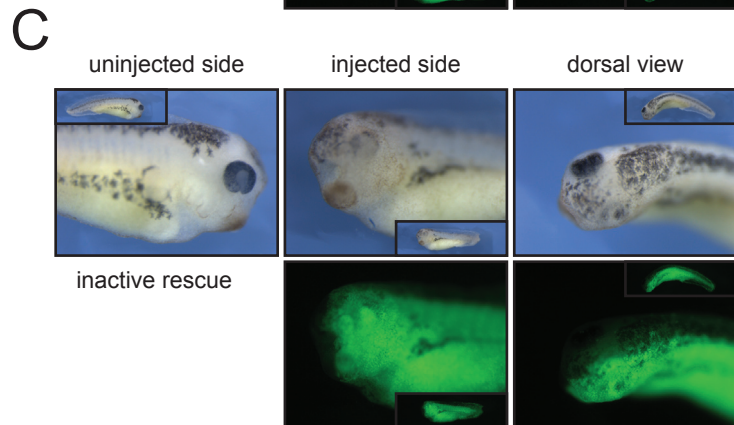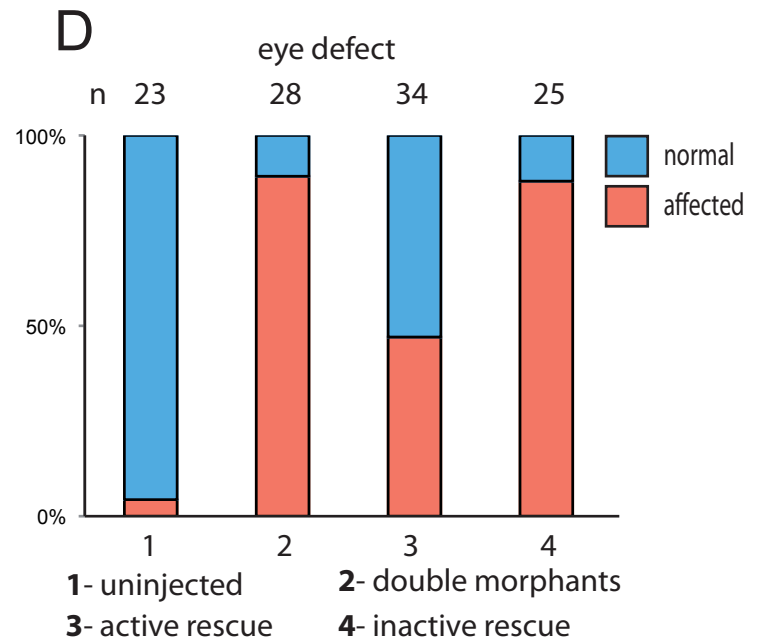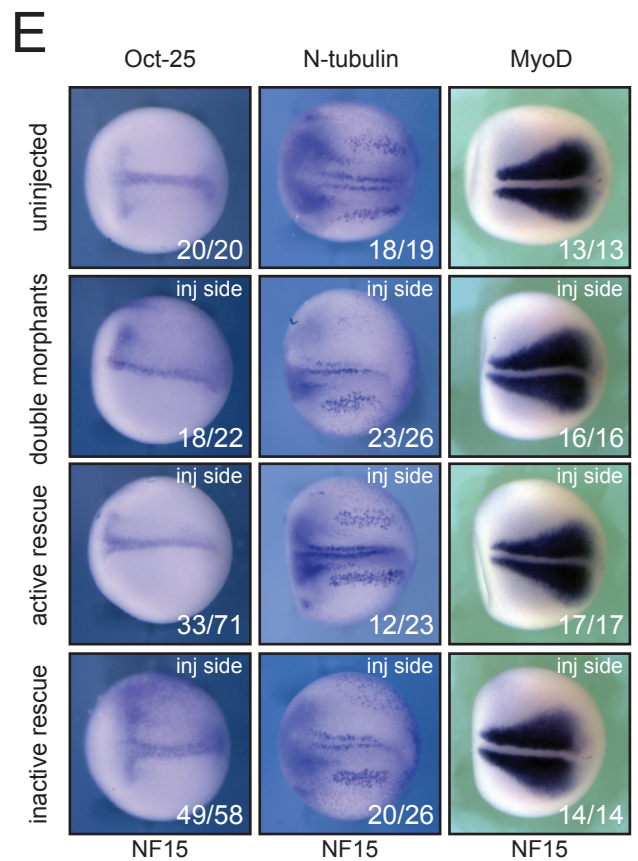

Supplement: Figure S7 — A functional SET domain is required for morphological and molecular rescue of double-morphants phenotypes. (A–C) Morphological phenotypes of NF30-33 double morphants (A), embryos injected with xSuv4-20h1, h2 morpholinos and active (active rescue, B), or inactive (inactive rescue, C) mouse Suv4-20h1, h2 mRNAs. Embryos were coinjected in one half at two cell stage with Alexa Fluor 488 Dextran as lineage tracer (green channel) to identify the injected side and sort embryos. (D) Penetrance of the eye phenotype in the indicated samples. Displayed are the results from two independent experiments. (E) RNA In situ hybridization analysis of NF15 uninjected, double morphants, active and inactive rescue embryos using probes against Oct-25, N-tubulin and MyoD. The pictures show dorsal view of stained embryos, anterior is on the left. (PDF) [file pgen.1003188.s007.pdf]

A

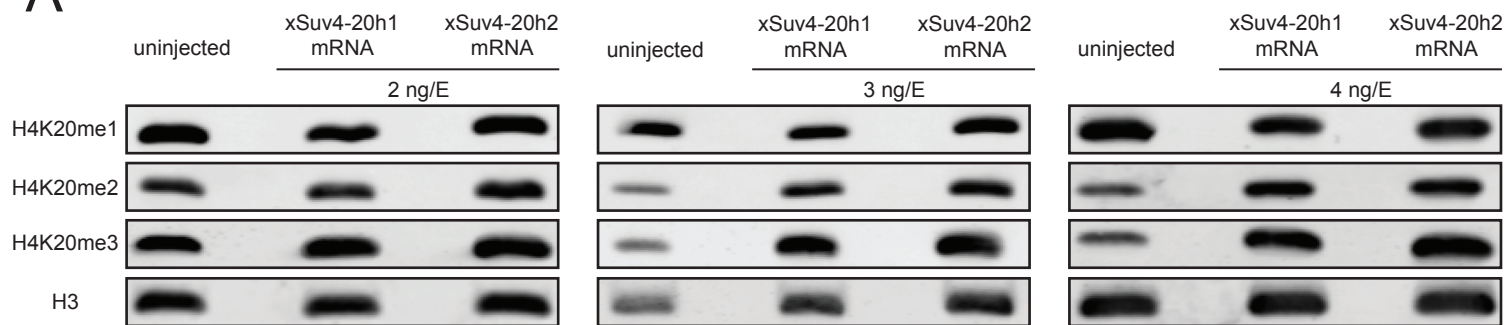

B

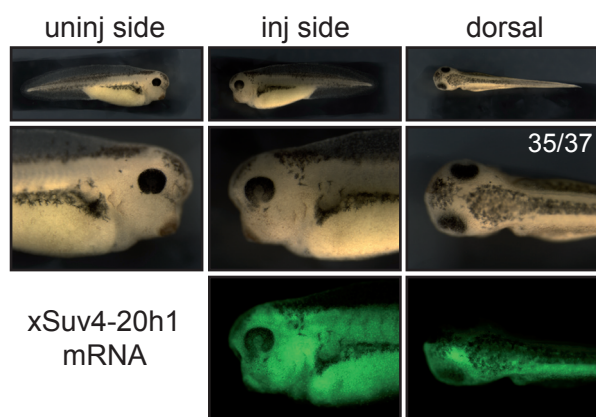

C

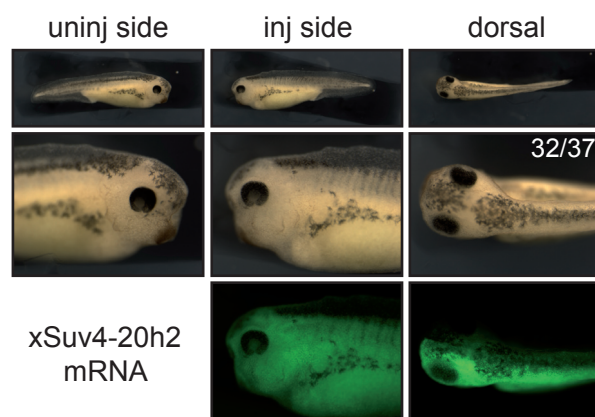

D

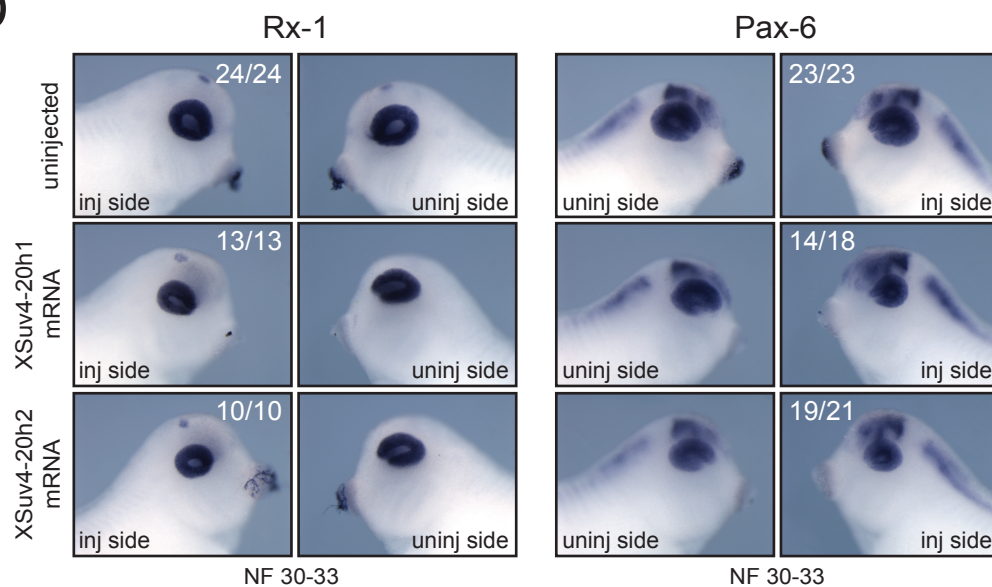

E

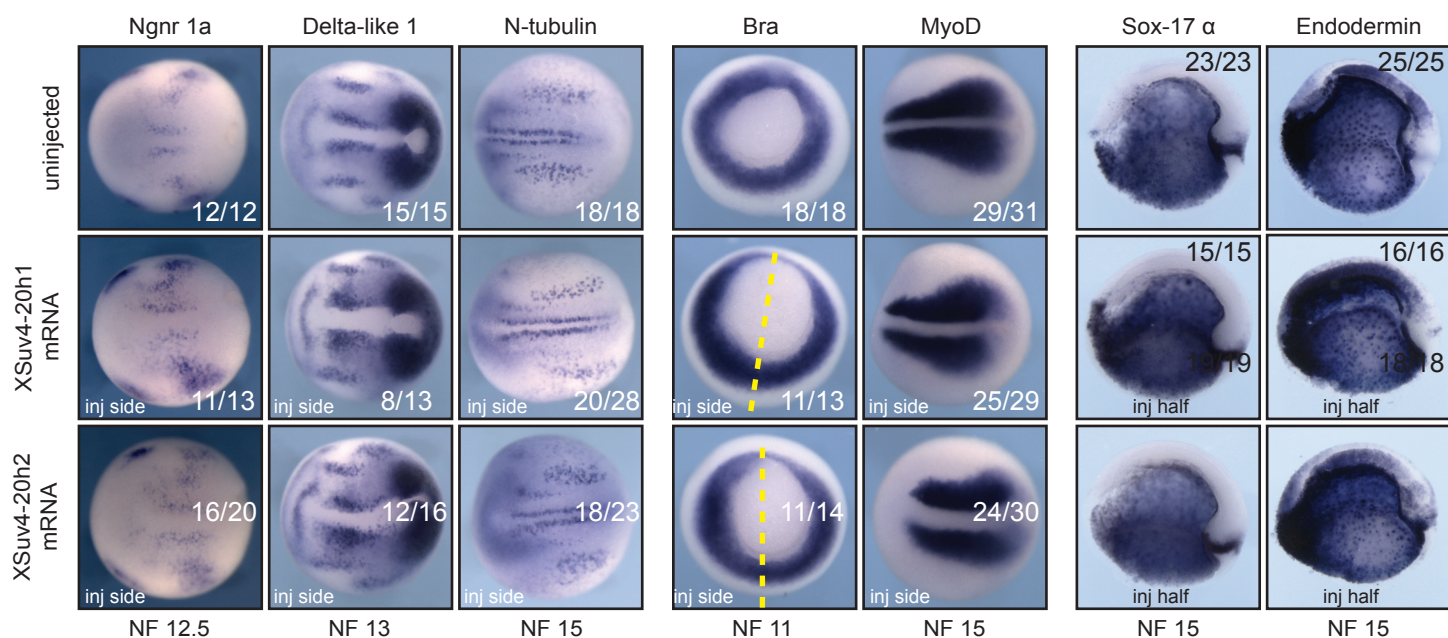

Supplement: Figure S8 — Xenopus laevis Suv4-20h1 or h2 mRNA overexpression. (A) Overexpression of frog Suv4-20h1 and h2 enzymes causes an upregulation of H4K20me2 and H4K20me3 marks. Bulk histones from uninjected embryos or embryos bilaterally injected with increasing amounts of Suv4-20h1 or h2 mRNAs were isolated at NF11.5 and analysed by Western blot. Pan H3 antibody was used as loading control. (B, C) Morphological phenotypes of NF30-33 embryos injected with xSuv4-20h1 (B) or h2 (C) mRNA. (D) RNA In situ hybridization of NF30-33 uninjected embryos (top row) and embryos injected with Suv4-20h1 (middle row) or h2 (bottom row) mRNA using probes against Rx-1 and Pax-6. Pictures show the head of stained embryos. (E) RNA In situ hybridization analysis of uninjected embryos (top row) and embryos injected with xSuv4-20h1 (middle row) or h2 (bottom row) mRNA using probes against Ngnr-1a, Delta-like 1, N-tubulin, Xbra, MyoD, Sox17 α and Endodermin. Pictures show dorsal views of stained embryos, anterior is on the left; Xbra pictures show vegetal views of NF11 embryos; MyoD pictures show dorsal views of NF15 embryos, with the head on the left. For Sox-17 α and Endodermin sagittal sections of NF15 embryos were created; pictures show internal view of the injected halves, with anterior on the left. (PDF) [file pgen.1003188.s008.pdf]

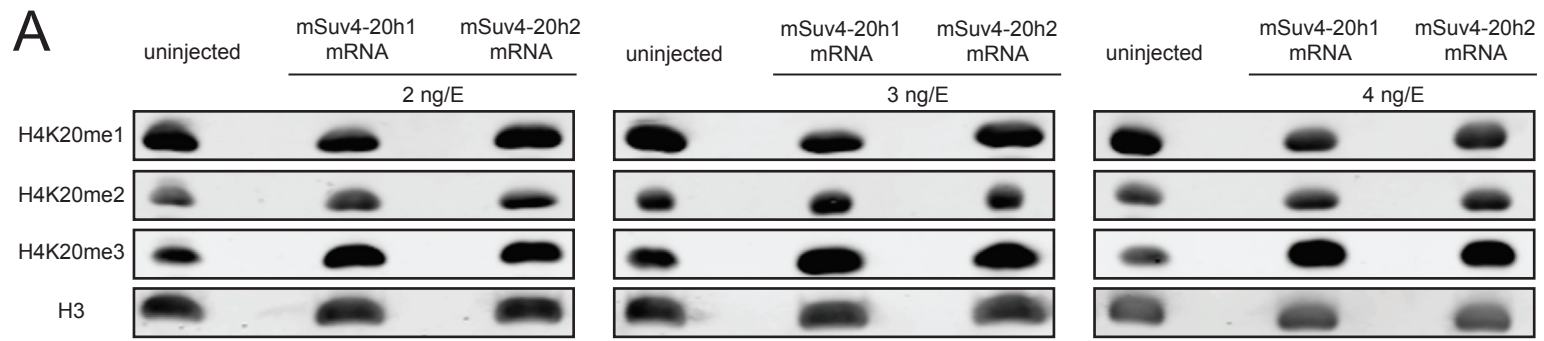

**B**

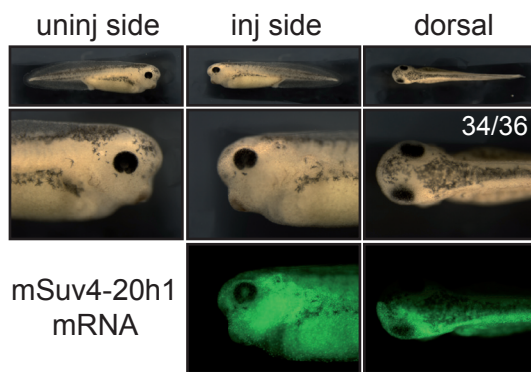

**C**

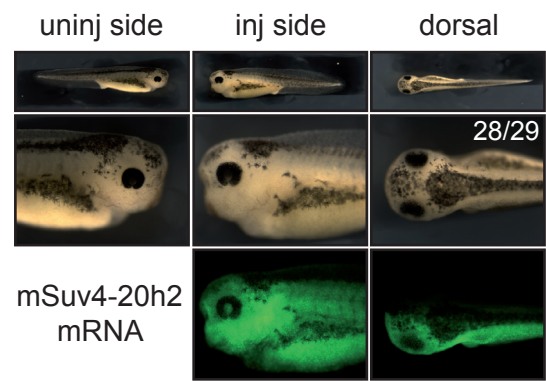

**D**

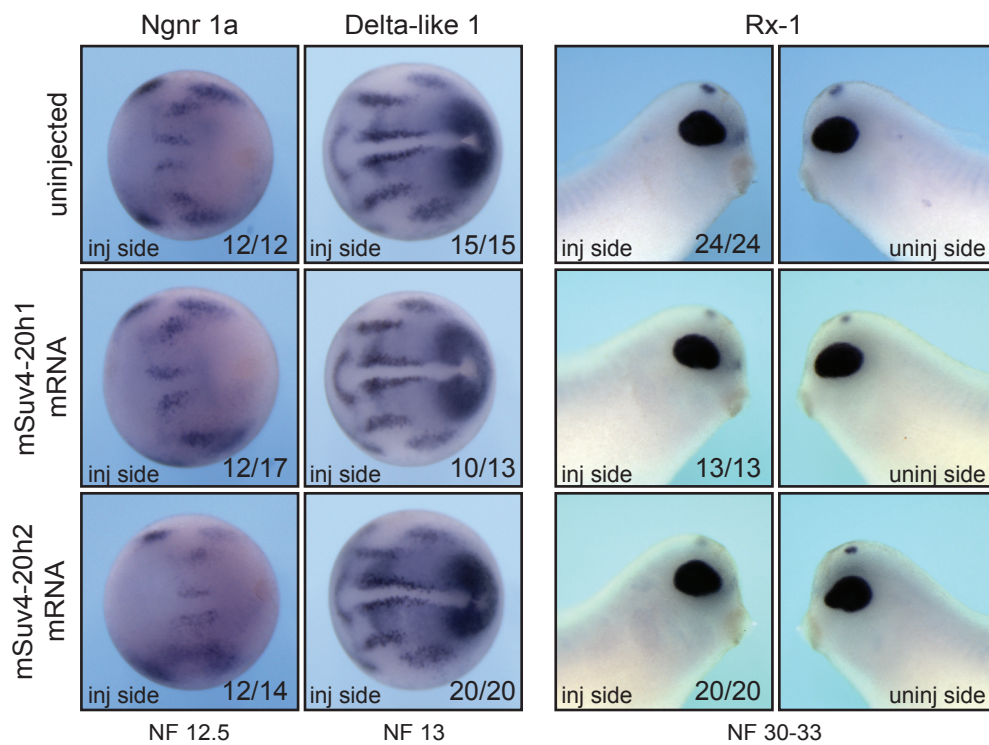

Supplement: Figure S9 — Mus musculus Suv4-20h1 or h2 mRNA overexpression. (A) Western Blot analysis of uninjected embryos or embryos injected with Mus musculus Suv4-20h1 or h2 mRNAs at different concentrations. Bulk histones from NF11.5 embryos were isolated and analyzed as described in Materials and Methods section. Pan H3 antibody was used as loading control. (B, C) Morphological phenotypes of NF30-33 embryos injected with mouse Suv4-20h1 (B) or h2 (C) mRNA. (D) RNA In situ hybridization analysis of uninjected embryos (top row) and embryos injected with mSuv4-20h1 (middle row) or h2 (bottom row) mRNA using probes against Ngnr 1a, Delta-like 1 and Rx-1. Rx-1 pictures show the head of NF30-33 stained embryos. Ngnr 1a (NF12.5) and Delta-like 1 (NF13) pictures show dorsal view of stained embryos, anterior is on the left. (PDF) [file pgen.1003188.s009.pdf]

**A**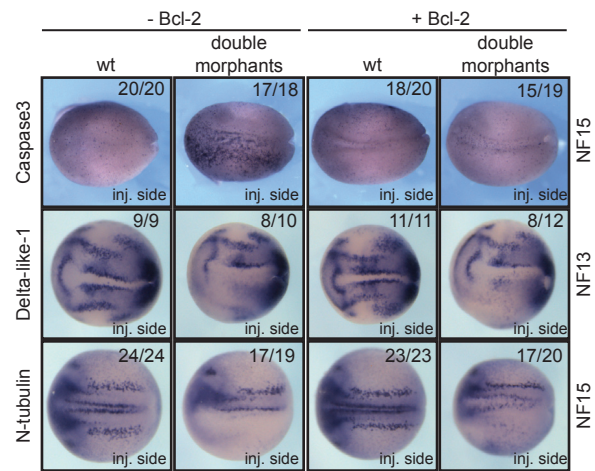**B**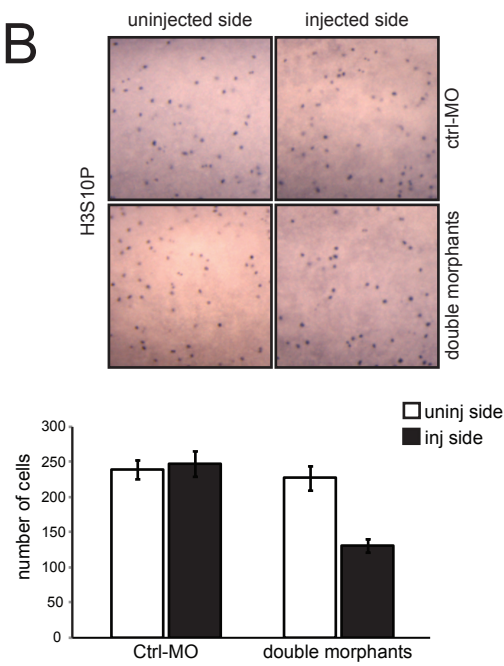

Supplement: Figure S10 — Cell proliferation and apoptosis in xSuv4-20h double morphants. (A) Double morphants show increased number of apoptotic cells during neurulation. Top row – immunocytochemistry for active Caspase3 in unilaterally injected embryos (NF15). Middle and bottom rows - RNA in situ hybridisation for Delta-like 1 (NF13) and N-tubulin mRNAs (NF15). Pictures show dorsal views, with anterior to the left. (B) Proliferation assay – immunocytochemistry for the mitotic histone modification H3S10P in Crtl-MO versus double morphant embryos. The chart shows a two-fold difference in the number of H3S10P positive cells on the injected side of double morphants. Data represent mean values of four embryos per condition from two independent experiments; error bars indicate SEM. (PDF) [file pgen.1003188.s010.pdf]

A

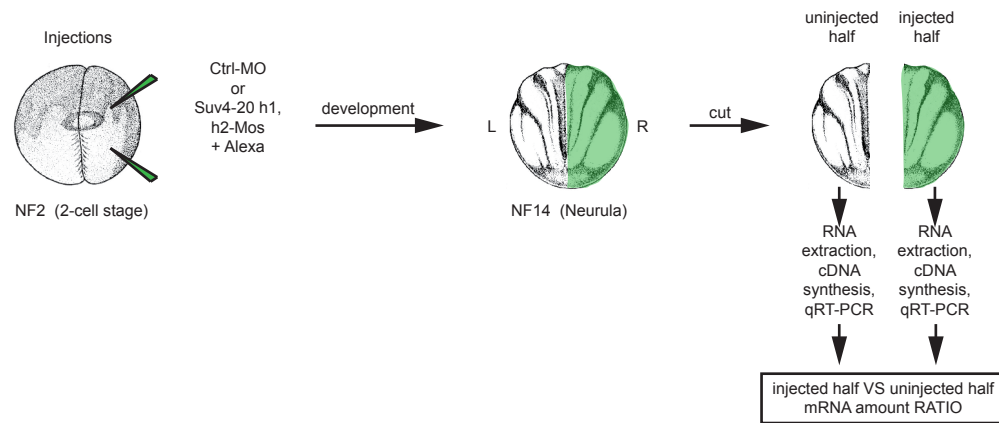

B

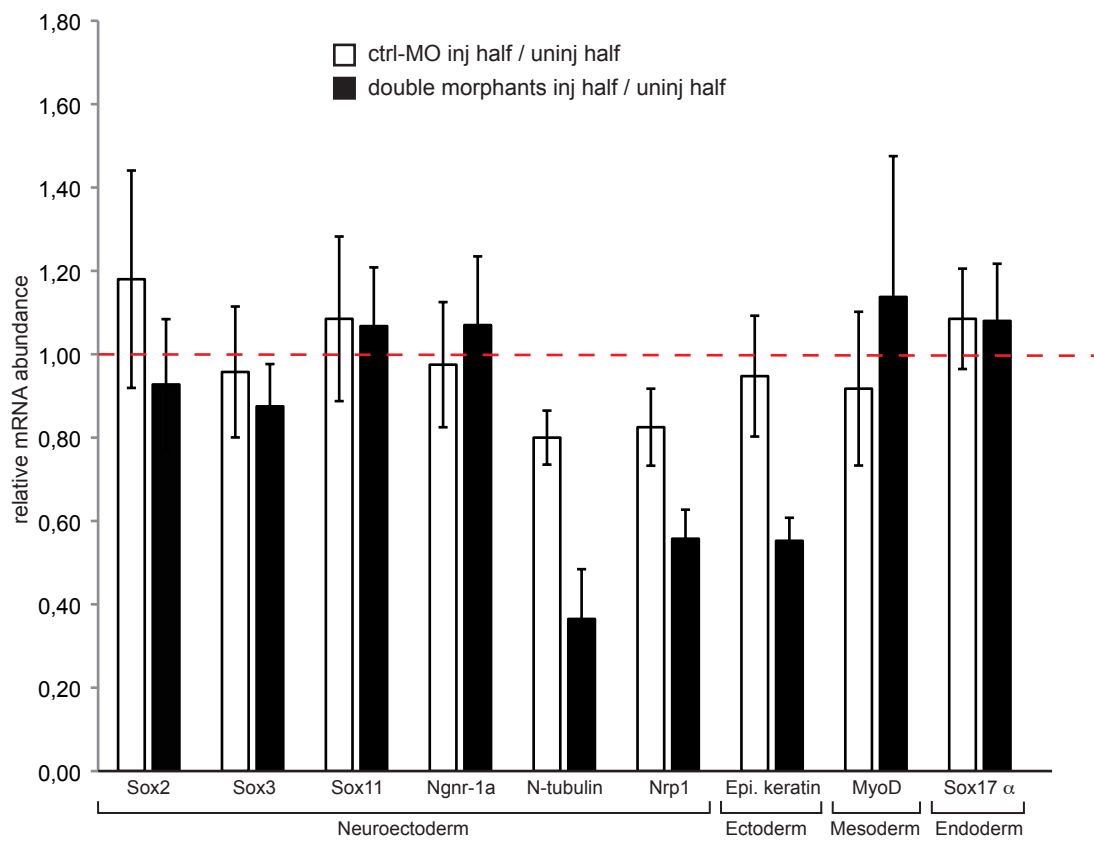

Supplement: Figure S11 — qRT-PCR analysis. (A) Schematic representation of mRNA purification from NF14-15 embryos for qRT-PCR experiments. (B) qRT-PCR profiles for the indicated genes in Ctrl-MO injected and xSuv4-20h double morphant embryos. Data represent normalized ratios of mRNA levels as means of four independent experiments, error bars indicate SEM. (PDF) [file pgen.1003188.s011.pdf]

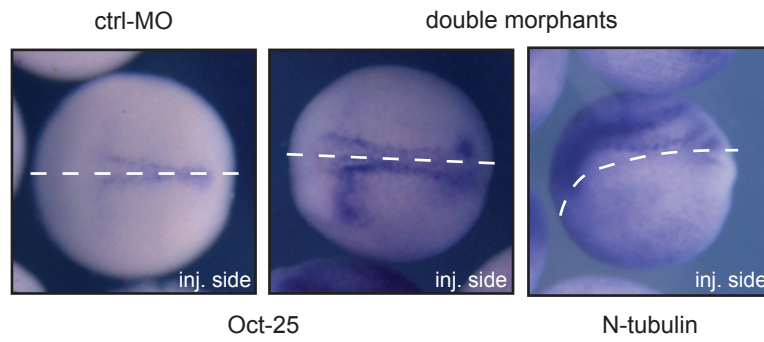

Supplement: Figure S13 — Oct-25 and N-tubulin gene expression in X. tropicalis Suv4-20h double morphant embryos. RNA in situ hybridization analysis of ctrl-MO injected and double morphant embryos at neurula stage (NF14-15) using probes against Oct-25 and N-tubulin. The pictures show dorsal views of the open neural plate with anterior to the left. Dashed line: embryonic midline. (PDF) [file pgen.1003188.s013.pdf]

**A**

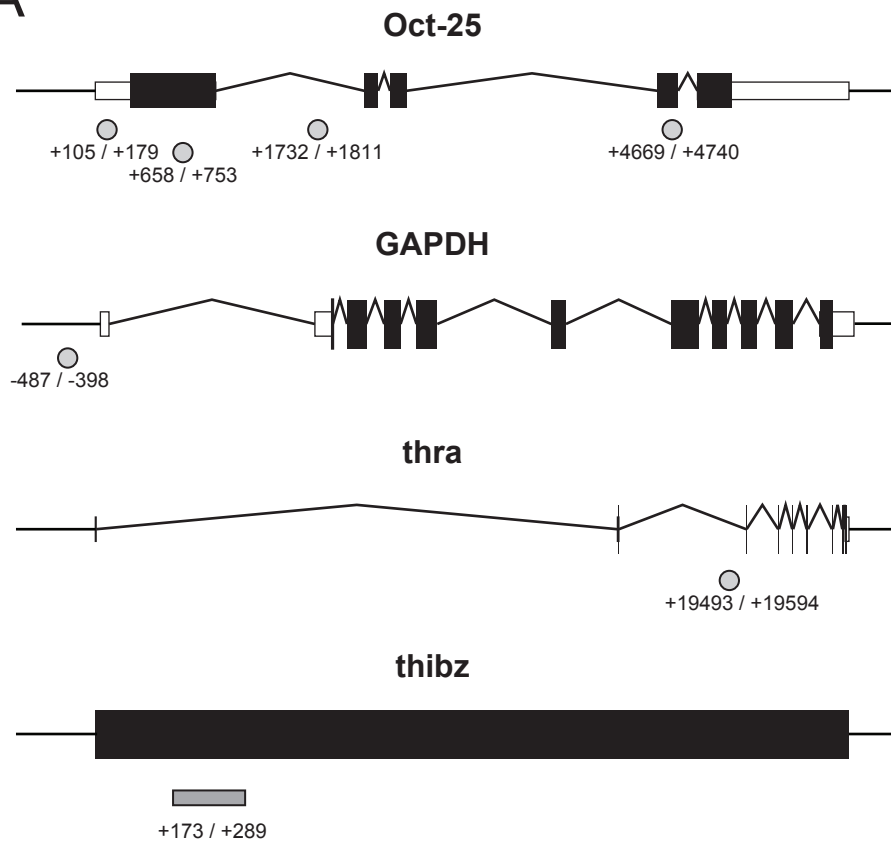

**B**

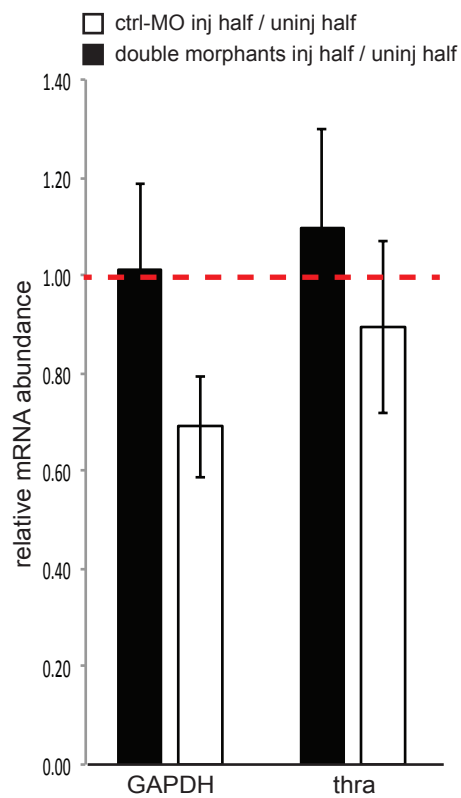

Supplement: Figure S14 — Genes analysed by ChIP. (A) Schematic representation of the genes and the amplicons analysed in ChIP experiments. Black boxes: exons; white boxes: untranslated regions; line connecting boxes: introns. For each gene the position of the amplicon(s) used in the experiments is indicated below the gene structure. (B) qRT-PCR profiles for GAPDH and thra genes in Ctrl-MO injected and xSuv4-20h double-morphant embryos. Data represent normalized ratios of mRNA levels as means of five independent experiments, error bars indicate SEM. (PDF) [file pgen.1003188.s014.pdf]

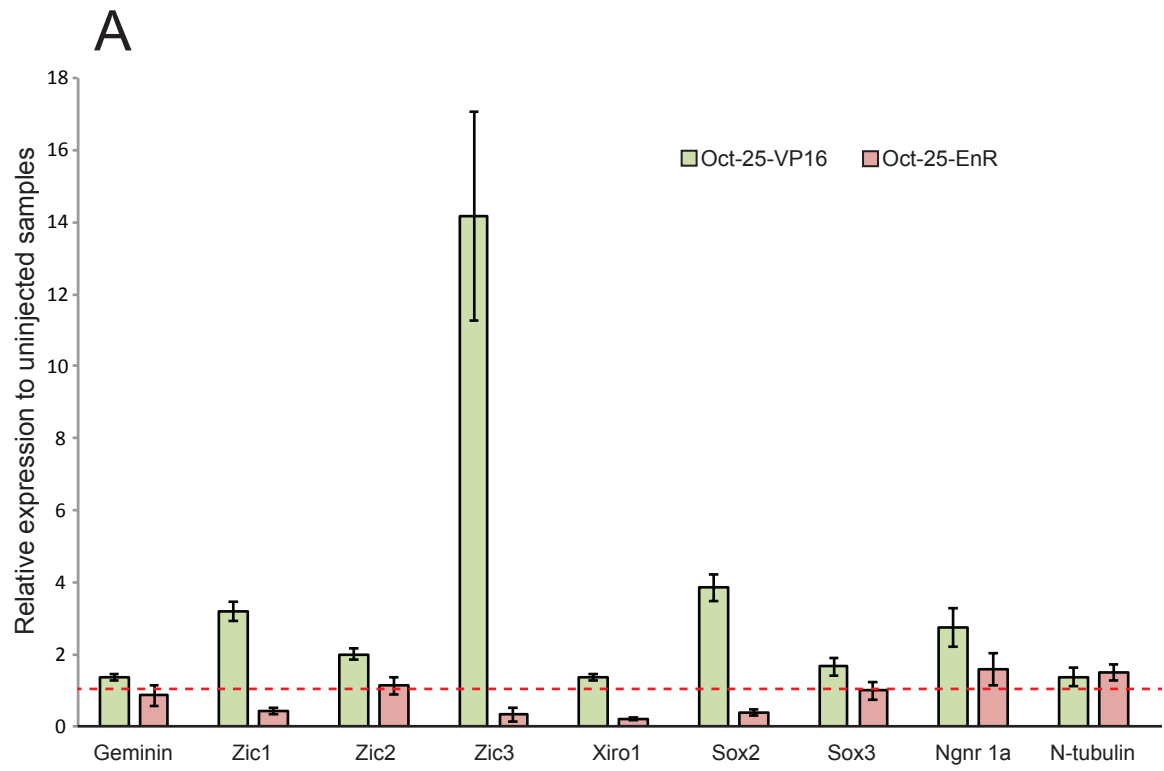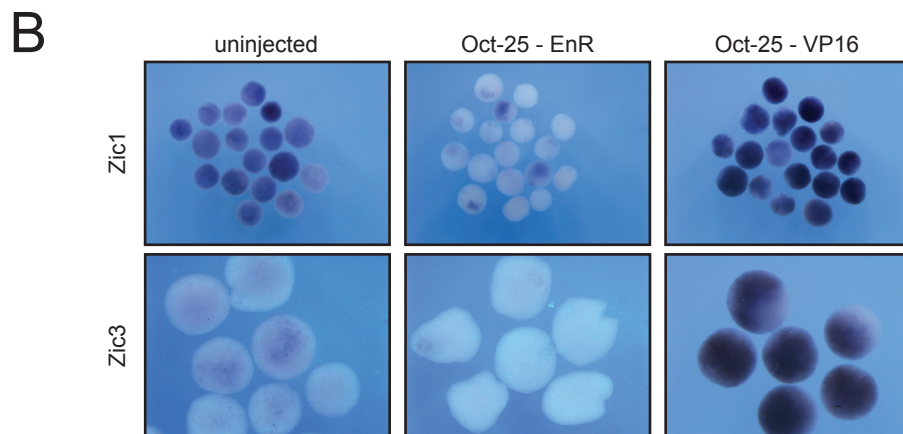

Supplement: Figure S15 — Regulation of early neural marker genes by Oct-25-VP16 and Oct-25-EnR fusion proteins. (A) qRT-PCR on animal cap (AC) explants cut from uninjected embryos and embryos overexpressing Oct-25-VP16 or Oct-25-EnR mRNAs. The chart shows the relative expression of the indicated genes compared to H4 gene levels. Data represent normalized ratios of mRNA levels as means of three or four independent experiments, error bars indicate SEM. (B) In situ hybridization on uninjected AC or explants overexpressing Oct-25-VP16/EnR for Zic1 (upper row, 20× magnification) and Zic3 genes (lower row, 50× magnification). (PDF) [file pgen.1003188.s015.pdf]

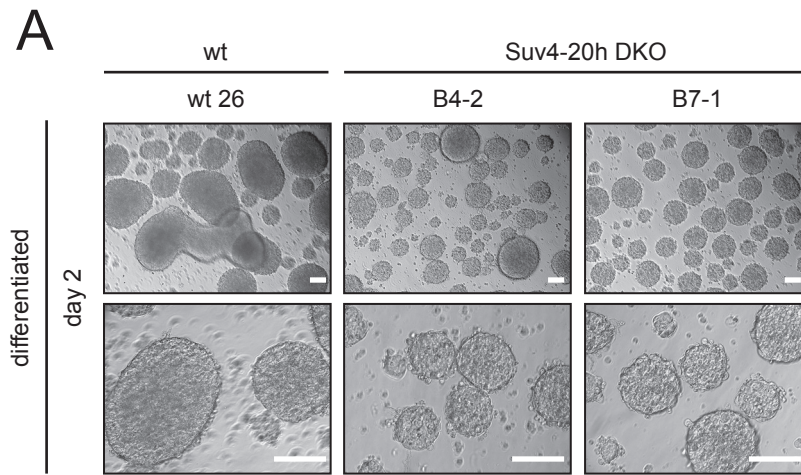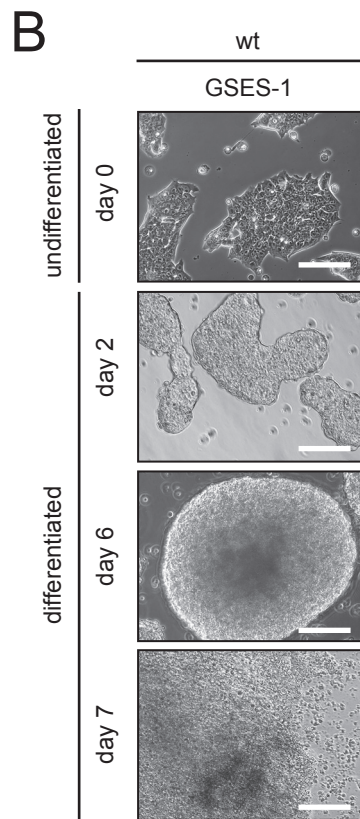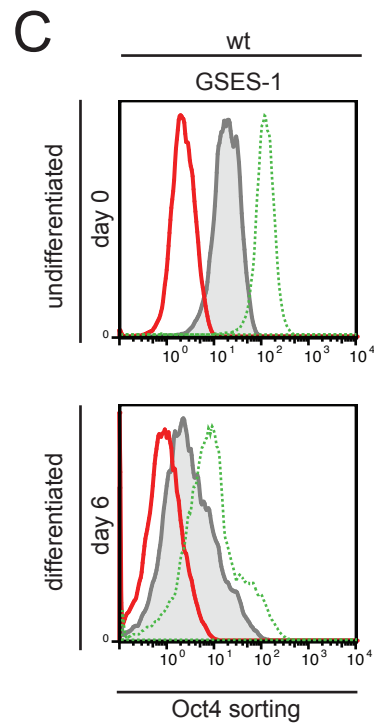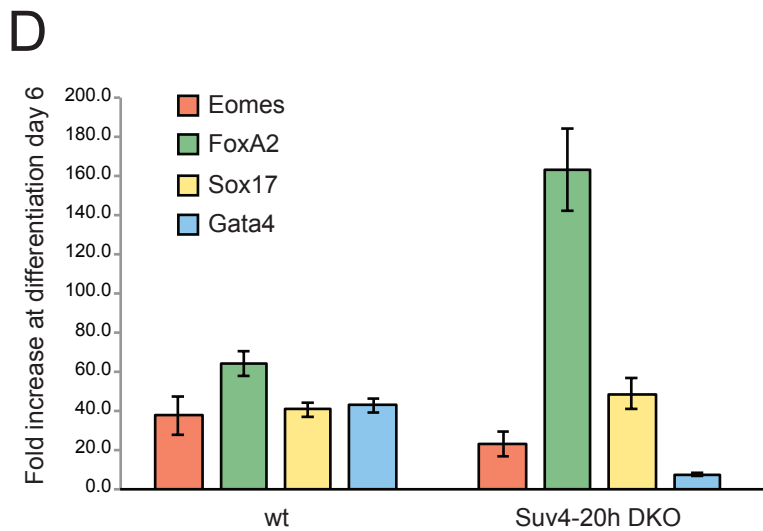

Supplement: Figure S17 — ES cell analysis. (A) Morphological appearance of differentiated day 2 embryoid bodies: B4-2 and B7-1 cell lines formed smaller bodies than those of the wild-type line wt26. (B) GSES-1 morphology and in vitro differentiation at the indicated days. Scale bar: 100 µm. (C) FACS profiles of GSES-1 cell line before (day 0) and during (day 6) differentiation. Red graphs: fluorescence of non-specific isotype control; black graphs: Oct4 protein levels in GSES-1 cells; green dashed graphs: Oct4 protein levels in mutant B7-1 cell line. (D) qRT-PCR profiles for the indicated genes in wild-type (wt) and Suv4-20h DKO cell lines at differentiation day 6. FoxA2 and Gata4 expression levels are misregulated in Suv4-20h DKO cells upon differentiation. (PDF) [file pgen.1003188.s017.pdf]
