# Supplementary material for: Suv4-20h Histone Methyltransferases Promote Neuroectodermal Differentiation by Silencing the Pluripotency-Associated Oct-25 Gene
Source: PLoS Genet. 2013 Jan 31;9(1):e1003188. doi: 10.1371/journal.pgen.1003188 (PMC3561085; doi:10.1371/journal.pgen.1003188)
Supplement: Figure S12 — Microarray analysis. (A) Schematic representing mRNA purification from NF 14–15 embryos for microarray experiments. (B) Pie-chart showing number of up- (green) and down- (red) regulated genes. (C) Histogram summarizing the fold expression change of the analysed 9752 active genes. Indicated in red are responder genes (153up, 169 down). (D) Table presenting the 10 most upregulated genes. For each gene, the gene name, symbol, the log fold change (logFC) and the fold change are indicated. (PDF) [file pgen.1003188.s012.pdf]

Nicetto_Fig.S12

A

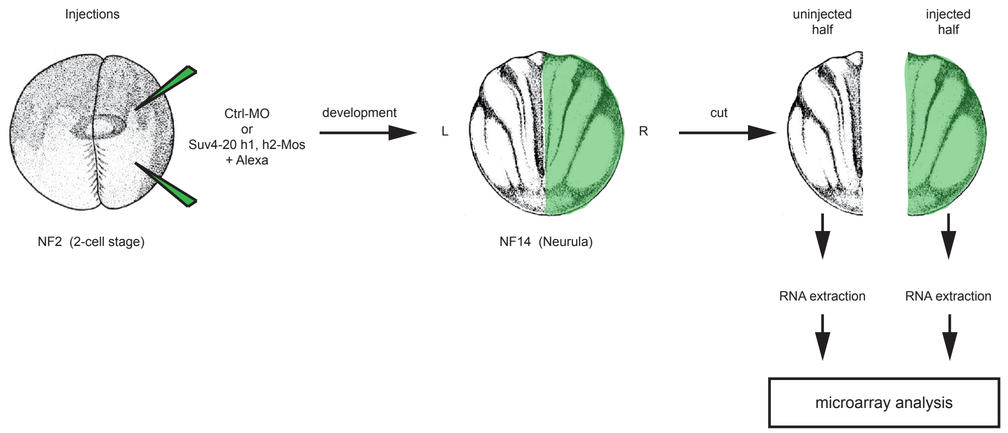

B

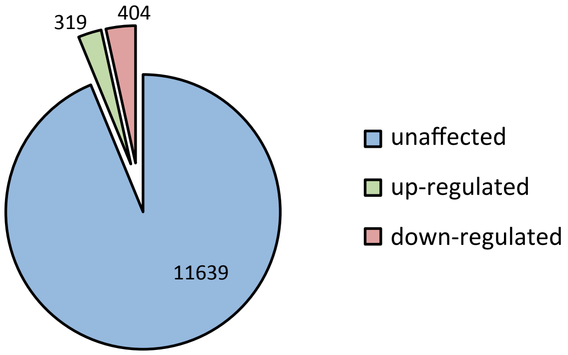

C

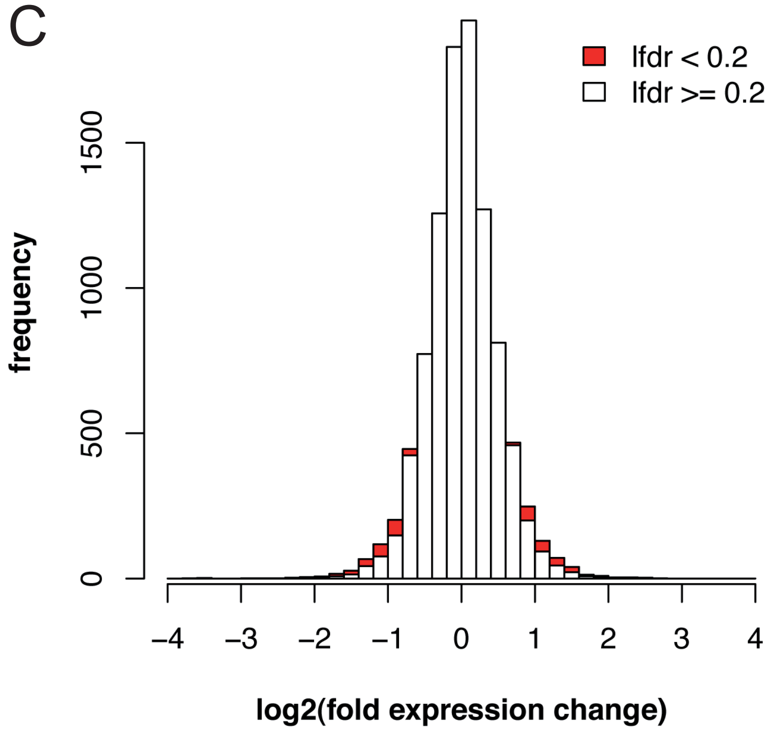

D

Top 10 Upregulated Genes			
**Gene Name**	**Gene Symbol**	**logFC**	**Fold Change**
Glucokinase (hexokinase 4)	gck	2.68077	6.41197
Rab interacting lysosomal protein	rilp	2.53108	5.78006
Tripartite motif containing 7	trim7	2.41965	5.35040
MGC81526 protein	MGC81526	2.40205	5.28555
Fat storage-inducing transmembrane protein 2	fitm2	2.23256	4.69967
Teratocarcinoma-derived growth factor 1	tdgf1	2.21938	4.65692
CDC42 small effector protein 2-c	cdc42se2-c	2.12568	4.36408
cAMP responsive element binding protein 1	creb1	2.09589	4.27490
**POU class V protein Oct-25**	**pou5f1.1**	**1.97463**	**3.93028**
Serine/threonine kinase 35	stk35	1.96083	3.89285
