# Supplementary material for: Suv4-20h Histone Methyltransferases Promote Neuroectodermal Differentiation by Silencing the Pluripotency-Associated Oct-25 Gene
Source: PLoS Genet. 2013 Jan 31;9(1):e1003188. doi: 10.1371/journal.pgen.1003188 (PMC3561085; doi:10.1371/journal.pgen.1003188)
Supplement: Figure S16 — Oct-25 binding sites on Zic1, Zic3 and Sox2 genes. Oct-25 hypothetical binding sites on Zic1, Zic3 and Sox2 have been identified as described in the Material and Methods section. The schematic representation of the genes shows: black boxes: exons; white boxes: untranslated regions; line connecting boxes: introns. For each gene the position of the binding sites, the identified sequence and the similarity to the published weight matrix (weight) are indicated in the underneath table. For Zic3 the six highest identified sequences are shown. (PDF) [file pgen.1003188.s016.pdf]

Nicetto_Fig.S16

Zic1

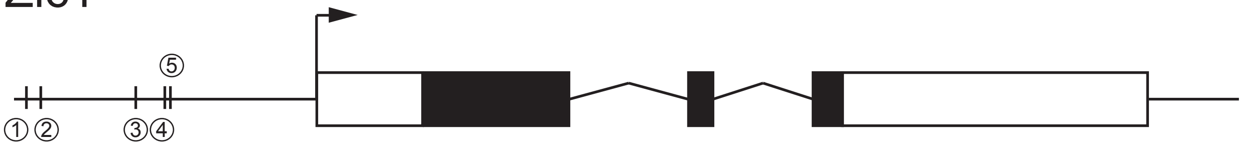

Zic1				
start	end	sequence	weight	position
-1918	-1909	AATGGAAAAA	5.2	①
-1823	-1814	AATGCAACAA	6.8	②
-1195	-1186	TATGCAAAAT	6.6	③
-1003	-994	AATGCAATGT	6.7	④
-966	-957	CATGGAAAAT	5.3	⑤

Zic3

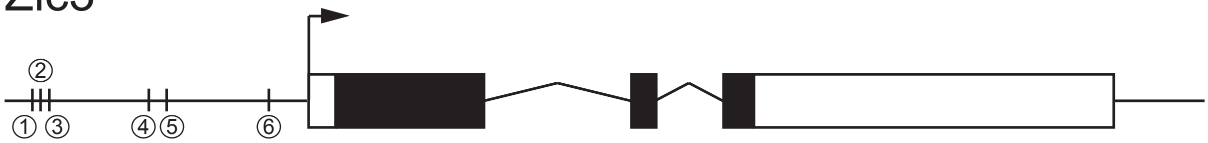

Zic3				
start	end	sequence	weight	position
-1816	-1807	AATGCAATAC	6.7	①
-1762	-1753	GATGCAAAGC	7.2	②
-1704	-1695	AATGCAAGCC	7.5	③
-1046	-1037	TATGCAAGTC	6.7	④
-928	-919	AATGCAAGAT	6.9	⑤
-252	-243	GATGCAAAAT	6.9	⑥

Sox2

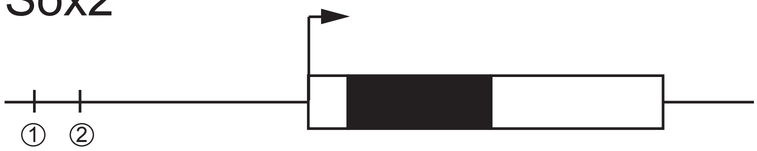

Sox2				
start	end	sequence	weight	position
-1803	-1794	CATGAAATAA	5	①
-1510	-1501	GATGTAAGAG	5.1	②
