# Supplementary material for: Suv4-20h Histone Methyltransferases Promote Neuroectodermal Differentiation by Silencing the Pluripotency-Associated Oct-25 Gene
Source: PLoS Genet. 2013 Jan 31;9(1):e1003188. doi: 10.1371/journal.pgen.1003188 (PMC3561085; doi:10.1371/journal.pgen.1003188)
Supplement: Table S1 — List of oligonucleotide sequences used in this study. (PDF) [file pgen.1003188.s018.pdf]

OLIGONUCLEOTIDES SEQUENCES	
**RT-PCR - qRT-PCR (Xenopus)**	
Geminin for	5'-tgaagtggctgttgatccag-3'
Geminin rev	5'-tcttcgttcctctgcaacct-3'
H4 for	5'-gaccgcggtcacctacacc-3'
H4 rev	5'-ctggcgcttcagaacataca-3'
MyoD for	5'-aggaaggccgccactatga-3'
MyoD rev	5'-gttgcgcaggatctccactt-3'
Ngnr 1a for	5'-acctgcactctgcgcttgat-3'
Ngnr 1a rev	5'-gcgcaaggtctcatcttgg-3'
Nrp1 for	5'-gccatgctgcaaaacttctt-3'
Nrp1 rev	5'-cccaccttatagccctccat-3'
N-tubulin for	5'-tgctgatctacgcaaactgg-3'
N-tubulin rev	5'-ctgtcagggctcggtattgt-3'
Oct-25 for	5'-caggttccagggttgcag-3'
Oct-25 rev	5'-gtccttgaggtgcaggaaag-3'
Oct-91 for	5'-ggacaacagtcgctgtagca-3'
Oct-91 rev	5'-cactgctcagcccatcacta-3'
ODC for	5'-acaaagaaacccaaaccaga-3'
ODC rev	5'-caaacaacatccagtctccaa-3'
Sox2 for	5'-tgcgtccaacaaccagaata-3'
Sox2 rev	5'-agttgtgcatcttggggttc-3'
Sox3 for	5'-atgaacggctggactaatgg-3'
Sox3 rev	5'-tacctgtgctggatctgctg-3'
Sox11 for	5'-cgagaaaatccccttcatca-3'
Sox11 rev	5'-aggatccactttgggctttttc-3'
Sox17 alpha for	5'-tactgcaactaccccagtgc-3'
Sox17 alpha rev	5'-agagcccgtccttctcaata-3'
Xiro 1 for	5'-ccataaccaccaccaccttc-3'
Xiro 1 rev	5'-tgtctgagtgcttgggactg-3'
XK81 for	5'-ccgttggtgttgaacaagtg-3'
XK81 rev	5'-gcagctcaatttccaagctc-3'
xlSuv4-20h1 for	5'-gttggcatgaagtggttgg-3'
xlSuv4-20h1 rev	5'-gcagacaatcggtttccatt-3'
xlSuv4-20h2 for	5'-ccggatgtttcttccagaga-3'
xlSuv4-20h2 rev	5'-ccaccaggagttcaatcttttc-3'
Zic1 for	5'-acagatgaggctgggcttc-3'
Zic1 rev	5'-cagttggctggaggcataat-3'
Zic2 for	5'-tcggtaggacggagcaatac-3'
Zic2 rev	5'-ttcataggggagtactggttgtg-3'
Zic3 for	5'-ggtggtgcagcctttaactc-3'

Zic3 rev	5'-tggcaaaaagtccatgttga-3'

ChIP-qPCR	
GAPDH for	5'-ctgtgctactggtgcttttcc-3'
GAPDH rev	5'-taagcacaggcagcccttac-3'
Oct-25 5'-UTR for	5'-ctccgacttatttgggtgga-3'
Oct-25 5'-UTR rev	5'-tctaacctggatgggaggtg-3'
Oct-25 exon 1 for	5'-agagtccccagaacccaaat-3'
Oct-25 exon 1 rev	5'-aagggctaccagtccatgtg-3'
Oct-25 intron 1 for	5'-aaagctaccggctgattgg-3'
Oct-25 intron 1 rev	5'-agcgtgcaggattaggtcat-3'
Oct-25 exon 4 for	5'-aggggacgctggaaagttac-3'
Oct-25 exon 4 rev	5'-ccttggctatttgcaccatc-3'
MSAT 3 for	5'-ccaccgtttgtcgtagacc-3'
MSAT 3 rev	5'-tgctggggcaattaactg-3'
Thibz for	5'-gctgtcggaactctcactcc-3'
Thibz rev	5'-gcgtctcttgtcccagtagc-3'
Thr alpha for	5'-atttgctttcatgccttgct-3'
Thr alpha rev	5'-tatgaaacggagcgacacaa-3'

mutagenesis PCR	
mSuv4-20h1 Y299A for	5'-cctggagaagaaatttcttgttacgcaggagatggctttttggagaaa-3'
mSuv4-20h1 Y299A rev	5'-tttctccaaaaaagccatctcctgcgtaacaagaaatttcttctccagg-3'
mSuv4-20h2 Y217A for	5'-ggatgaagtgacttgcttcgcaggtgagggcttcttcgg-3'
mSuv4-20h2 Y217A rev	5'-ccgaagaagccctcacctgcgaagcaagtcacttcatcc-3'
mSuv4-20h1 N264A for	5'-ggctcggtcctgctgcatttatagcccatgattgcagacctaactg-3'
mSuv4-20h1 N264A rev	5'-cagttaggtctgcaatcatgggctataaatgcagcaggaccgagcc-3'
mSuv4-20h2 N182A for	5'-ggcccagctgccttcatcgcccatgactgcaaaccc-3'
mSuv4-20h2 N182A rev	5'-gggtttgcagtcatgggcgatgaaggcagctgggcc-3'

qRT-PCR (ES cells)	
Actin for	5'-ggtcatcactattggcaacg-3'
Actin rev	5'-tccatacccaagaaggaagg-3'
Eomesodermin for	5'-atcgaccataacccccttcgcc-3'
Eomesodermin rev	5'-cgtaccgacctccagggacaac-3'
FoxA2 for	5'-gtgaagatggaagggcacgagc-3'
FoxA2 rev	5'-gccgcggacatgctcatgta-3'
GAPDH for	5'-tcaagaaggtggtgaagcag-3'
GAPDH rev	5'-gttgaagtcgcaggagacaa-3'
Gata4 for	5'-agggtgagcctgtatgtaatgc-3'
Gata4 rev	5'-attcaggttcttgggcttcc-3'

Sox17 for	5'-acgcaagcggttggcacag-3'
Sox17 rev	5'-cgaagggccgcttctctgc-3'
