# Supplementary material for: Suv4-20h Histone Methyltransferases Promote Neuroectodermal Differentiation by Silencing the Pluripotency-Associated Oct-25 Gene
Source: PLoS Genet. 2013 Jan 31;9(1):e1003188. doi: 10.1371/journal.pgen.1003188 (PMC3561085; doi:10.1371/journal.pgen.1003188)
Supplement: Text S1 — Supporting information on experimental procedures. This file contains additional information on statistical analysis, extraction of Myc-tagged fusion protein from embryos, qRT-PCR samples preparation, Vibratome sections of Oct-25 stained embryos, Immunostaining, Immunofluorescence microscopy of MEF cells and ChIP (chromatin immunoprecipitation) analysis. (DOC) [file pgen.1003188.s019.doc]

**SUPPLEMENTARY INFORMATIONS ON EXPERIMENTAL PROCEDURES**

**Statistical analysis**

The statistical analysis was performed using two-tailed, Paired Student’s *t*-test, unless differently specified.

**Myc-tagged fusion protein extraction from embryos**

25 embryos per condition were lysed in 100μl of 100mM NaCl, 10mM Tris pH 7.5 buffer supplemented with 1mM NaF, 20mM beta-glycerol, 0.1mM Sodium Vanadate, 10mM Na Butyrate, 0,5% NP-40 and EDTA-free protease inhibitor cocktail tablets (Roche). Embryos were centrifuged 15min at 14,000g at 4 °C; the supernatant was collected and 2X Loading buffer (Roti-Load1; carlroth.de) was added. Samples were subsequently analysed by western blot.

**qRT-PCR samples preparation**

Two-cell stage embryos were injected with ctrl-MO (80ng) or xSuv4-20h1 and h2 morpholinos (40ng each) mixed with Alexa 488 in only one blastomere only. At neurula stage (NF15), injected embryos were cut along the midline into pools of injected and uninjected halves based on alexa fluorescence. As control, embryos unilaterally injected only with Alexa 488-Dextran were processed in parallel. Six halves each from corresponding embryos were pooled into injected and uninjected sample pairs and used for RNA extraction. RNA samples from two independent experiments were subjected to microarray analysis, while RNA samples from four independent replicates were used to perform qRT-PCR analysis. For qRT-PCR profiles of ES cell lines, 10^6 cells at day 0 and embryoid bodies at day 6 of differentiation were harvested and stored at -80°C. Total cellular RNA was extracted from independent experiments. For both frog and mouse samples, cDNA synthesis was performed using DyNAmo cDNA synthesis kit (Finnzymes), following the manufacturers’ protocol. For microarray analysis, cDNA probes were prepared according to standard Affimetrix protocol. For each experiment a control RNA aliquot was processed without reverse transcriptase (–RT sample). Real time PCR was performed using the Power SYBR Green PCR Master mix and run in LightCylcer**®** 480 System (Roche). Primer sequences are listed in Supplement Table S1. C(t) values for each sample were normalized to histone H4 as reference gene. The fold change between samples was then calculated by normalizing ctrl-MO injected embryos or xSuv4-20h double-morphant embryos to the uninjected samples by the ΔΔC(t) method. Finally the ratio between injected and uninjected side within each sample was calculated to estimate the up- or downregulation of a gene’s mRNA level. For qRT-PCR profiles of ES cell lines, C(t) values for each sample were normalized to the two reference genes, GAPDH and Actin. ΔΔC(t) method was applied to calculate fold change difference between samples.

**Vibratome sections of Oct-25 stained embryos**

Embryos were rinsed in gelatine/albumin mixture (2.2g of gelatine dissolved in 500ml 1X PBS subsequently supplemented with 135g of albumin (Roth) and 90g of Sucrose). 1/20 vol of glutaraldehyde were added to 2ml of albumin/gelatine mixture. The solution was quickly vortexed and poured in a small plastic tray to create a bottom layer. Embryos were placed and properly oriented on the solidified layer. A second layer of albumin/gelatine mixture plus glutaraldehyde was prepared and poured on the embryos. The mixture was hardened at least for 30min. The gelatinized block with embedded embryos was cut out under a dissecting microscope and glued onto a metal support. 30-50μm sections were created using a Vibrotome 1000 (Technical Products International, INC.). Sections were transferred on slides, slightly dried, covered with X-TRA Kit mounting medium (Medite) and analysed with Leica M205FA Fluorescence Stereomicroscope.

**Immunostaining**

Immunostaining was performed according to Sive et al. (2000). Chromogenic reactions with BCIP/NBT (biomol) were stopped by rinsing embryos in PBS. Embryos were refixed in MEMFA and bleached in 1% H_2_O_2_, 5% Formamid, 0.5X SSC on a light box for at least 4 hours. For immunostaining of paraffin embedded samples, embryos were fixed in MEMFA for one hour at room temperature and then transferred in ice-cold Dent’s Fixative o/n at -20°C. Prior embedding embryos were rehydrated for 30min in 100mM NaCl, 100mM Tris/HCl pH 7.4. After dehydration with increasing ethanol concentrations, embryos were incubated for two hours in Xylene. Subsequently embryos were soaked in paraffin at 55°C twice for two hours, followed by proper orientation in moulds and paraffin hardening on cooling plates. Embryos were sectioned into slices of 10μm, which were dried on glass slices for 2 hours at 37°C. Paraffin was removed washing the samples twice with X-tra Solv (Medite), then with decreasing ethanol concentration and finally with 1X PBS. Heat-induced epitope retrieval was performed incubating the slides in citrate buffers solution for 1 hour at 90°C followed by cooling down to room temperature. Endogenous peroxidase inactivation was achieved by 10min incubation with 3% peroxidase inactivating solution (35% hydrogen peroxidase, Roth, 1/10 Methanol in PBS). Unspecific antibody binding sites were blocked by incubation for 1 hour with 2% biotin-free albumin (Roth) in PBS. Primary antibodies, diluted in blocking-solution, were incubated o/n at 4°C. Secondary antibody incubation was preceded by washes in PBST (1X PBS + 0.1% Tween20); subsequently, slices were incubated 1 hour at room temperature with biotinylated anti-Rabbit secondary antibody. After several washes in PBST, slices were incubated for 1 hour in the dark at room temperature in High Sensitivity Streptavidin-HRP solution (Thermo Scientific), diluted 1:500 in blocking solution. Staining was stopped after about 10min at room temperature in DAB substrate chromogen solution by washing the samples in double distilled water. For counterstaining haemalaun (Roth) was used (6 min at room temperature in 1:3 haemalaun-solution); slices were then blued with 10min under running tap water. Increasing ethanol concentrations and X-tra Solv were used for dehydration. Finally slides were embedded using X-TRA Kit mounting medium (Medite) and analyzed with Leica DM microscope.

**Immunofluorescence microscopy of MEF cells**

Wt or Suv4-20 DKO MEFs were fixed with 3.7% (w/v) paraformaldehyde in PBS for 10min at room temperature, PBS washed and permeabilized in 0.1% (w/v) NaCitrate containing 0.1% (v/v) Triton X-100 for 5min. After washing in PBS-T (PBS, 0.1% [v/v] Tween-20), cells were blocked for 30min in blocking buffer (PBS, 2.5% [w/v] BSA, 0.1% [v/v] Tween-20) at room temperature. The cells were incubated with primary antibody (H4K20me3, diluted in blocking buffer; ) overnight at 4°C. After washing with PBS-T, the secondary antibody (Cy3-conjugated anti-rabbit; Jackson ImmunoResearch, diluted in PBS-T) was added for 1 hour at room temperature. After several washes in PBS-T, the cells were embedded in Vectashield containing DAPI (Vector Laboratories) and stored at 4°C for further analysis.

**Chromatin Immunoprecipitation experiments**

Aliquots of 50 *Xenopus tropicalis* injected and uninjected embryos were fixed at NF 14-15 in 5ml 1% formaldehyde in PBS for 5min at 20°C on a rolling wheel. Crosslinking was stopped by a 10min wash with 0.125M glycine/PBS, followed by three washes in PBS. Fixed embryos were transferred in 1.5ml eppendorf tubes, frozen in liquid nitrogen and stocked at -80°C. At experimental day1, embryos were thawed for 15min on ice.

Two 15ml conical tubes of blocked protein-G and –A (Protein-A and –G Sepharose 4, Fast Flow, GE Healthcare) beads were prepared by incubating the proper amount of beads (plus an extra 50μl) with 15ml of 5% BSA in PBS. The tubes were incubated at 4 °C while mixing for at least 1 hour.

Two 50-embryos aliquots (100 embryos/condition) were used in each experiment. 600μl of 4°C RIPA buffer was added to each 50 embryos aliquot. Samples were homogenized with a pellet pestle by gently disrupting the embryos until no large embryo fragments are visible. Embryos were incubated on ice at least 10min and subsequently centrifuged at 14,000rpm for 10min at 4°C. The supernatant was discarded and the wall of the tubes was wiped with a kimwipe to remove lipid residue. 650μl of 4°C RIPA buffer was added to each sample; the pellet was re-homogenized vigorously. Samples were subsequently sonicated using the Bioraptur (Diagenode) for 25 cycles each composed by 30sec pulse and 30sec rest. Sampels were centrifuged at 14,000rpm for 10min at 4°C. 600μl sheared chromatin from the two 50-embryos aliquots per sample were pooled together and transferred into a pre-chilled, clean 1.5 microcentrifuge tube. Input samples were then prepared: combining 5μ of sheared chromatin plus 195μl TES . Input sample was frozen at -80°C and processed once the immunoprecipitations were completed.

One of the two 15ml conical tubes containing the blocked protein-G and –A beads was centrifuged at 1000rpm for 5min at 4°C. Excess of 5% BSA/PBS was removed and the beads were gently resuspended by pipetting. Pre-clearing step was achieved by dispensing 50μl blocked beads to each sample of sheared chromatin and incubating each sample at 4°C with mixing for 1-1.5 hour. Samples were subsequently centrifuged at 1000rpm for 1min at 4°C. Each 1.2ml sheared chromatin sample was separated into two samples by transferring 580μl of pre-cleared, sheared chromatin in two new 1.5ml pre-chilled, clean microcentrifuge tubes. Each new tube was filled with RIPA buffer and the immunoprecipitation was achieved by adding the appropriate amount of antibody to only one of the two tubes, keeping the second one as negative control. Samples were incubated overnight at 4°C with mixing.

At experimental day2 the second 15ml conical tube containing the blocked protein-G and –A beads was centrifuged at 1000rpm for 5min at 4°C. Excess 5% BSA/PBS was removed, the beads were gently resuspended by pipetting. 50μl blocked beads was added to each sample. Samples were incubated at 4°C with mixing for 1.5 hour and afterwards centrifuged at 100rpm for 1min at 4°C. Beads were subsequently washed: each wash consisted of a 1-minute spin at 1000rpm in a 4°C centrifuge to pellet the immunocomplexes, removal of supernatant with a 20-gauge needle, addition of 1ml wash buffer and incubation at 4°C with mixing for 5min. Samples were washed for a total of 8 times, using 2 washes each with buffers I through IV. Following the washes, the supernatant was aspirated with a 26-gauge needle inserted into the beads to completely remove any residual wash buffer. 200μl TES buffer was added to the beads. Elution was achieved by incubating the samples at 65°C for 1 hour in a table shaker (1000rpm). During this time the frozen input samples were thawed and vortexed to resuspend any precipitated SDS. All the different samples (input, IP and negative control) were processed in the same manner for the rest of the procedure.

After elution samples were centrifuged at 14,000rpm at RT for 1min. 200μl of the eluted supernatant was transferred to a new 1.5ml microcentrifuge tube. RNase treatment was achieved by adding 2μl of 10 mg/ml stock RNase A (Quiagen) to wach samples and by incubating the samples for 45min at 37°C. Subsequently, 12μl of Proteinase K/Glycogne solution was added to each sample. Samples were incubated at 68°C for 4 hours while shaking (1300rpm) to reverse crosslinks and digest proteins. DNA was purified on column using the QIAquick® PCR purification kit (Qiagen) following the manufacturer’s protocol. DNA was eluted in 33μl of EB buffer. qPCR was performed using the Light Cycler 480 System (Roche). Data were analysed using the Light Cycler 480 Software Release 1.5.0 SP1 (Roche).
